# Genome Wide Mapping of Peptidases in *Rhodnius prolixus*: Identification of Protease Gene Duplications, Horizontally Transferred Proteases and Analysis of Peptidase A1 Structures, with Considerations on Their Role in the Evolution of Hematophagy in Triatominae

**DOI:** 10.3389/fphys.2017.01051

**Published:** 2017-12-12

**Authors:** Bianca S. Henriques, Bruno Gomes, Samara G. da Costa, Caroline da Silva Moraes, Rafael D. Mesquita, Viv M. Dillon, Eloi de Souza Garcia, Patricia Azambuja, Roderick J. Dillon, Fernando A. Genta

**Affiliations:** ^1^Laboratory of Insect Physiology and Biochemistry, Oswaldo Cruz Institute – Oswaldo Cruz Foundation (IOC-FIOCRUZ), Rio de Janeiro, Brazil; ^2^National Institute of Science and Technology for Molecular Entomology (INCT-EM), Cidade Universitária, Rio de Janeiro, Brazil; ^3^Chemistry Institute, Federal University of Rio de Janeiro, Rio de Janeiro, Brazil; ^4^Institute of Integrative Biology, University of Liverpool, Liverpool, United Kingdom; ^5^Division of Biomedical and Life Sciences, Lancaster University, Lancaster, United Kingdom

**Keywords:** *Rhodnius prolixus*, insect, genome, blood feeding, peptidase, gene transfer

## Abstract

Triatominae is a subfamily of the order Hemiptera whose species are able to feed in the vertebrate blood (i.e., hematophagy). This feeding behavior presents a great physiological challenge to insects, especially in Hemipteran species with a digestion performed by lysosomal-like cathepsins instead of the more common trypsin-like enzymes. With the aim of having a deeper understanding of protease involvement in the evolutionary adaptation for hematophagy in Hemipterans, we screened peptidases in the *Rhodnius prolixus* genome and characterized them using common blast (NCBI) and conserved domain analyses (HMMER/blast manager software, FAT, plus PFAM database). We compared the results with available sequences from other hemipteran species and with 18 arthropod genomes present in the MEROPS database. *Rhodnius prolixus* contains at least 433 protease coding genes, belonging to 71 protease families. Seven peptidase families in *R. prolixus* presented higher gene numbers when compared to other arthropod genomes. Further analysis indicated that a gene expansion of the protease family A1 (Eukaryotic aspartyl protease, PF00026) might have played a major role in the adaptation to hematophagy since most of these peptidase genes seem to be recently acquired, are expressed in the gut and present putative secretory pathway signal peptides. Besides that, most *R. prolixus* A1 peptidases showed high frequencies of basic residues at the protein surface, a typical structural signature of Cathepsin D-like proteins. Other peptidase families expanded in *R. prolixus* (i.e., C2 and M17) also presented significant differences between hematophagous (higher number of peptidases) and non-hematophagous species. This study also provides evidence for gene acquisition from microorganisms in some peptidase families in *R. prolixus*: (1) family M74 (murein endopeptidase), (2) family S29 (Hepatitis C virus NS3 protease), and (3) family S24 (repressor LexA). This study revealed new targets for studying the adaptation of these insects for digestion of blood meals and their competence as vectors of Chagas disease.

## Introduction

The arthropod development requires several nutritional components that are usually obtained through the digestive function. In this process, enzymes are crucial to break down complex compounds into simpler molecules that can be absorbed by the organism to be used for energy, growth, or reproduction. The molecular properties of these enzymes differ among arthropod taxa and are especially correlated to their phylogenetic background. However, other factors such as the adaptation to new feeding resources may have influenced the pre-established composition of enzymes in closely related organisms (Terra and Ferreira, 2005).

Hematophagy (i.e., ability in feeding on vertebrate blood) potentiates the ingestion of high amounts of proteins in short time by arthropods. This feeding behavior has emerged independently in several arthropod groups, particularly in females that require higher amounts of proteins to produce their offspring (Black and Kondratieff, 2004; Attardo et al., 2005; Grimaldi and Engel, 2005). However, hematophagy brings several challenges to insects, due to an increase of contact with vertebrates that may present predatory behavior or defense mechanisms, and blood parasites that are potential pathogens (Devenport and Jacobs-Lorena, 2004; Balenghien et al., 2011). In addition to that, the heme molecule released by digestion of hemoglobin in hematophagous is toxic and result in oxidative stress to the midgut cells (Graça-Souza et al., 2006).

In Hemiptera, Reduviidae, Polytecnidae and Cimicidae families present species that developed hematophagy. The majority of the hemipteran species are phytophagous such as aphids and cicadas, but the order also gathers other feeding behaviors such as predation of small vertebrates and insects (Beard, 2004). The adaptation to digesting vertebrate blood by hemipteran species required different strategies when compared to most studied hematophagous insects (mainly Diptera). Hemipterans perform digestion in an acid gut by lysosomal-like cathepsins instead of the most common trypsin-like enzymes secreted in an alkaline gut (Garcia and Garcia, 1977; Terra and Ferreira, 2005). It has been postulated that the recruitment of lysosomal-like cathepsins in digestion in Hemipterans was a secondary adaptation for protein digestion, after the loss of the peritrophic membrane, counter-current fluxes and serine endopeptidases that probably occurred in a sap-sucking ancestor (Houseman and Downe, 1983; Terra, 2001).

In Reduviidae, the Triatominae subfamily also known as “kissing bugs” is a hematophagous group presenting a widespread distribution in the American continent through different ecosystems from Argentina to the USA. These hematophagous species are potential vectors of the Chagas disease parasite *Trypanosoma cruzi* (Beard, 2004). This neglected disease is historically associated with countryside areas of Latin America and currently affects up to 7 million people. The epidemiology of Chagas disease has recently changed, with the increase of its distribution to include several urban habitats (WHO, 2017). *Rhodnius prolixus* Stål (1859) is one of the main vectors of *Trypanosoma cruzi* in South/Central America and one of the most used triatomine laboratory models to study insect physiology and interaction with *Trypanosoma* spp. (Wigglesworth, 1972).

Haemoglobin and albumin are major components of vertebrate blood, and because of that the study of digestion in hematophagous insects has primarily focused in protein degradation and absortion. In mosquitoes (Diptera), the main proteases involved in blood digestion are serine proteases as trypsin and chymotrypsin, plus carboxypeptidases and aminopeptidases (Clements, 2000). In Triatomines like *Rhodnius prolixus*, belonging to the order Hemiptera, serine proteases (e.g., trypsin and chymotrypsin) activities are not major midgut activities. However, digestive Cathepsin D-like aspartic-proteinases, Cathepsin-L like cysteine-proteinases, plus aminopeptidases and carboxypeptidases have been described and are involved in digestion of blood proteins and interaction with pathogens as *T. cruzi* (Garcia et al., 2010). Recent transcriptome analysis of *R. prolixus* digestive tract described the main groups of proteins involved in the main functions in this tissue (e.g., digestion, immune system), indicating possible gene expansion in cysteine and aspartic proteinases (Ribeiro et al., 2014). Besides that, the genome of *R. prolixus* was recently sequenced (Mesquita et al., 2015) with the mapping of several proteinase families. Despite this evidence, there are still no detailed genomic studies supporting the hypothesis of proteinase family amplification in this hematophagous insect.

In this study, we extended previous reports, screening the *R. prolixus* genome to identify peptidases from each family described in the MEROPS database (Rawlings et al., 2014). We performed further analyses to determine and characterize genetic expansion of peptidase families that may be linked to adaptations for hematophagy in triatomines. We detected two main mechanisms of expansion in protease gene families: putative gene duplication in ancestral gene families and horizontal gene transfer from microorganisms. These two mechanisms were probably necessary during evolution in triatomines as they provided a more diverse array of digestive proteases for the degradation of the high protein amounts ingested during hematophagy. We further explored the diversification in *R. prolixus* peptidase gene families using family A1 as an example. Comparison of *R. prolixus* sequences with canonical A1 peptidases from mammals (pepsin, cathepsin D, and renin) corroborated the establishment along the evolution of a very diverse array of enzymes.

## Materials and methods

A summary of our strategy and findings is presented in Supplementary Figure 1.

### Genome screening and bioinformatics analysis

The peptidase families (defined as described in the MEROPS database) in *Rhodnius prolixus* genome were characterized using FAT software (Seabra-Junior et al., 2011), which integrates HMMER (http://hmmer.janelia.org/) and BLAST+ tools (Camacho et al., 2009) to filter the initial dataset and perform automatic annotation. The filter step used the HMG-box conserved domain (Pfam code varied between families) to identify and extract only proteins containing such a domain in the *R. prolixus* dataset (VectorBase, http://www.vectorbase.org, *Rhodnius prolixus* CDC, RproC1.2). The annotation step compared the filtered proteins for similarity with proteins and conserved domains databases using respectively (a) BLAST with *nr* and *Swiss-prot uniprot* databases and (b) HMMSCAN software with *Pfam* database (Finn et al., 2010). All results were manually inspected.

We performed comparisons of protease gene frequencies between species following three different strategies:

1. Comparison of gene frequencies between Hematophagous (7 species) and Non-hematophagous arthropods (12 species). The non-parametrical statistical Mann–Whitney U (Milton, 2012) test was used to verify if the number of genes of each peptidase family varied among groups related to the ability to blood feed (i.e., species that feed on blood vs. other feeding behaviors without blood) and was performed by SPSS Statistics 23 (IBM, New York, United States). Box plot graphs were created for the peptidase families with significant differences.
2. Comparison of gene frequencies between species from different taxonomical groups, namely Hemiptera (2 species), Diptera (6 species), Hymenoptera (6 species), Mixed insect orders (Coleoptera, Lepidoptera, and Phthiraptera, three species) and Non-insects (Acari and Crustacea, two species). The non-parametrical statistical Kruskal–Wallis (Breslow, 1970) test was used to verify if the number of genes of each peptidase family varied among Classes (i.e., Diptera, Hemiptera, Hymenoptera, other insects, and other arthropods) and was performed by SPSS Statistics 23 (IBM, New York, United States). It. Box plot graphs were created for the peptidase families with significant differences.
3. Comparison of *R. prolixus* protease gene frequencies with the Percentile 90 from other 18 arthropod genomes. We compared the results of the *R. prolixus* screening to the peptidase information of 18 arthropod species (i.e., *Ixodes scapularis, Daphnia pulex, Tribolium castaneum, Aedes aegypti, Anopheles gambiae, Culex quinquefasciatus, Drosophila melanogaster, Drosophila pseudoobscura, Glossina morsitans, Acyrthosiphon pisum, Apis mellifera, Acromyrmex echinatior, Camponotus floridanus, Harpegnathos saltator, Solenopsis invicta, Nasonia vitripennis, Danaus plexippus*, and *Pediculus humanus*) stored in the MEROPS database (Rawlings et al., 2014). We calculated the percentile 90 in each peptidase family using the gene numbers for these 19 genomes of arthropod species. Then we selected families where *R. prolixus* number was higher than the percentile 90 above.

We manually drew a Venn's diagram with the peptidase families distribution according to the three criteria described above (1—higher in blood feeders vs. non-blood feeders; 2—higher in Hemiptera vs. other orders; 3—higher in *R. prolixus* vs. other genomes).

We performed phylogenetic analysis in families where *R. prolixus* presented a significant number of peptidases. We aligned sequences of each selected protein family with Clustal Omega using the correspondent PFAM curation model (Sievers et al., 2011). PHYLIP package (Felsenstein, 1989) PROML was used for phylogeny calculations. The bootstrap analysis used a 100 replicates dataset created with SEQBOOT and analyzed as above, before support calculation using CONSENSE. All programs were used with standard parameters. Trees and dendrograms were created using FIGTREE (Rambaut, 2009) and edited with GIMP. For families A1 and C1 we included the peptidase information of 5 other hemipteran species (*Triatoma infestans*; *Lygus hesperus*; *Riptortus pedestris*; *Acyrthosiphon pisum*, and *Diaphorina citri*) stored in the NCBI database.

The Phobius web server (http://phobius.sbc.su.se; Käll et al., 2004) was used to identify transmembrane topology and signal peptides in *R. prolixus* peptidases.

Protein structures of A1 peptidase sequences were inferred by Protein Homology/analogY Recognition Engine V 2.0 (Phyre^2^; Kelley and Sternberg, 2009) available online (http://www.sbg.bio.ic.ac.uk/phyre2/html/page.cgi?id=index). The Phyre^2^ Investigator was used to perform numerous quality and functional analysis in the best five protein structure models of each peptidase (Kelley and Sternberg, 2009). The ProQ2 quality assessment (Ray et al., 2012) and Ramachandran plot were used to select the best model in each peptidase. The model that present lowest relative values in both parameters was chosen to collect the functional information of catalytic and substrate binding sites.

We obtained genomic maps directly from the genome browser at the Vector Base site (https://www.vectorbase.org/organisms/rhodnius-prolixus). Shannon entropy indexes were calculated for multiple protein sequence alignments using the tool present at the Protein Variability Server (at http://imed.med.ucm.es/PVS/, Garcia-Boronat et al., 2008).

We predicted putative glycosylation sites in protein sequences using the tools NetNGlyc (at http://www.cbs.dtu.dk/services/NetNGlyc/) and NetOGlyc (at http://www.cbs.dtu.dk/services/NetOGlyc/, Steentoft et al., 2013). Surface maps of protein structures were constructed and analyzed using the PyMol Molecular Graphics System, Version 1.8 Schrödinger, LLC (https://www.pymol.org/).

### DNA amplification and expression analysis

We used male adult *R. prolixus* reared artificially on defibrinated rabbit blood (Garcia et al., 1975) for preparation of all templates used in PCR and RT-PCR reactions. We submerged insects during 30 s in ethanol 70% (v/v) for surface sterilization followed by washes in sterile NaCl 0.15 M (Sant'Anna et al., 2012). After that, we dissected the insects for complete removal of the digestive tract to avoid bacterial contamination from the gut microbiome and used carcasses for genomic DNA extraction with Wizard Genomic DNA Purification Kit (Promega, USA). Genomic DNA was digested with PstI (Cat. No. R6111, Promega, WI, USA) according to manufacturer's protocol. PCR reactions included 1 μL template (200 ng DNA), 2 μL 5x Green GoTaq® reaction buffer, 0.4 μL 10 mM dNTP, 0.6 μL 50 mM MgCl_2_, 1 μL each primer at 10 mM, 1 μL GoTaq® DNA Polymerase (Cat. No. M300, Promega, WI, USA) and 13.9 μL water (Cat. No. W4502, Sigma, MO, USA).

For the RT-PCR reactions, we dissected the entire guts of insects before blood meal (starving) and 2, 5, 7, 9, 12, and 14 days after feeding. These guts included salivary glands, anterior and posterior midguts and hindgut. We used those tissues for RNA extraction, and cDNA reverse transcription using Superscript kit (Cat. No. 18080-051, Invitrogen, CA, USA). We combined all preparations at equal cDNA concentrations for the RT-PCR templates. PCR reactions included 1 μL template (10 ng cDNA), 2 μL 5x Green GoTaq® reaction buffer, 0.4 μL 10 mM dNTP, 0.6 μL 50 mM MgCl_2_, 1 μL each primer at 10 mM, 1 μL GoTaq® DNA Polymerase (Cat. No. M300, Promega, WI, USA) and 13,9 μL water (Cat. No. W4502, Sigma, MO, USA). Supplementary Table 1 describes all primers used. All primer sequences were blasted against the *R. prolixus* genome to confirm their pairing to only one gene in the geneset (https://www.vectorbase.org/blast?organism=Rhodnius%20prolixus).

PCR conditions were as follows: initial denaturation at 94°C for 2 min, followed by 40 cycles of 94°C for 15 s, 55°C for 30 s and 72°C for 1 min, and a final extension step of 72°C for 5 min. Samples were resolved on 2% (w/v) agarose stained with ethidium bromide and viewed with an ultra-violet light transilluminator.

## Results

### Overview of peptidase genes and families in *rhodnius prolixus* genome

Peptidase families are divided according to their active site group or their dependence from metal ions in nine different groups: Aspartic (A), Cysteine (C), Glutamic (G), Metallo (M), Asparagine (N), Serine (S), Threonine (T), Mixed (P), and Unknown (U) (Rawlings et al., 2014). To obtain a more complete and broad picture of protease gene groups in *Rhodnius prolixus* genome, we screened its last available version using all PFAM signatures that are associated with known protease gene families in the MEROPS database. This screening allowed us to identify 433 protease coding genes from 71 peptidase families divided among six groups (Aspartic, Cysteine, Metallo, Asparagine, Serine, and Threonine peptidases) (Supplementary Table 2).

The majority of the families were identified within the Metallo (*N* = 25) and Cysteine (*N* = 22) groups, while the groups Aspartic (*N* = 4), Threonine (3), and Asparagine (*N* = 1) represent only 11.2% of the families sampled (Supplementary Table 2). The number of peptidases found varied among families (range: 1–94 genes) with the majority of the families showing a low number of peptidases (Percentile 50 up to 3 genes; Percentile 75 up to 6 genes). Family S1, including Trypsins and Chymotrypsins, has the highest number of peptidases (*N* = 94). Family S1 has more genes than the sum of the other top five scored families: A1 (*N* = 22), C9 (*N* = 21), S33 (*N* = 21), C1 (*N* = 17), and T1 (*N* = 15) (Supplementary Table 2).

### Transcript diversity and phylogenetic analysis of A1 and C1 peptidase families

In a recent work, Ribeiro et al. (2014) observed that sequences from families A1 and C1 were the most actively transcribed peptidase genes in the midgut of *R. prolixus*. We compared the sequences in those peptidase groups from *R. prolixus* genome with sequences from other hemipteran species to verify if gene amplification might have occurred in those multigenic families in the Triatomine branch.

**Figure 1:**
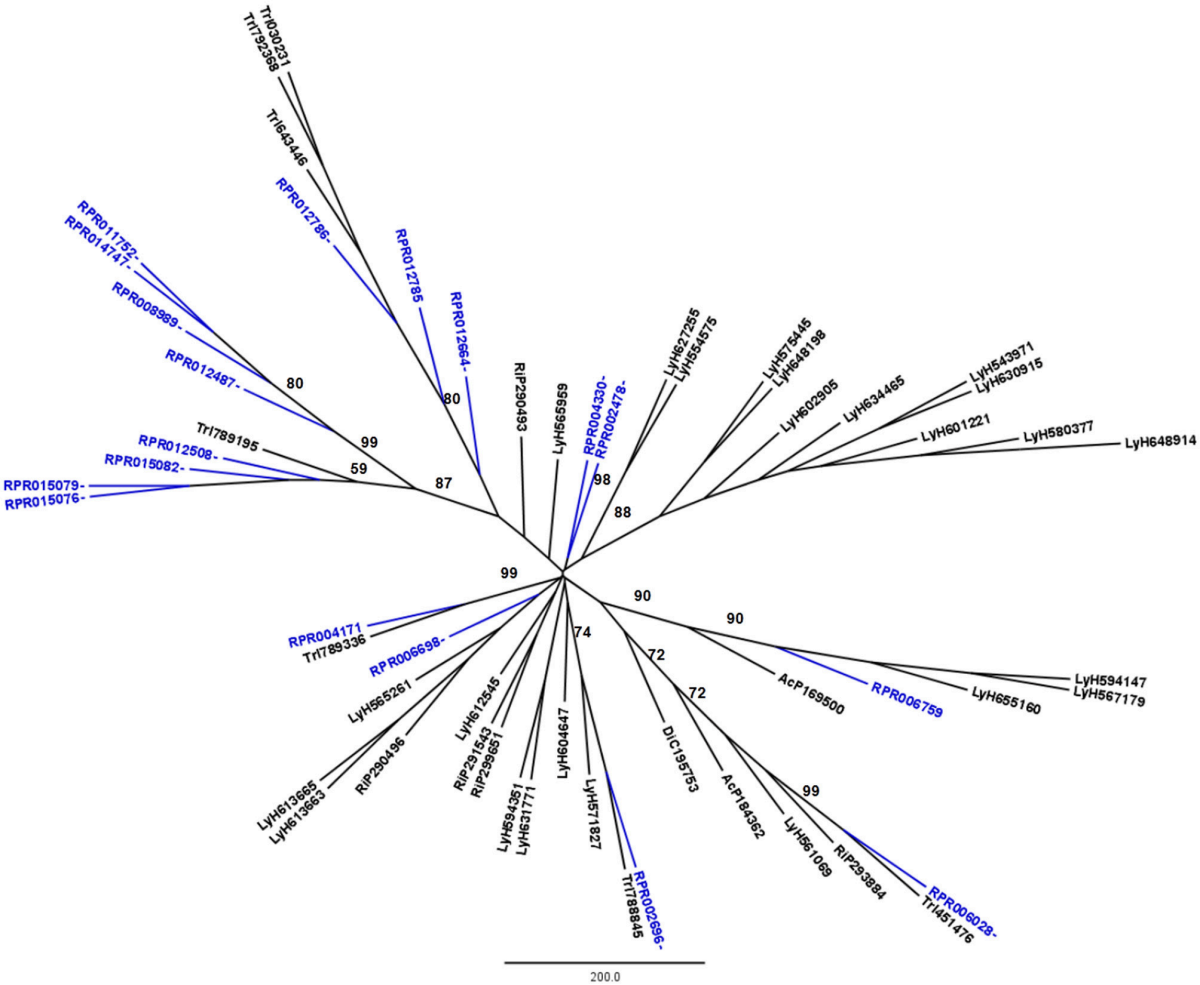
Phylogenetic trees of amino acid sequences from the peptidase family A1 (Pepsin A or Cathepsin D-like proteins) in Hemiptera. Blue, branches with *Rhodnius prolixus* peptidases (RPR); Black, branches with peptidases from other species; TrI, *Triatoma infestans* (hemathophagus Heteroptera); LyH, *Lygus hesperus* (Heteroptera); RiP, *Riptortus pedestris* (Heteroptera); AcP, *Acyrthosiphon pisum* (Aphidiformes); DiC, *Diaphorina citri* (Psylliformes). Basal bootstrap above 50 is shown in the tree.

**Figure 2:**
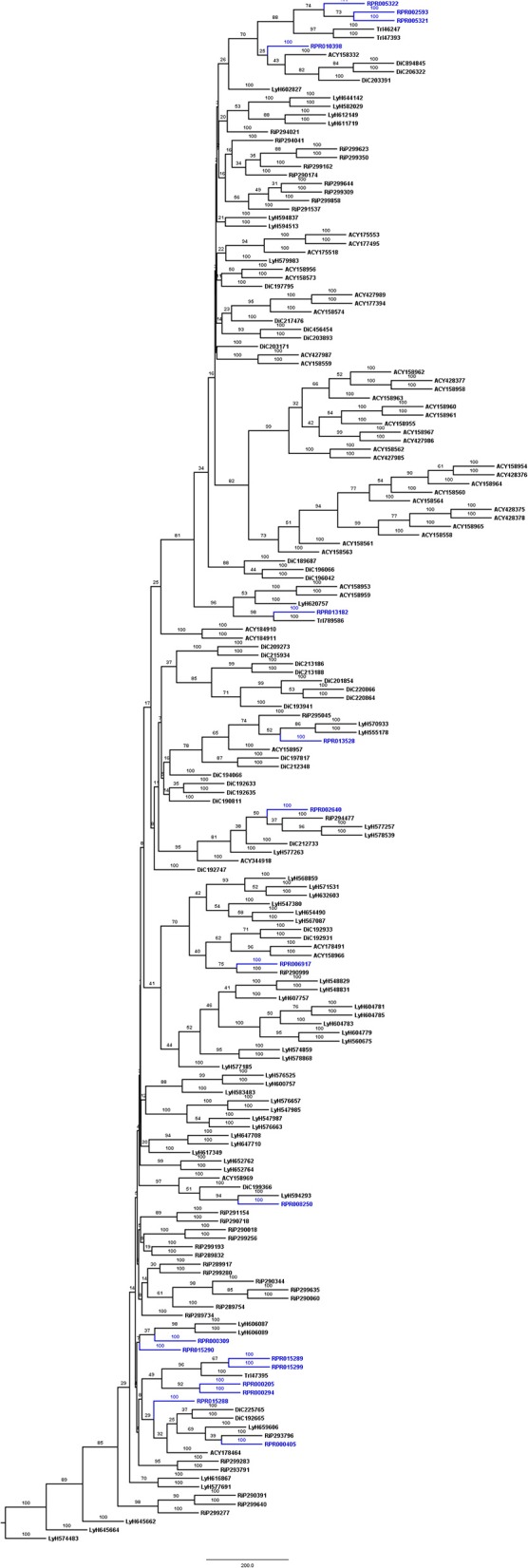
Phylogenetic trees of amino acid sequences from the peptidase family C1 (Papain) in Hemiptera. Blue, branches with peptidases (RPR); Black, branches with peptidases from other species; TrI, *Triatoma infestans* (hemathophagus Heteroptera); LyH, *Lygus hesperus* (Heteroptera); RiP, *Riptortus pedestris* (Heteroptera); AcP, *Acyrthosiphon pisum* (Aphidiformes); DiC, *Diaphorina citri* (Psylliformes).

*R. prolixus* transcriptome contains 18 and 17 sequences belonging to families A1 and C1, respectively (Supplementary Table 2). Other hemipterans with available transcriptome projects in GenBank are *Triatoma infestans* (kissing bug, hemathophagus Heteroptera), *Lygus hesperus* (western tarnished plant bug, phytophagous Heteroptera), *Riptortus pedestris* (bean bug, phytophagous Heteroptera), *Acyrthosiphon pisum* (pea aphid, phytophagous Aphidiformes) and *Diaphorina citri* (asian citrus psyllid, phytophagous Psylliformes). The transcriptomes of *Tr. infestans, Ly. hesperus, Ri. pedestris, Ac. pisum*, and *Di. citri* contain a variable number of entries from those peptidase families, with 7, 5, 24, 2, and one sequences in family A1 (Figure 1) and 4, 31, 52, 43, and 30 sequences in family C1, respectively (Figure 2).

Phylogenetic analysis showed that 11 genes from family A1 in the *R. prolixus* genome (RPR015076, RPR015079, RPR015082, RPR012508, RPR012487, RPR008989, RPR014747, RPR011752, RPR012786, RPR012785, and RPR012664, corresponding to 61% of this family) grouped in the same clade with *Tr. infestans* sequences (Figure 1). Two other genes group together at the base of the phylogenetic tree (RPR004330 and RPR002478) and the remaining entries locate in different branches, with occasional grouping with other *Tr. infestans* sequences.

*Rhodnius prolixus* peptidases from family C1 do not show the same phylogenetic distribution as A1 peptidases when compared to other sequences from hemipterans (Figure 2). Only two remote small branches showed grouping of *R. prolixus* sequences, one with three sequences (RPR005322, RPR002593, and RPR005321, 17% of the family) and another with four entries (RPR015289, RPR015299, RPR000205, and RPR000294, 24% of the family). These results suggest that the number of gene duplications during the diversification of the Triatominae branch was higher in the peptidase family A1 than in family C1.

After those phylogenetic comparisons, we analyzed the sequences from A1 and C1 peptidase families in *R. prolixus* genome to predict the cellular localization of their gene products (Supplementary Table 3). Using two different algorithms (Signal-IP and Phobius), we observed that the majority of the proteins in those two peptidase families are probably non-cytoplasmic. From a total of 18 and 17 members in each family, Phobius predicted a non-cytoplasmic location for 17 and 16 proteins of families A1 and C1, respectively. From those proteins, Signal-IP detected a putative signal peptide in 13 proteins in both families (Supplementary Table 3). Only one protein from each family was assigned as cytoplasmic (Supplementary Table 3). These results suggest that the majority of proteins from families A1 and C1 coded in *R. prolixus* genome is secreted.

### Comparative analysis of peptidase families in arthropod genomes

To have a broader understanding of the evolution of peptidase gene families in our model, we compared the number of peptidase hits in *R. prolixus* genome with the information from other arthropod genomes available in the literature. We also searched for proteinase families with an increased number of hits in Hemipterans or hematophagous species. These amplified families might be involved in the adaptation for hematophagy in Hemiptera, which would include the triatomine group.

Initially, we compared *R. prolixus* hits with the genomes available in the MEROPS database, as these sequences have been subjected to further analysis and curation of the corresponding gene data sets. Using that criterion, we found 18 available arthropod genomes that are described in Supplementary Table 4.

From the selected arthropod species (Supplementary Table 4), 16 are insects, one is a crustacean, and one is a chelicerate. Among the insect species, seven orders are represented (Hemiptera, Coleoptera, Diptera, Hymenoptera, Lepidoptera, Phthiraptera), with a predominance of dipterans and hymenopterans. In respect to feeding habits, there are phytophagous, detritivores, omnivorous, fungivores, carnivores, and hematophagous species, with six different blood feeders (kissing bug, mosquitoes, tick, and louse). Those genomes contain a variable number of peptidase families (ranging from 53 to 62, median 58.5), with a predominance of cysteine and metallopeptidase groups (medians 19 and 21, respectively) (Supplementary Table 5). However, regarding the number of genes serine peptidases (median 244) predominate over metallo (median 136) and cysteine peptidases (median 86). The number of genes in Aspartic (median 7.5), Asparagin (0), and Threonin (23.5) peptidase families are smaller than in the classes above (Supplementary Table 5).

**Figure 3:**
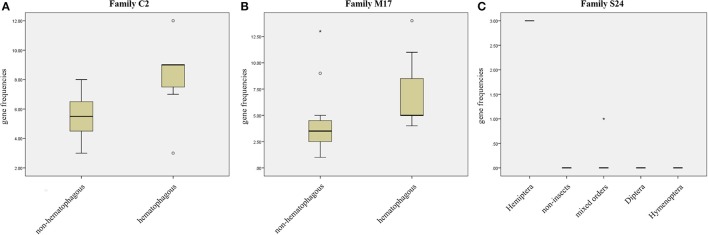
Gene frequency comparisons in three peptidase families. **(A)** Comparison between hematophagous and non-hematophagous species of peptidase gene frequencies in family C2. **(B)** Comparison between hematophagous and non-hematophagous species of peptidase gene frequencies in family M17. **(C)** Peptidase frequency distribution in five groups defined by the taxonomic order in peptidase family S24. Boxes are frequencies between 25 and 75%, with medians; error bars represent non-outlier ranges; ° are outliers; ^*^ are extremes.

We compared protease gene frequencies between species using different strategies (see Material and Methods for details). Comparison of gene frequencies between Hematophagous (7 species) and Non-hematophagous arthropods (12 species) revealed five protease families with significant differences in gene frequencies between these two groups (Mann-Whitney test, *p* < 0.05). Higher gene frequencies in the hematophagous group were observed in families C2, M17, and M41 and lower gene frequencies in families A28 and C64/C85 (Supplementary Table 6). Typical results of these comparisons are presented in Figures 3A,B.

Arranging species in different taxonomical groups (Hemiptera, Diptera, Hymenoptera, Mixed insect orders, and Non-insects) allowed us to identify 20 peptidase families with significant differentiation among these five sets (Kluskal-Wallis test, *p* < 0.05; A22, C1, C13, C40, C69, C86, M12B, M17, M19, M38, M79, M87, S1, S10, S24, S28, S33, S54, S60, and S72), including three highly significant (*p* < 0.01; C13, M19, and M79; Supplementary Table 7 and Figure 3C).After comparing *R. prolixus* protease gene frequencies with the Percentile 90 from the other 18 arthropod genomes, we observed seven families where *R. prolixus* gene frequencies are higher than in most of other species (greater than Percentile 90): A1, C2, M17, M74, N6, S24, and S29 (Supplementary Figure 2).

**Figure 4:**
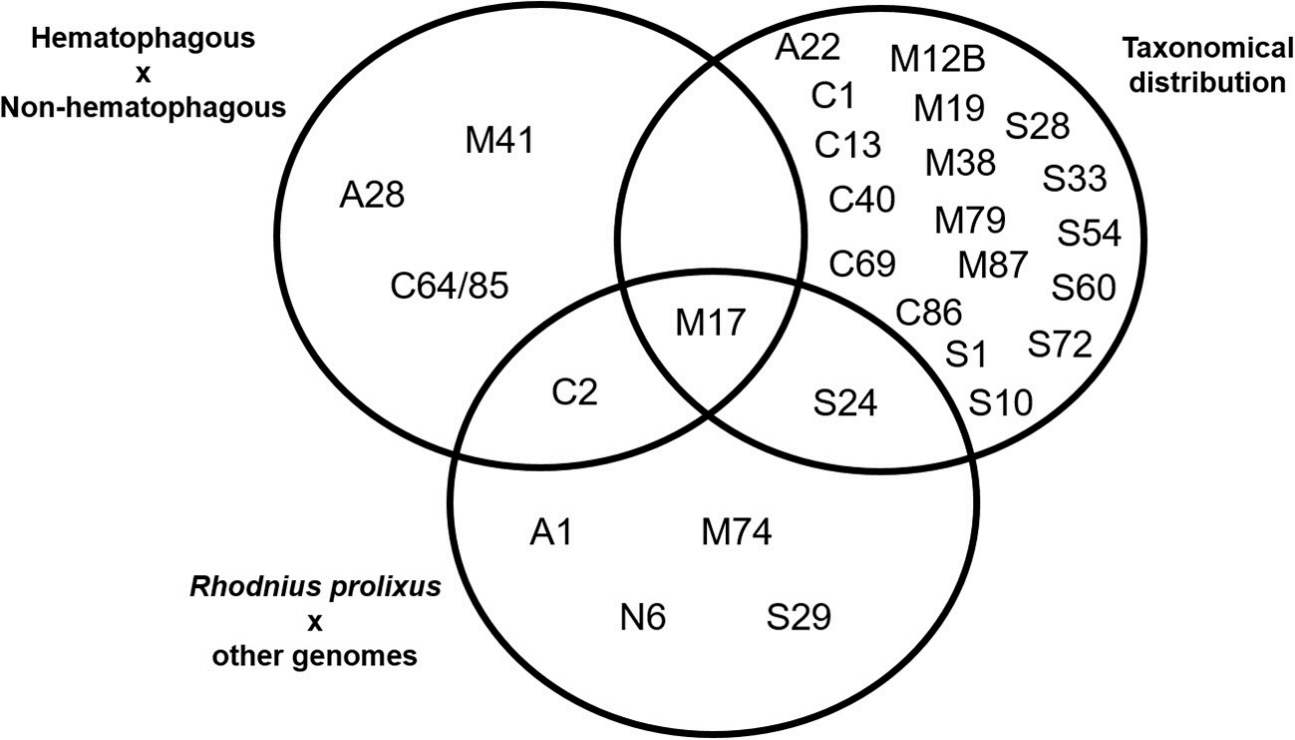
Venn's diagram of the clustering of MEROPS families after statistical analysis of peptidase gene frequencies in 19 arthropod genomes, including *Rhodnius prolixus*. Gene families with significantly higher/lower frequencies according to each criteria highlighted were included in each set. See Material and Methods for details.

A comparison between the results obtained using these three strategies is presented in Figure 4. We observed that some families were assigned as significantly changed in *R. prolixus* by more than one criterion (C2, M17, and S24) but, at the same time, the majority of families which were assigned as changed by some criteria were highlighted by only one strategy (90%). It is noteworthy that the presence of hematophagy and taxonomic distribution criteria have highlighted very different sets of peptidase families. In fact, taxonomic group history seems to have influenced peptidase diversity (20 families) more than blood feeding (5 families).

In respect to *R. prolixus*, we can consider that the amplification observed in some peptidase families seem to be directly related to the blood feeding habit (C2 and M17). Besides that, amplification in families M17 and S24 might be associated with its Hemipteran heritage. The amplification observed in other families (A1, M74, N6, and S29) might be related to the specific evolutionary history of *R. prolixus* or the triatomine group.

Further analyzing the gene counts of *R. prolixus* peptidase families with frequencies greater than the percentile 90 from the other 18 arthropod genomes (Figure 4 and Supplementary Table 5), we observed two different categories. (1) Common peptidases in arthropods that may be expanded in *R. prolixus* genome. Families A1, C2, and M17 belong to this category, with Percentiles 90 of 18.2, 9 and 11.4, respectively. (2) Sporadic/absent peptidases in insects that may have microorganism origin (i.e., M74, N6, S24, and S29). All these families have very low gene counts in general, with M74 and S29 showing Percentiles 90 equal to zero, N6 with Percentil 90 equal to 0.2 and Family S24 with Percentil 90 equal to 1.4. Using these criteria, we decided to perform phylogenetic and expression analysis of these gene families.

### Gene number and phylogenetic analysis of A1 peptidase family

We initially found 23 hits belonging to the peptidase family A1 (pepsin A, Cathepsin D-like proteins) in *R. prolixus* genome (Supplementary Table 5). Using the information available in the gut transcriptome (Ribeiro et al., 2014), we observed that ten sequences previously assembled as different genes code for only five transcripts. We joined those sequences and interpreted them as single gene counts (Supplementary Table 1). With this correction, we conclude that *R. prolixus* contains 19 genes belonging to the A1 peptidase family. We compared these sequences to the peptidases found in the other 18 arthropod genomes (Supplementary Figure 3A). From the 18 *R. prolixus* sequences analyzed (RPR010954 is truncated), 15 (83%) group in the same branch (RPR011752, RPR14747, RPR008989, RPR012487, RPR015082, RPR015076, RPR015079, RPR012508, RPR012664, RPR012785, RPR012786, RPR002478, RPR004330, RPR006698, RPR004171) and two other sequences (RPR006028 and RPR006759) group with *Acyrthosiphon pisum* or *Pediculus humanus* genes. RPR002696 did not group with any other sequence. In this respect, the comparison of genomic information confirms the previous observation that the majority of A1 peptidases in the genome of *R. prolixus* might be products of recent duplications.

For the sake of comparison, we did the same analysis with sequences from C1 peptidase family (papain, Cathepsin-L like proteins; Supplementary Figure 3B). *R. prolixus* contains 17 genes belonging to this family, and phylogenetic analysis (Supplementary Figure 3B) revealed that only four sequences (24% of the family) group in the same branch (RPR002593, RPR005321, RPR005322, and RPR010398). RPR015299 and RPR015289 clustered in a small branch, as well as RPR000294 and RPR000205. The other 9 *R. prolixus* sequences from this family are dispersed along the phylogenetic tree. These observations agree with the previous analysis of transcriptomic data and suggest that most duplications in the C1 family are older than the divergence of the Triatominae family.

### Gene number and phylogenetic analysis of C2 peptidase family

**Figure 5:**
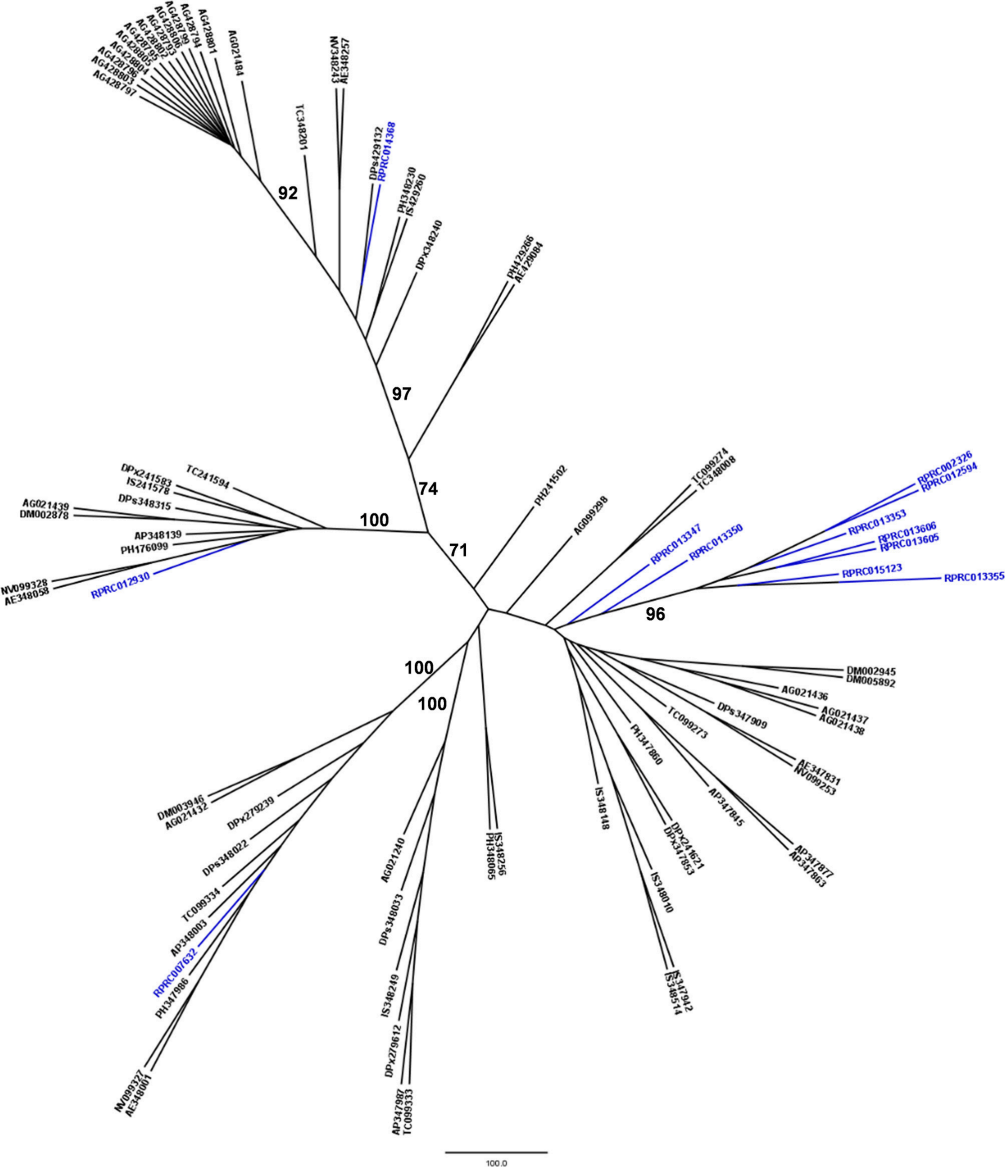
Phylogenetic tree of amino acid sequences from peptidase family C2 (Calpains) common in insect genomes which contain high gene copy numbers in the *R. prolixus* genome. Tree gather sequences from 19 arthropod species. Blue, branches with *Rhodnius prolixus* peptidases; Black, branches with peptidases from 18 arthropod species; ACY, *Acyrthosiphon pisum*; IXO, *Ixodes scapularis*; DAP, *Daphnia pulex*; TRI, *Tribolium castaneum*; AED, *Aedes aegypti*; ANO, *Anopheles gambiae*; CUL, *Culex quinquefasciatus*; DRm, *Drosophila melanogaster*; DRp, *Drosophila pseudoobscura*; GLO, *Glossina morsitans*; API, *Apis mellifera*; ACR, *Acromyrmex echinatior*; CAM, *Camponotus floridanus*; HAR, *Harpegnathos saltator*; SOL, *Solenopsis invicta*; NAS, *Nasonia vitripennis*; DAN, *Danaus plexippus;* PED, *Pediculus humanus*. See Material and Methods for details.

Another peptidase family with a high gene count in the *R. prolixus* genome is the family C2 (calpains). *R. prolixus* contains 12 genes coding for calpains, and phylogenetic comparison with sequences from other arthropod genomes (Figure 5) reveals that 9 of them (75%) group in the same branch (RPRC013347, RPRC013350, RPRC002326, RPRC012594, RPRC013353, RPRC013606, RPRC013605, RPRC015123, and RPRC013355). The other three sequences (RPRC007632, RPRC012930, and RPRC014368) are dispersed along the tree without a clear pattern.

We performed a similar analysis comparing *R. prolixus* C2 sequences to transcripts from several hemipteran species (Supplementary Figure 4). In this case, seven *R. prolixus* sequences (58% of the family; RPRC002326, RPRC012594, RPRC013353, RPRC013606, RPRC013605, RPRC015123, and RPRC013355) group together. Other two *R. prolixus* sequences (RPRC013347 and RPRC013350) group together independently of the rest of the tree. RPR014368 and RPR012930 group with *Triatoma infestans* hits and RPR007632 group with *Lygus hesperus* proteins (Supplementary Figure 4). These analyses altogether suggest that at least seven C2 *R. prolixus* sequences might be products of recent gene duplications after the diversification of the main Hemipteran suborders.

The prediction of transmembrane topology and presence of signal peptide for members of family C2 in the *R. prolixus* genome was impaired due to the truncation of the amino terminal region of six predicted genes in this protein family (Supplementary Table 8). Despite that, no signal peptide was predicted for any member of this family. The only sequence with a topology marker is RPRC007632, showing a putative transmembrane region.

### Gene number and phylogenetic analysis of M17 peptidase family

Family M17 (Leucine aminopeptidases) is another peptidase family with high gene counts in the *R. prolixus* genome. We initially found 14 coding sequences containing the M17_peptidase PFAM domain. However, transcriptome analysis revealed that two of them (RPRC014323 and RPRC10866) code for the same transcript, so they were merged in one gene (Supplementary Table 9). In this respect, we can conclude that *R. prolixus* genome code for at least 13 peptidases of family M17.

**Figure 6:**
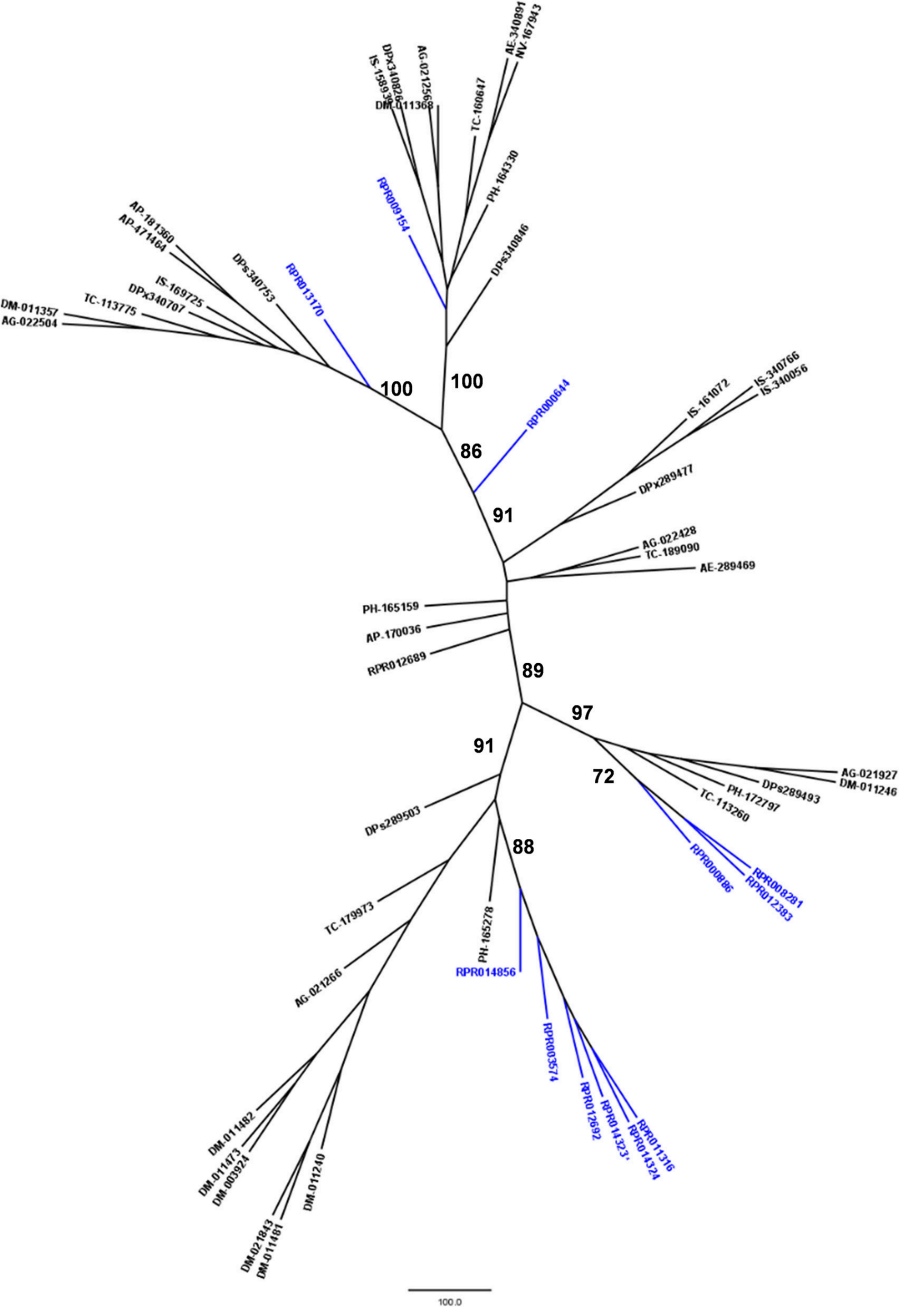
Phylogenetic tree of amino acid sequences from peptidase family M17 (Leucine Aminopeptidases) common in insect genomes which contain high gene copy numbers in the *Rhodnius prolixus* genome. Trees gather sequences from 19 arthropod species. Blue, branches with *R. prolixus* peptidases; Black, branches with peptidases from 18 arthropod species; ACY, *Acyrthosiphon pisum*; IXO, *Ixodes scapularis*; DAP, *Daphnia pulex*; TRI, *Tribolium castaneum*; AED, *Aedes aegypti*; ANO, *Anopheles gambiae*; CUL, *Culex quinquefasciatus*; DRm, *Drosophila melanogaster*; DRp, *Drosophila pseudoobscura*; GLO, *Glossina morsitans*; API, *Apis mellifera*; ACR, *Acromyrmex echinatior*; CAM, *Camponotus floridanus*; HAR, *Harpegnathos saltator*; SOL, *Solenopsis invicta*; NAS, *Nasonia vitripennis*; DAN, *Danaus plexippus*; PED, *Pediculus humanus*. See Material and Methods for details.

Phylogenetic comparison with protease genes of other arthropod genomes (Figure 6) revealed that six M17 *R. prolixus* genes (RPRC014856, RPRC003574, RPRC012692, RPRC014323, RPRC014324, and RPRC011316) cluster in one single branch. Other three sequences of this family (RPRC000886, RPRC012383, and RPRC008281) group with sequences from other insects in another separate branch. RPRC009154 and RPRC013170 locate with other sequences in two other different groups, and RPRC000644 do not group with any sequence. This distribution suggests at least two distinct gene duplication events in the M17 family during the evolution of the Triatominae group.

Nevertheless, phylogenetic comparison with sequences retrieved from transcriptomes of other hemipteran species revealed that all M17 *R. prolixus* sequences group separately with the sequences of other hemipterans (Supplementary Figure 5). As we did not observe *R. prolixus* M17 sequences clustered in any branch, it seems that recent duplications (after the divergence of the Triatominae group) have not occurred in this gene family.

Sequence analysis of M17 *R. prolixus* sequences using the tools Signal-IP and Phobius revealed that no sequence from this protein family bear a putative signal peptide (Supplementary Table 8), but two of them (RPRC003574 and RPRC012692) present putative transmembrane regions. Considering that most of the translated sequences have an initial Methionine residue (9 from 13), being probably complete, it seems that *R. prolixus* M17 family is constituted majorly by non-secreted proteins. Particular care should be taken with the four sequences which are truncated (RPRC000644, RPRC008281, RPRC012692, and RPRC014856) because in those cases it is not possible to discard the presence of a putative signal peptide.

### Peptidase putatively acquired by horizontal gene transfer from microorganisms (families M74, S24, and S29)

Among the protease families with high gene counts in *R. prolixus* genome when compared to the other 19 arthropod genomes that are present in the MEROPS database (Figure 4), there are families M74, S24, S29, and N6. These families are characterized by having low gene frequencies (1–3) in *R. prolixus*. However, because they are absent in most of the other arthropod genomes analyzed, *R. prolixus* frequencies are higher than the percentile 90 that was calculated to each of these protease families.

Protease family M74 (murein endopeptidases) is represented in *R. prolixus* genome data by the gene RPRC003168. No hits for this family were found in the other 18 arthopod genomes studied. Similarly, we found two genes belonging to Family S29 (Hepacivirin) in *R. prolixus* genome data (RPRC004810 and RPRC013821), and no S29 hits were found in the other genomes analyzed.

*Rhodnius. prolixus* genome sequences also contain three proteases (RPRC002798, RPRC005865, and RPRC010630) of family S24 (repressor LexA). Family S24 proteins were also found in the genomes of *Acyrthosiphon pisum* (3 hits) and *Apis mellifera* (1 hit), with a percentile 90 for this family of 1.4 (Supplementary Table 4).

Family N6 (autoprocessing endopeptidases like YscU) has one gene (RPRC014779) in *R. prolixus* genome data. Only one hit was found in the other 18 genomes analyzed, in *Daphnia pulia*. The calculated percentile 90 for this family was 0.2 (Supplementary Table 4).

Similarity searches using the blastp tool and *R. prolixus* sequences of these protease families as queries resulted only in sequences with microorganism origin (bacteria and viruses, data not shown). This fact raised the possibility that these sequences are the product of contaminations of the genomic DNA used to produce the *R. prolixus* genome data. We decided to look at the neighbor genes of these protease genes in their corresponding contigs to explore this hypothesis. In this respect, the presence of neighbors with putative bacterial origin would reinforce the contamination hypothesis, and neighbors with arthropod or animal homologs would favor the idea that these proteases are coded by *R. prolixus* actual genes.

The results are presented in Supplementary Figure 6 and Supplementary Table 10. In the case of family M74, the gene RPRC003168 has no neighbors in its contig, so this strategy was not applicable. The member of family N6, Gene RPRC014779, has as neighbor RPRC014780, a gene that has similarity only with bacterial sequences, being a putative stress protein from enterobacteria. This result suggests that RPRC014779 is a contamination and that N6 proteases do not belong to the genomic repertoire of *R. prolixus* peptidases. Contrarily to that, most sequences from families S24 (RPRC002798 and RPRC005865) and S29 (RPRC004810 and RPRC013821) showed neighbors who are similar to animal/arthropod genes, reinforcing that their sequencing is not from contaminating material and that they are probably present in *R. prolixus* genomic DNA. One exception is gene RPRC010630 from family S24, which presented neighbors similar to bacterial proteins, an uncharacterized DUF2800 domain-containing protein (RPRC010628) and an ATPase (RPRC010632). It is noteworthy that both genes above contain introns, which is not coherent with their suggested bacterial origin.

**Figure 7:**
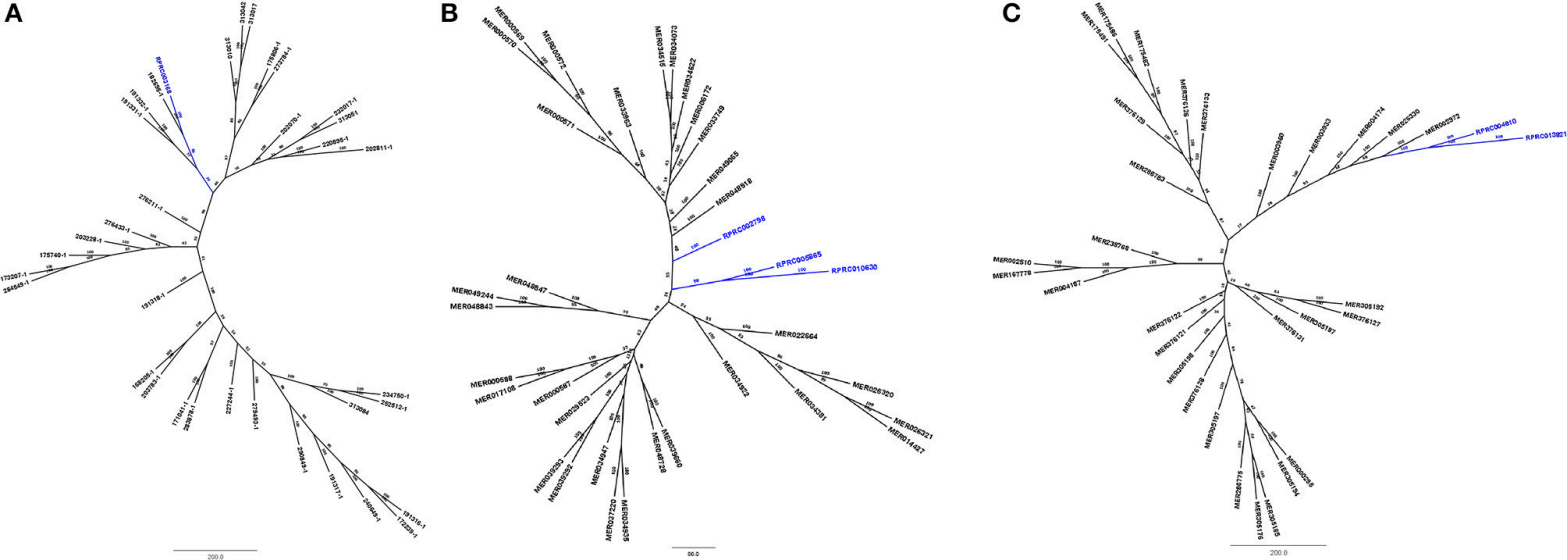
Phylogenetic tree of amino acid sequences from peptidase families typical for microorganisms which present genes in *Rhodnius prolixus* genome. **(A)** Family M74 (Murein endopeptidase); **(B)** S24 (repressor LexA); **(C)** S29 (Hepacivirin).

Phylogenetic analysis of protein sequences from family M74 (Figure 7A) shows that the sequence coded by the gene RPRC003168 groups in a monophyletic clade with sequences MER182695 (from *Dickeya dedantii*), MER191332 (from *Yersinia rohdei*) and MER191331 (from *Yersinia ruckeri*), supporting its homology to bacterial counterparts. Analysis of S24 peptidases (Figure 7B) showed that genes RPRC005865 and RPRC010630 group together in a single clade, and RPRC002798 remains alone in a single branch. None of *R. prolixus* S24 peptidase sequences grouped with other proteins of the family. Comparison of *R. prolixus* genes of family S29 with other proteases of this family (Figure 7C) revealed that RPRC004810 and RPRC013821 group together at the end of a branch formed by sequences from hepatitis C virus type 4a (MER003960), hepatitis C virus type 6a (MER003933), hepatitis C virus type 3g (MER004174), hepatitis virus type 3g (MER026330) and hepatitis C virus type 3b (MER002972). Finally, sequence RPRC014779 from family N6 grouped with sequences from *Pelotomaculum thermopropionicum* (326890A1), *Serratia plymuthica* (326898A1), *Thermoanaerobacter wiegelii* (273717), *Paenibacillus terrae* (326897A1), *Bacillus licheniformis* (326887A1), *Bacillus amyloliquefaciens* (165824), *Bacillus subtilis* (326896A1), *Candidatus Arthromitus* (165925), *Candidatus Arthromitus* (166080) (Supplementary Figure 7). These results all together suggest that protease sequences from families M74, S24, and S29 are indeed part of the genome of *R. prolixus* and that N6 sequences were obtained from contaminations in the samples used in the genome project.

### Assessment of gene structure and expression analysis of selected peptidase families

We decided to confirm the presence in the genome of *R. prolixus* of protease genes belonging to protease families that showed signs of gene amplification during the evolution of the triatomine branch. Additionally, we tested by RT-PCR if these genes are actually expressed in the gut using as a template a pool of RNAs extracted from entire guts (including salivary glands, anterior midgut, posterior midgut, hindgut) collected throughout the digestion of 5th instar larvae (2, 5, 7, 9, 12, and 14 days after blood feeding). Namely, those genes correspond to the sequences from families A1, C2, M17, M74, S24, and S29, discussed above.

Amplification of fragments of peptidase coding genes corresponding to family A1 from genomic DNA resulted in amplicons with the expected size, confirming their presence and partial structure (Supplementary Table 1 and Supplementary Figure 8A left column). All A1 protease genes are also expressed in *R. prolixus* gut, as fragments of the expected size, were obtained from cDNA prepared from guts at different time points after blood feeding (Supplementary Figure 8A right column). Similarly, fragments of an expected size corresponding to all the genes coding proteases from family C2 were amplified from genomic DNA (Supplementary Table 1 and Supplementary Figure 8B left column). Most of the C2 genes are expressed in the gut, with the sole exception of RPRC013353 (Supplementary Figure 8B right column). The same result was obtained for M17 protease coding genes (Supplementary Table 1 and Supplementary Figure 8C left column), with the amplification of expected fragments from genomic DNA and confirmation of gut expression for all M17 genes but RPRC000644 (Supplementary Figure 8C, right column). Amplicons of an expected size corresponding to fragments of the genes that code for proteases of families S24, S29 and M74 were also amplified from genomic DNA of *R. prolixus*, confirming the insertion of these originally bacterial or viral sequences in its genome (Supplementary Table 1 and Supplementary Figure 8D, left column). Interestingly, all these genes are expressed in the gut of *R. prolixus*, as the expected fragments were also obtained from cDNA extracted from this tissue (Supplementary Figure 8D, right column).

### Sequence and structural analysis of family A1 peptidases in *rhodnius prolixus*

Family A1 correspond to the protease group with putative expansions in the *R. prolixus* genome with the highest number of gene hits observed. Briefly, families A1, C1, C2, and M17 showed 19, 17, 12, and 13 gene hits in *R. prolixus*, with 15, 4, 9, and six genes belonging to supposed recent duplications, respectively (see above). Based on that we decided to do a more detailed analysis of A1 sequences, looking for functional signatures and comparison with archetypal members of the family, namely humans Pepsin A (PGA3, Uniprot accession P0DJD8), Renin (GenBank accession AAA60363) and Cathepsin D (Uniprot accession P07339). These sequences correspond to very well characterized A1 peptidases that are respectively secreted in the gut, plasma or into the lysosome of vertebrate cells (Rawlings and Barrett, 2014). These comparisons aimed to search for structural features that might be related to the role of A1 peptidases in digestion, processing of proteins in the plasma and intracellular degradation of cellular proteins, with an especial focus in *R. prolixus* sequences.

Initially, we observed that almost all *R. prolixus* A1 sequences contain the two conserved catalytic glutamates (Supplementary Table 11, columns 1–2). The exceptions are RPRC12504 and RPCR11752. They correspond to only half of the other sequences, or just one lobe of the typical A1 structure, and contain only one of the conserved glutamates.

A1 aspartic peptidases contain six well-conserved Cysteine residues, which form three disulfide bonds inside their structure. These residues correspond to Cysteines 107/112/268/272/311/344, 117/124/283/287/325/362, and 110/117/286/290/329/366 in Pepsin A, Renin, and Cathepsin D, respectively. These six conserved Cysteines are conserved in all but three *R. prolixus* A1 peptidases (Supplementary Table 11, columns 4–5 and 7–10). The exceptions are RPRC006028, RPRC012504 and RPRC011752, with the deletion of 1, 2 and 4 Cysteines, respectively. Cathepsin D contains two additional Cysteine residues (C91 and C160), but they are not conserved either in Pepsin A or Renin, neither in none of *R. prolixus* A1 sequences (Supplementary Table 11, columns 3 and 6).

Proteases of family A1 typically contain four conserved proline residues. Two of them are conserved in Pepsin (Pro 85 and 354), Renin (Pro 95 and 372) and Cathepsin D (Pro 88 and 376), and other two are conserved in Renin (Pro 373 and 374) and Cathepsin D (Pro 377 and 378). A typical characteristic of some A1 peptidases is the “Proline loop,” consisting of residues 372–374 of Renin and 376–378 in Cathepsin D. The first Proline residue above (Pro 85, 95, and 88 in Pepsin, Renin and Cathepsin D, respectively) is conserved in all *R. prolixus* sequences but two, RPRC015076 and RPRC012504. Noteworthy, in the region corresponding to the Proline loop, most sequences of *R. prolixus* do not contain any proline residues (RPRC015709, RPRC015076, RPRC004171, RPRC012785, RPRC006759, RPRC004330, RPRC014747, RPRC002479, RPRC012504, RPRC012508, and RPRC011752), four sequences (RPRC06698, RPRC012786, RPRC015082, and RPRC012664) contain one proline residue and one sequence (RPRC006028) contains two proline residues. No A1 peptidase sequence from *R. prolixus* showed the putative complete proline loop with three residues (Supplementary Table 11).

N-linked glycosylations have been implicated in A1 peptidase structure and function, being mapped and studied in human Cathepsin D. The residues involved in these modifications are partially conserved in this protease family, corresponding to the three residues Asn134/Asn141/Thr126, Asn263/Asn260/Arg245, and Lys267/Thr264/Glu249 in Cathepsin D, Renin and Pepsin A, respectively (Supplementary Table 11). The first residue above (Asn134 in Cathepsin D) is conserved as asparagine in four *R. prolixus* sequences (RPRC006698, RPRC015079, RPRC006028, and RPRC006759) and is a lysine in one sequence (RPRC012504). The second residue (Asn141 in Cathepsin D) is conserved as asparagine in only one *R. prolixus* sequence (RPRC004171). Interestingly, this residue is an arginine in Pepsin A (Arg245), with positional identity in 12 *R. prolixus* A1 peptidases (RPRC006698, RPRC012786, RPRC015079, RPRC015082, RPRC015076, RPRC006028, RPRC012785, RPRC004330, RPRC014747, RPRC002479, RPRC012504, and RPRC012508). The third residue (Lys 267 in Cathepsin D) is a conserved lysine in three *R. prolixus* sequences (RPRC012785, RPRC014747, and RPRC012504). In this position, a threonine is observed in Renin (Thr264) and other 3 *R. prolixus* sequences (RPRC012786, RPRC004171, and RPRC006759). In Pepsin A, this position is changed to a glutamate (E249), which was observed only in one *R. prolixus* sequence (RPRC006698).

The observation that some residues involved in glycosylation in Cathepsin D were conserved in several *R. prolixus* A1 peptidases led us to check the putative glycosylation patterns of these sequences on a broader scale. The distribution of putative glycosylation sites (N- and O-linked) among a range of A1 peptidase sequences from vertebrates and its comparison with those from *R. prolixus* sequences are presented in Supplementary Figure 9. It is evident from the alignment of sequences that at least eight glycosylation sites are strongly conserved in vertebrate sequences. Surprisingly, *R. prolixus* sequences do not strictly retain any of these sites, with the only exception of a recurrent occurrence of O-glycosylation around residues 120–130 (Supplementary Table 12 and Supplementary Figure 9). Exceptions to this are RPRC006698, RPRC012786, and RPRC011752, which did not show any glycosilations in this region, and RPRC012504 and RPRC012664, which showed different numbering or absence of glycosilations due to possible truncation of their sequence (Supplementary Table 12 and Supplementary Figure 9).

Considering that most *R. prolixus* A1 peptidase sequences retain the conserved active site glutamates, being probably catalytically active, we decided to compare the residues present at known positions in the binding sites of their homologs (Supplementary Table 11). This comparison aims to understand if *R. prolixus* A1 peptidase might have an active site more similar to Pepsin, Renin or Cathepsin D, the three archetypes of A1 peptidases with well-known structures of the substrate binding sites. For example, the binding subsite S4 (numbering as Schechter and Berger, 1967) of Pepsin, Renin and Cathepsin D is formed respectively by residues of Met/Thr/Ala, a conserved Ser and Leu/Tyr/Leu. Most of *R prolixus* sequences showed an Ala residue (9 sequences), Ser (13 sequences) and Leu (11 sequences), being more similar to Cathepsin D (Supplementary Table 11).

Subsite S3 is formed by 8 residues, being composed in Pepsin, Renin and Catepsin D by Glu/Gln/Gln, Thr/Thr/Ser, Phe/Pro/Thr, Leu/Phe/Phe, conserved residues of Phe and Gly, Thr/Ala/Thr and a conserved Ser (Supplementary Table 11). In *R. prolixus* sequences, the first three positions above showed many variations, with a preponderance of Asp/Glu residues (7 sequences), Thr (9 sequences) and Pro (8 sequences). After that, we observed a highly conserved Phe (13 sequences), a less conserved Phe (9 sequences), and strongly conserved Gly (14 sequences), Thr and Ser (13 sequences both, Supplementary Table 11). In this respect, the subsite S3 in *R. prolixus* sequences bear similarities with all the three possible homologs considered.

Subsite S2 is composed of 9 residues in Pepsin/Renin/Cathepsin D, with a conserved Tyr, Gly/Ser/Gly, Thr/Thr/Ser, a conserved Gly, Thr/Ala/Thr, Thr/Ser/Val, Gln/His/Met, a conserved Met and Ile/Ala/Ile residues, respectively (Supplementary Table 11). Most *R. prolixus* sequences showed residues of Tyr (15 sequences), Gly (13 sequences), Thr (9 sequences), Gly (14 sequences), Thr (13 sequences), Ile (9 sequences), Ser (9 sequences), Leu (7 sequences) and Ile (13 sequences) at these positions. In some positions, we observed a prevalence of the similarity with Cathepsin D (the 6th position from the list above), but at others a resemblance with Pepsin (7th). Surprisingly, we did not observe conservation of the Met in the 8th position, which was strictly retained in the three homologs considered (Supplementary Table 11).

Nine residues were mapped into the S1 subsite, corresponding to conserved Val, Asp and Gly, variable Thr/Arg/His, a conserved Tyr, Leu/Phe/Phe, conserved Phe, Ile/Val/Ile and a conserved Asp in Pepsin/Renin/Cathepsin D, respectively (Supplementary Table 11). Most *R. prolixus* A1 sequences showed Ile or Val (7 sequences each), a completely conserved Asp and an almost entirely conserved Gly in the first, second, and third position respectively. The fourth position is extremely variable, with a predominance of neutral residues (Val, Ala, Ile and Pro, nine sequences). In the subsequent positions, we found a prevalence of Tyr (15 sequences), Phe (13 sequences), Phe (9 sequences), Ile (10 sequences) and Asp (15 sequences) (Supplementary Table 11).

Subsite S1′ is composed of 5 residues, including conserved Gly and Ser, variable Thr/Arg/His, conserved Tyr and a variable residue of Thr/Ala/Thr in Pepsin, Renin and Cathepsin D, respectively (Supplementary Table 11). In *R. prolixus* most sequences show in the first two positions conserved residues of Gly (14 sequences) and Ser (14 sequences). The third position is extremely variable with a predominance of neutral residues (Val, Ala, Ile and Pro, nine sequences). The remaining two locations mostly contain conserved Tyr (15 sequences) and Thr (13 sequences) residues respectively (Supplementary Table 11).

S2′ subsite is composed of a variable residue Thr/Arg/His, a conserved Tyr, and variable Gly/Ser/Gly, Tyr/Val/Tyr, Leu/Ile/Ile for Pepsin, Renin, and Cathepsin D, respectively (Supplementary Table 11). The first position is the same residue in the third position of subsite S1′ and, in *R. prolixus* sequences are highly variable. The second residue is the same as the fourth position in S1′, being a preserved Tyr in *R. prolixus* sequences (15 sequences). The third and fourth locations in *R. prolixus* A1 peptidases are represented by conserved Gly (13 sequences) and Tyr (9 sequences) residues, respectively. Moreover, the fifth position showed high diversity in *R. prolixus* sequences, with no predominance of any particular residue class (Supplementary Table 11).

**Figure 8:**
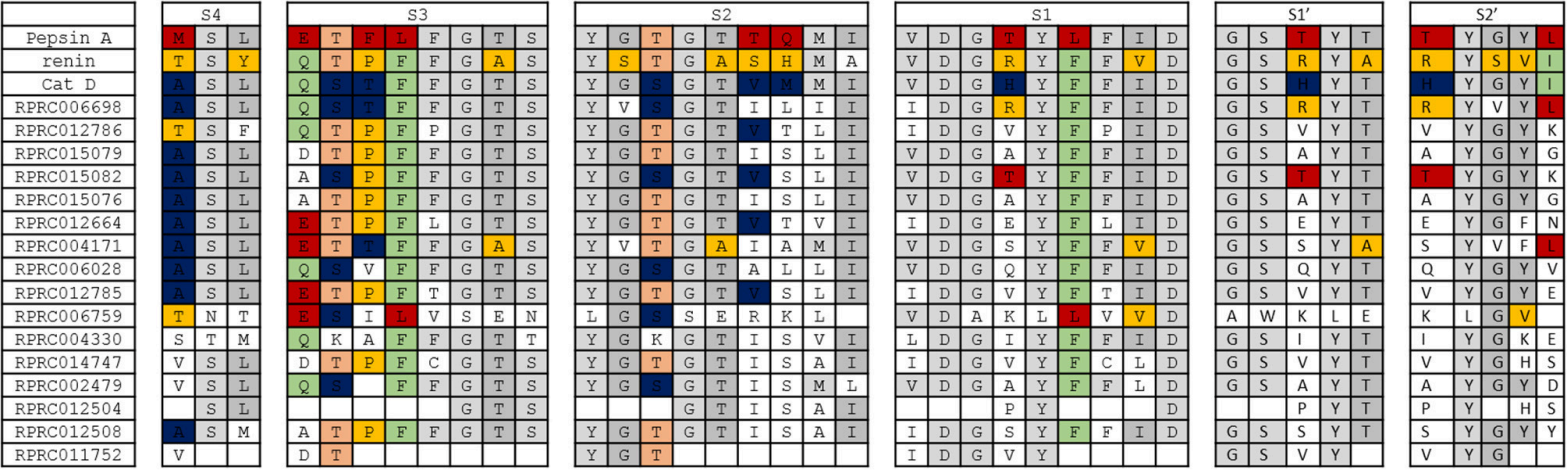
Overall view of residues involved in substrate binding across subsites S4 to S2′ in the active site of selected peptidases of family A1 and corresponding residues in *R. prolixus* homologous sequences. The background color of residues shows their presence in the archetype sequences of pepsin, renin and cathepsin D. Residues exclusive to pepsin, renin and cathepsin D are colored in red, yellow and blue, respectively. Residues common to pepsin and renin are colored in light orange, those common to renin and cathepsin D in light green and those common to pepsin and cathepsin D in light gray. Residues that are common to pepsin, renin and cathepsin D are shown in a gray tone that is even lighter than the above.

In general, we observed that the overall architecture of substrate-binding subsites of archetypal proteases of family A1 is not conserved in any of *R. prolixus* A1 peptidases (Figure 8). The only exception to this rule is subsite S4 of Cathepsin D, which is preserved in several sequences of *R. prolixus* (RPRC006698, RPRC015079, RPRC015082, RPRC015076, RPRC012664, RPRC004171, RPRC006028, and RPRC012785) (Figure 8). Despite that, it is possible to recognize in each subsite a core of residues that are common to all A1 peptidases, or at least two of the archetypal sequences considered, that are mostly preserved in *R. prolixus* sequences (Figure 8).

Due to the low conservation of substrate-binding subsites architectures in *R. prolixus* sequences, we decided to compare the variability of the aminoacid sequences of these proteins in a more general approach. Calculation of diversity indexes along protein sequences is useful to differentiate highly variable sequences from more conserved motifs (Garcia-Boronat et al., 2008). Surprisingly, highly variable residues (Shannon index above 2) are dispersed along the whole sequence of *R. prolixus* A1 peptidases (Supplementary Figure 10A). To have a wider perspective of this variation, we performed the same analysis with all 73 vertebrate A1 peptidase sequences that were present in the MEROPS database, from tunicates to humans (Supplementary Figure 10B). It is noteworthy that in vertebrate sequences, besides the first 70 residues that correspond to the signal peptide and pre-processing regions, which tend to be species-specific, highly variable positions along the catalytic domain are rare (Supplementary Figure 10B). In general, *R. prolixus* A1 peptidase aminoacid sequences showed 184 highly variable positions, and vertebrate sequences showed only 78.

**Figure 9:**
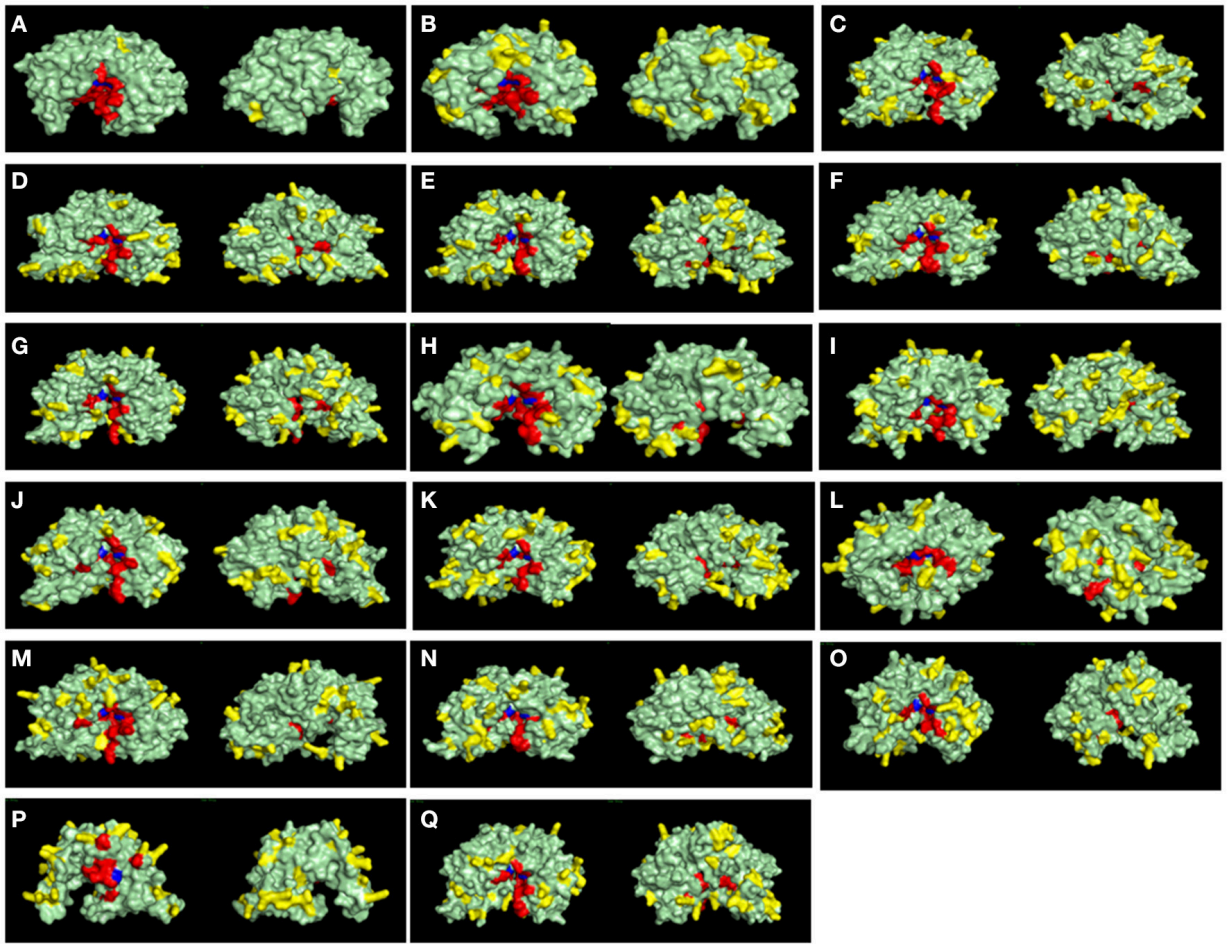
Surface view of structural models of A1 peptidase sequences from *Rhodnius prolixus* and their human counterparts. Catalytic Aspartates are depicted in blue, and other active site residues are shown in red. Basic exposed residues are shown in yellow, and the rest of protein surface is shown in light green. Left panels are the active site (front) view, and right panels are the rear view of each model. **(A)**, Pepsin A; **(B)**, Cathepsin D; **(C)**, RPRC006698; **(D)**, RPRC012786; **(E)**, RPRC015079; **(F)**, RPRC015082; **(G)**, RPRC015076; **(H)**, RPRC012664; **(I)**, RPRC004171; **(J)**, RPRC006028; **(K)**, RPRC012785; **(L)**, RPRC006759; **(M)**, RPRC004330; **(N)**, RPRC014747; **(O)**, RPRC002479; **(P)**, RPRC012504; **(Q)**, RPRC012508.

Another key feature of some A1 peptidases is the frequency of basic-charged residues on their surfaces. Specifically, the activity of some A1 peptidases in less acidic pHs has been linked to higher frequencies of surface basic residues. To assess the frequencies of basic residues on the surface of *R. prolixus* A1 peptidases, we constructed homology-based structural models for these proteins (Figure 9) and compared them with the known structures of pepsin (Figure 9A) and cathepsin D (Figure 9B). For example, pepsin A, a highly acidic peptidase (optimum pH 2), has only three exposed arginine residues and no lysins in its surface (Figure 9A and Supplementary Table 13). Meanwhile, Cathepsin D, which has a less acidic optimum pH (around 5), contains 20 lysins and eight arginine residues in its surface (Figure 9B and Supplementary Table 13). In general, *R. prolixus* A1 peptidases show a high number of exposed basic residues, ranging from 24 (RPRC012664) to 42 residues (RPRC014747), with a predominance of Lysines (18–29 residues) of Arginines (5–16 residues), a pattern that is similar to the observed in Cathepsin D (Figure 9B and Supplementary Table 13).

## Discussion

### Evolution of peptidase families in insect genomes

The number and diversity of peptidases found in *R. prolixus* (i.e., 433 protease coding genes from 71 peptidase families, item 1 of Results section) are within normal values found in arthropods (see item 3.3 of Results section). These proteins are essential for several functions in animal metabolism (e.g., digestion, blood-clotting, immune system), which explain the high diversity of this molecule type across different arthropods (Khan and James, 1998; Terra and Ferreira, 2005; Wu et al., 2009). Only a fraction of these protein coding genes would be involved in the adaptation of triatomine to blood feeding behavior, supporting blood protein digestion among other physiological functions. This food source might present several challenges such as pathogens, inhibitors and defense molecules which may interact negatively with the insect midgut (Cederlund et al., 2011; Gottdenker et al., 2012). In fact, when we compare the frequencies of *R. prolixus* peptidase families with the other arthropods genomes considered in this work, only eight families showed higher gene frequencies, suggesting that, during hematophagy evolution in this group, gene duplication and recruitment for new physiological functions happened only in a limited set of proteases.

In a more general perspective, the comparisons performed in this work suggest that the phylogenetic position of the organism is more decisive than the diet to the evolutionary outcome of peptidase families, at least regarding gene duplication and expansion of gene families. Interestingly, only five peptidase families showed expansions related to hematophagy and 20 showed gene number variations associated with particular orders. This data agrees with the observation that anatomical, physiological and biochemical features of the insect gut follow the phylogeny of the main insect orders (Terra and Ferreira, 2005). Nevertheless, evolutionary convergence regarding expansion or retraction of peptidase gene families seems to be a common event associated with the adaptation for blood feeding. It is remarkable to notice that we observed changes in gene frequencies in three peptidase classes (metallo, aspartic and cysteine peptidases) across four arthropod groups (Acari, Hemiptera, Diptera, Phthiraptera). It is important to notice that these comparisons are biased by the high number of dipterans in the hematophagous group (4 out of 7) and the prevalence of species in Diptera and Hymenoptera when we arrange sequences by taxonomical groups (12 out of 19 species). It will be important in the future to add more species to these sort of analysis, as more arthropod genomes have been sequenced in the last years.

### Multigenic peptidase families expanded in *r. prolixus* genome

The genetic expansion was observed in three families with a large number of peptidases in the *R. prolixus* genome (i.e., A1, pepsin A; C2, calpain-2; and M17, leucyl aminopeptidase), which suggests that these families are potential candidates to be involved in the adaptation process for blood-feeding in Triatomines.

The family A1, pepsin A, should be the only expanded gene family involved directly in the digestion of blood proteins (i.e., Hemoglobin and albumin). The role above is suggested by the presence of a putative signal peptide and active expression in the gut that was observed for most members of this family. This family is typically associated with the lysosomal/endosomal system with catalytic activation by acidification. The most common member of family A1 in insects, cathepsin D, was previously described in triatomine midgut lumen and it was among the most common digestive peptidases in *R. prolixus* (Balczun et al., 2008; Ribeiro et al., 2014). In *R. prolixus*, some of these genes are distributed in a tandem manner on chromosomes, suggesting a role for tandem duplication in this expansion. Putative peptide signal cleavage sites, which are essential structural markers to secrete digestive enzymes into the extracellular environment, were found in the majority of A1 peptidases (13/18), which is consistent with previous records in this family (Balczun et al., 2012). Four A1 peptidase sequences without signal peptides were small and probably incomplete since present less than 50% of residues when compared with other sequences of the family.

Interestingly, most of the members of the A1 protease family in *R. prolixus* grouped in phylogenetic analysis, using both arthropod genomic or hemipteran transcriptomic datasets. The similarity among A1 sequences suggests that duplication of those genes happened after the differentiation of main hemipteran suborders and that this diversification might be an involved adaptation to digestion of blood components in the Triatomine evolutionary lineage. Noteworthy, family A1 gene numbers were not significantly different when hematophagous and non-hematophagous arthropods were compared. This is probably a consequence of the high number of dipteran species in the hematophagous group considered, and highlights this expansion as a particular trait of the Triatominae.

We observed an opposite situation in protease family C1 (Cathepsin B-like or cathepsin L-like cysteine proteinases), another important protease group in *R. prolixus*. A series of biochemical studies have shown that one of the main endopeptidase activities in the midgut of triatomines (including *R. prolixus*) correspond to a cysteine peptidase (Garcia et al., 1978; Garcia and Guimarães, 1979; Houseman and Downe, 1980, 1981; Terra et al., 1988), which probably is a Cathepsin L-like enzyme from family C1 (Terra and Ferreira, 2005). Surprisingly, we did not observe gene expansion in the C1 peptidase gene family in the genome of *R. prolixus*. The absence of a detected expansion could be a result of the stringent criteria used for the definition of gene number increase. For example, values above 90% percentil across the 19 genomes studied will not be produced by few duplications in a large gene family as C1 peptidases, which presented the second highest 90% percentil among all protease families studied here. One possibility is that the recruitment of C1 peptidases in triatomine digestion involved other mechanisms than gene duplication. In this way, we could expect overexpression of C1 genes at the midgut, which was observed in a recent transcriptome analysis (Ribeiro et al., 2014). In this respect, *R. prolixus* C1 peptidases should be analyzed in more detail to understand why they did not follow the same evolutionary tendency of families A1, C2 and M17. In the case of C1 peptidases, the presence of a high number of C1-protease genes seems to be a previous acquisition during the evolution of phytophagous hemipteran ancestors, as *R. prolixus* C1-proteases do not group together in phylogenetic analysis of genomic or transcriptomic datasets. It has been proposed that the diversification of C1-proteases in Hemipterans might be related to the adaptation for the presence of plant inhibitors as phytocystatins in the diet and that A1-proteases might have been recruited for inactivation of these inhibitors in the initial stages of digestions (Pimentel et al., 2017). In this respect, the observed array of C1-proteases and at least part of the amplification observed in the A1 family in *R. prolixus* may be a result of adaptations for phytophagy in the Hemipteran lineage that are previous to the development of blood-sucking behavior.

The other two expanded families (C2 and M17) are commonly described as cytosolic enzymes. Proteases from family C2 (calpains) are highly specific cytosolic proteases involved in remodeling of cytoskeletal/membrane attachments, signal transduction pathways and apoptosis (Ono and Sorimachi, 2012). It is interesting to notice that calpain genes are not common in invertebrates, which contrast with the presence of 15 genes in humans, for example. Besides that, some human calpains (Calpain 8 and 9) are gut-specific and involved in response to chemical stress. Comparison of *R. prolixus* C2 sequences with human calpains (Supplementary Table 15) indicate that the expanded calpain branch in *R. prolixus* genome is composed by classical calpains (similar to calpains 3, 8 and 9), but their functional assignment needs clarification. The observed increase in calpain gene number in hematophagous insects could be related to the necessity of intense intracellular trafficking after a blood meal due to the secretion of induced digestive enzymes and perimicrovilar (or perithophic) membrane. Alternatively, it might be due to the adaptation and signaling against toxic components which are present in the blood meal, as the pro-oxidative heme molecule (Graça-Souza et al., 2006). It is interesting to notice that most *R. prolixus* C2 sequences grouped in the phylogenetic analysis of either genomic or transcriptomic dataset. The grouping suggests that family C2 expansion occurred after diversification of hemipteran suborders, being a putative adaptation for blood feeding. In some respect, it was unexpected to find adaptations for the blood feeding involving intracellular proteases in *R. prolixus*. Independently of the specific role of *R. prolixus* C2 proteases, it is noteworthy that changes in the proteolytic genes involved in adaptations for blood feeding in Triatomines are not restricted to digestive enzymes.

Family M17 (leucyl aminopeptidases) is associated with the breakdown of peptide products of intracellular peptidases (Lowther and Matthews, 2002). In humans, these enzymes are involved in the processing of peptides generated by the proteasome, specially by the Major Histocompatibility Complex type I (Saveanu et al., 2002). Despite the cytosolic nature of the majority of the characterized members of the M17 peptide family, these peptidases were already described as important in digestion in a series of organisms. Interestingly, M17 peptidases are necessary for hemoglobin digestion in the parasites *Plasmodium falciparum* and *P. vivax* (Lee et al., 2010; Skinner-Adams et al., 2010), *Schistosoma mansoni* and *S. japonicum* (McCarthy et al., 2004), in the tick *Haemaphysalis longicornis* (Hatta et al., 2006) and in the mite *Psoroptes* spp. (Nisbet and Billingsley, 2002). Besides that, M17 leucine peptidases were already incriminated as digestive enzymes in the midgut of coleopteran (*Morimus funereus*; Bozić et al., 2008) and dipteran species (*Anopheles darlingi*, Okuda et al., 2005). In this respect, some insects may present digestive leucine aminopeptidases which differ in specificity and structure from the usual M1 digestive aminopeptidase (aminopeptidase N) that are found in all insect orders (Terra and Ferreira, 2005). Expansion of the M17 gene family was also documented in *Drosophila melanogaster*, but in this case, the proteins are secreted in the sperm, and many members have lost enzymatic activity (Dorus et al., 2011). It is noteworthy that several *R. prolixus* M17 sequences present putative signal peptide sequences, or non-cytosolic and transmembrane topologies. More studies are needed to unravel the exact participation of *R. prolixus* M17 genes in digestion, as these proteins could be involved in intracellular processing of secreted proteins and vesicular trafficking, or directly in blood protein hydrolysis.

Both C2 and M17 families presented a significantly higher number of genes in hematophagous than non-hematophagous arthropods, which suggest a possible non-digestive role or a secondary digestive role in the adaptations for blood feeding despite the independent origins of this feeding behavior. However, it would be important to ascertain if the A1, C2 and M17 expansions occurred in other members of the triatominae family or have been restricted to the species *R. prolixus* and further studies will be required to corroborate their role in *R. prolixus* and other hematophagous insects.

### Horizontal transfer of peptidase genes from microorganisms to *r. prolixus* genome

This study suggests horizontal gene transfer to the *R. prolixus* genome of six genes belonging to 3 different peptidase families (M74, S24, and S29) associated with bacteria or viruses. Peptidases from these families are not commonly found in any eukaryote organism, but most of them were found in genomic regions surrounded by genes associated with insect functions and metabolism. Their presence was confirmed by amplification of genomic DNA from *R. prolixus* and they were found to be expressed in the gut of blood-fed adults by RT-PCR.

The two peptidases of the family S29, hepacivirin, grouped in the phylogenetic analysis with sequences obtained from hepatitis C viruses types 3, 4, and 6. It is unclear how genes might be transferred to the insect genome from vertebrate viruses, but infection during blood feeding on a contaminated vertebrate host seems to be the most probable route. Recently, the putative role of arthropods in hepatitis virus transmission has been considered (Houldsworth, 2017). Hepatitis virus has been found to be stable and viable for at least 1 month in the gut of bed bugs (*Cymex lectularius* and *C. hemipterus*) and *R. prolixus*, with the virus being detected in feces and transmitted trans stadially (Blow et al., 2001; Silverman et al., 2001). Besides that, Hepatitis virus has been detected in mosquitoes in endemic areas, and it has been found that Hepatitis virus efficiently replicates in cultures of mosquito cells (Fallecker et al., 2017). The possibility of Hepatitis transmission by arthopods has been hindered by the ignorance of natural reservoirs of the virus or documentation of prevalence of the virus in putative vectors (Houldsworth, 2017). In any scenario, the presence of active hepacivirin genes in the *R prolixus* genome suggests that the interaction between Hepatitis-like virus and insects might be evolutionary old. Besides that, this should be taken into account when performing the inquiry of this virus in insect population based on detection of virus DNA or RNA, as it might lead to false positive results. Further analysis will be required to identify the evolutionary and epidemiological meaning, physiological role and mechanisms involved in the transfer of these two genes to the genome of *R. prolixus*, as they have no apparent role in the triatomine metabolism.

The gene RPRC003168 was identified as a peptidase of the family M74, murein endopeptidase. This gene is located in a small contig of the *R. prolixus* genome with a low guaranty of being a true insertion in this genome. However, the topology prediction of the protein indicates the existence of signal peptide that it is a eukaryotic structure and several transmembrane domains surrounded by cytoplasmatic and non-cytoplasmatic regions. Besides that, its presence and expression were confirmed by PCR and RT-PCR of genomic DNA and RNAm from blood-fed adults, respectively. This peptidase is closely related with genes from Gram-negative bacteria such as *Dickeya* spp. and *Yersinia* spp. that may be associated with hematophagous arthropods. The similarity reinforces the hypothesis of horizontal gene transfer from microorganism to the *R. prolixus* genome, since this type of bacteria has been previously implicated in horizontal gene transfer in arthropods (Dillon and Dillon, 2004). This peptidase family is associated with synthesis and lysis of bacterial cell walls and may play a fundamental role in the defense of *R. prolixus* against bacterial pathogens or digestion of bacterial symbionts which develop in the midgut after blood feeding (Eichler and Schaub, 2002). A further analysis focusing expression/function of this gene/protein in *R. prolixus* will be required to confirm these roles.

Horizontal gene transfer might also have occurred in two peptidases of the family S24, repressor LexA (RPRC002798, RPRC005865). This family has not a digestive function, and it was described in a few eukaryote organisms (e.g., cnidarian, mammals, and reptile). However, the comparative analysis of the 19 arthropod genomes only found peptidases of this family in hemipteran species (*Ac. pisum* and *R. prolixus*). In this case, the horizontal gene transfer might have been occurred in a primitive Hemiptera, allowing divergence of the peptidase among Hemiptera such as observed between *Ac. pisum* and *R. prolixus*. This hypothesis would require further studies comparing the S24 peptidases across the order Hemiptera.

The importance of microorganisms in the development and physiology of triatomines is recognized since the isolation of the bacterial symbiont *Nocardia rhodnii* (later known as *Rhodococcus rhodnii*) from *R. prolixus* (Wigglesworth, 1936). *R. prolixus* does not fullfill its development in the absence of this actinomycete, and for many years it was believed that *Rh. rhodnii* is responsible for the production of several vitamins which are essential for the insect, specially of the complex B (Lake and Friend, 1968). However, there is compelling evidence that in fact, the symbiosis relies on a more complex metabolic complementation (Hill et al., 1976). After a blood meal, the symbiont can correspond to 5% of the insect dry weight (Hill et al., 1976). Despite the importance of actinomycetes for triatomines, recent studies have shown that their gut microbiota is a very heterogeneous and variable community, especially when we consider insects from the field (da Mota et al., 2012). Symbiosis with intestinal bacteria seems to be evolutionarily linked with the development of phytophagy and hematophagy, as hemipteran predators do not have this dependence (Buchner, 1965). The presence of a multitude of bacteria in the intestinal tract and the coevolution of gut bacteria and insect to stablish successful symbiotic pairs seems to be an appropriate scenario for the horizontal transference of genetic material between these organisms. It is interesting to notice that the two putative bacterial sources for genetic transfer to the *R. prolixus* genome are species which have well documented associations with insects. *Yersinia* is usually transmitted by fleas, being acquired from vertebrates and colonizing the insect midgut during hematophagy (Chouikha and Hinnebusch, 2012). In this case, it is very likely that the encounter of triatomines with this bacteria followed the same route, and that the acquisition of *Yersinia* genetic material by *Rhodnius* occurred after the development of hematophagy. In this scenario, this insertion might not be present in other triatomines genomes, being a putative marker of evolutionary distance between triatomine groups. On the other hand, *Dickeyia* is a plant pathogen, normally acquired and pathogenic for plant aphids (Costechareyre et al., 2013). In this case, it is more likely that this genetic transfer has occurred in some phytophagous ancestor of the triatominae lineage, located after the divergence between them and aphids, represented in our analysis by the *Ac. pisum* genome. In this way, the acquisition of peptidase genes from bacteria might have had a major role in the evolutionary establishment of blood feeding in triatomines.

The exchange of genetic material between different species inside the gut of *R. prolixus* may have a significant impact on the development of control strategies for the transmission of Chagas Disease. Many recent studies have proposed paratransgenesis as a promising strategy for manipulating the gut environment of *R. prolixus*, making it refractory to *T. cruzi* parasites. A major concern associated with this approach is the horizontal gene transfer between bacteria in the insect gut, which was already documented in fleas (Hinnebusch et al., 2002). The impact of horizontal gene transfer was already assessed theoretically and in test trials (Matthews et al., 2011), but our observations point that transfer to the insect genome should also be considered in this scenario.

### Diversification of *r. prolixus* aspartyl peptidases of family A1

Several hemipteran digestive aspartil endopeptidases were already classified as Cathepsin D-like enzymes, including *R. prolixus* activities (Houseman and Downe, 1980, 1981, 1982, 1983; Terra et al., 1988; Terra and Ferreira, 2005). The most accepted theory of the evolution of these enzymes accounts for a loss of main proteases that were probably serinic in the hemipteran ancestor, due to the adaptation to feeding on plant sap. This loss was concomitant to the loss of peritrophic membrane and counter current water fluxes in the midgut, two very-well documented features of the hemipteran midgut. It was postulated that, after the evolutionary loss of serine peptidases as trypsin and chymotrypsin, later adaptations were necessary for digestion of proteins as proteinaceous inhibitors or defense proteins from plants, or proteins from food of predators and blood-sucking species. In this scenario, the main adaptation would be the duplication of lysosomal protease genes and the recruitment of copies for luminal secretion and activity against food molecules (Terra and Ferreira, 2005).

Despite the consistency of this theory and all the gathered data involving it, it is important to consider that the original classification of hemipteran digestive aspartyl endopeptidases as Cathepsin D-like enzymes was performed with a much reduced amount of biochemical and no protein sequence data. It is critical to notice that there are very few diagnostic features that allow differentiating between pepsin and cathepsin D, the main digestive aspartyl peptidases already described in animals. Three important properties that were mapped in cathepsin D that are different from Pepsin are the less acidic optimum pH (between 3.5 and 5), the presence of a proline loop and activity against hemoglobin ten times higher than against albumin (Fusek et al., 2014). Unfortunately, these features are not completely clear in the case of *R. prolixus* activities, due to their highly acidic optimum pH and sobreposition with other protease activities and inhibition features (Terra et al., 1988). Besides that, at the time of their assignment as cathepsin D-like proteases, some key enzymological (regarding activity against hemoglobin vs. albumin) and sequence data were not available. In this work, we observed that any of the A1 peptidase sequences in the *R. prolixus* genome does not bear the Cathepsin D proline loop, and preliminary data from our group show activity levels against hemoglobin very similar to those observed against albumin (ratio 1.1 ± 0.2, data not shown), properties that make these enzymes more similar to Pepsin. Because of this apparent contradiction, we decided to analyse in more detail the properties of *R. prolixus* A1 peptidase sequences. We compared them directly to some archetypes of this protein family, namely Pepsin A, Renin and Cathepsin D. What emerges from these comparisons is that from all the characteristics observed (including glycosilations and active site structure) the most discriminatory between Pepsin and Cathepsin D was the frequency and distribution of basic amino acid residues at the protein surface. In this respect *R. prolixus* sequences showed an exemplar Cathepsin D-like structure. This analysis not only reinforces the classification of hemipteran sequences as Cathepsin D-like proteins using clear sequence markers but might also help to extend this feature to other insect species, corroborating the hypothesis about the lysosomal origin of midgut aspartic endopeptidases in Hemiptera and Coleoptera (Terra and Ferreira, 2005). Notably, a higher frequency of surface basic residues correlates with a less acidic optimum pH in A1 aspartic peptidases (Fusek et al., 2014), and this might be in partial accordance with the less acidic values observed for the hemipteran activities (3–4) than Pepsin itself (around 2).

The high variability that was observed in A1 *R. prolixus* peptidases may be interpreted as a consequence of a previously diversified background present in the ancestor of the Triatomine branch. As discussed above, the putative reason for this previous round of genetic expansion was the adaptation for the presence of protease inhibitors as phytocystatins in an herbivorous ancestor. However, the phylogenetic analysis of A1 sequences suggests that family A1 suffered amplification during the evolution of Triatominae and its adaptation for blood-feeding. In this respect, it is noteworthy that the active site topology of *R. prolixus* proteases are strikingly diverse, not mimicking any of these expected homologous counterparts as cathepsin D or pepsin. In this way, it is very challenging to foresee the substrate specificities of those enzymes, but we can expect a significant variation of structures for binding and hydrolysis. It is very likely that a major evolutionary pressure for active site variation in this protease family is to furnish to these blood-sucking insects the most diverse array of proteases to cope efficiently with a diverse universe of protein substrates. Substrate diversity in the case of triatomines might occur at two different levels at least. Firstly, we must consider the variety of proteins in blood as hemoglobin, albumin, lipoproteins, imunoglobulins, surface antigens, ferritins, fibrin, plasmin, components of the complement cascade and so on. Secondly, Triatomines are generalist blood-feeders and normally take blood meals from different vertebrates across their life time.

This scenario agrees with the complexity of the blood with several defense molecules that may present high intra-specific diversity (Cederlund et al., 2011) and the ability of *R. prolixus* in feeding in different vertebrates that promote a variation at inter-specific level (Peña et al., 2012). Beyond this complexity we should consider the observation that *R. prolixus* artificially reared with blood from different animal sources has extreme variations in development and reproductive performance (Gomes et al., 1990). Moreover, the observation that insects collected from field normally fed on one or two different blood sources (Peña et al., 2012) suggesting that the proteases from the insect during evolution might have developed maximal activities against a broad spectrum of protein sequences. In mosquito was observed a similar pattern with high diversification in the surface region associated with inhibitors in the genetic expansion of trypsin genes that present similar digestion function in the digestion of blood proteins (Wu et al., 2009). In Lepidoptera, coevolution of insect serine proteinases and similar proteins have led to the expansion of digestive serine proteinase genes and variation in sensitivity against plant protease inhibitors (de Oliveira et al., 2013). Further biochemical studies focusing on the digestive role of these enzymes and their activities on different blood molecules will be required to assure the physiological importance of A1 protease gene expansion in *R. prolixus* genome.

Importantly, we were able to confirm the actual presence in the genome and the gut expression of a complete and highly diversified array of *R. prolixus* proteases. In the specific case of A1 peptidases, the expression of 17 members of this family was previously reported in the transcriptome of *R. prolixus* (Ribeiro et al., 2014), and we were able to describe and partially characterize two more sequences of this protease family (RPRC002696 and RPRC012664) (Supplementary Table 14). For the other families considered, the coverage of the transcriptome was lower, as there were reported only 12, 3, 6 and one sequences for the families C1, C2, M17, and S24, respectively, and no sequences of families M74 and S28 (Supplementary Table 14). We were able to confirm the expression of these genes above and find more 5, 9, 7 and two genes in the respective families C1, C2, M17, and S24, several of them also being expressed in the *R. prolixus* gut (Supplementary Table 14). Besides that, transcripts from families M74 and S29 were described for the first time in *R. prolixus*. This outstanding diversity of proteases might be related not only to adaptations for the digestion of different blood proteins but also to differential expression and roles in the various compartments of the gut (e.g., salivary glands, anterior and posterior midgut and hindgut), as well as at the various physiological moments that the insect and its gut face during development (e.g., starving, initial, intermediary and final stages of blood digestion, and molting). In this way, aspects of the regulation of expression of these proteases are of particular concern. We expect that the mapping of peptidase gene families which are putatively related to the evolutionary adaptation for blood feeding in triatomines can help to orientate more detailed studies on the biochemical and physiological basis of digestion in this insect group and contribute to design new strategies for the control of transmission of Chagas Disease.

## Author contributions

FG, RD, EdSG, and PA conceived and designed the study. VD and RM gave important intellectual contributions. BH, BG, SdC, and CdSM performed the experiments and data analysis.

### Conflict of interest statement

The reviewer [MGP] declared a shared affiliation, though no other collaboration, with several of the authors [BH, BG, SdC, CdSM, EdSG, PA, and FG] to the handling Editor. The other authors declare that the research was conducted in the absence of any commercial or financial relationships that could be construed as a potential conflict of interest.
