# Supplementary material for: Genome Wide Mapping of Peptidases in Rhodnius prolixus: Identification of Protease Gene Duplications, Horizontally Transferred Proteases and Analysis of Peptidase A1 Structures, with Considerations on Their Role in the Evolution of Hematophagy in Triatominae
Source: Front Physiol. 2017 Dec 12;8:1051. doi: 10.3389/fphys.2017.01051 (PMC5736985; doi:10.3389/fphys.2017.01051)
Supplement: Supplementary file 2 [file Image2.PDF]

Supplementary Material

Genome wide mapping of peptidases in *Rhodnius prolixus*: identification of protease gene duplications, horizontally transferred proteases and analysis of peptidase A1 structures, with considerations on their role in the evolution of hematophagy in Triatominae

Bianca Santos Henriques, Bruno Gomes, Caroline da Silva Moraes, Samara Graciane Costa, Rafael Dias Mesquita, Viv Maureen Dillon, Eloi de Souza Garcia, Patricia Azambuja, Roderick James Dillon, Fernando Ariel Genta*

*** Correspondence:** Corresponding Author: genta@ioc.fiocruz.br or gentafernando@gmail.com

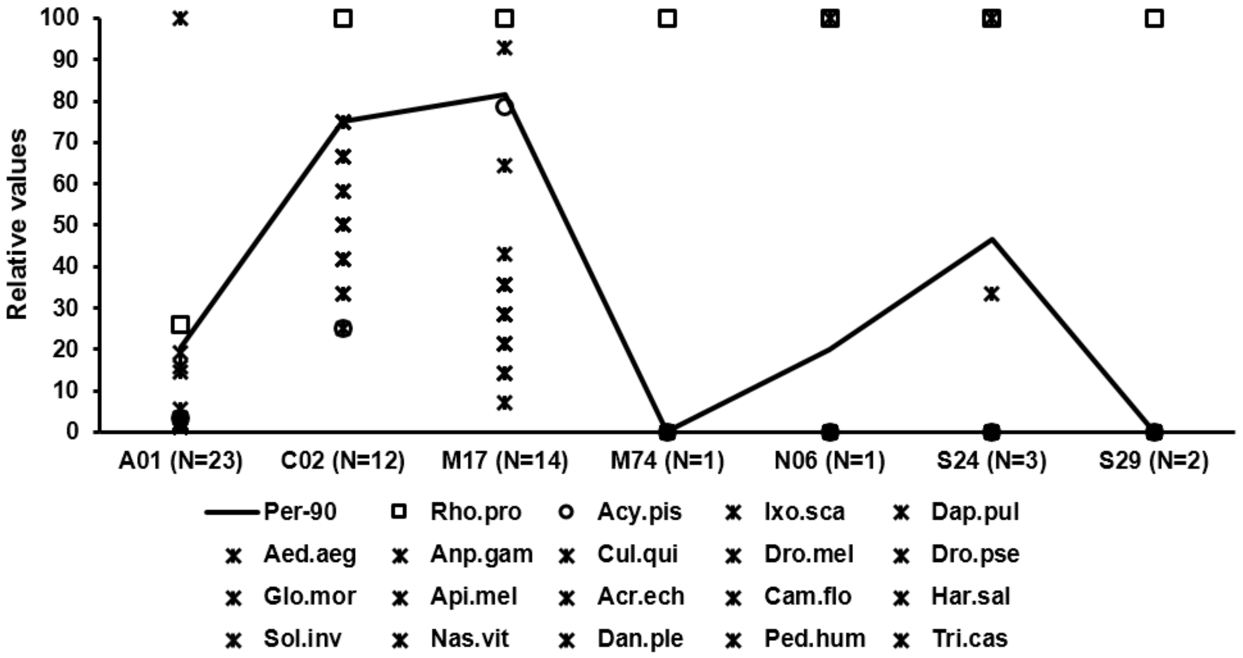

Supplementary Figure 2. Peptidase relative gene number in families with a higher number of coding genes in *R. prolixus*. Relative values: frequency (%) related to the larger number of coding genes among 19 arthropod species in each peptidase family. Values in brackets represent absolute frequencies of coding genes in *R. prolixus*. Per 90: Percentil 90% of the frequency of coding genes distribution among 19 arthropod species. Rho.pro, Acy.pis, Ixo.sca, Dap.pul, Tri.cas, Aed.aeg, Anp.gam, Cul.qui, Dro.mel, Dro.pse, Glo.mor, Api.mel, Acr.ech, Cam.flo, Har.sal, Sol.inv, Nas.vit, Dan.ple and Ped.hum are the respective frequency of coding genes in *Rhodnius prolixus*, *Acyrthosiphon pisum*, *Ixodes scapularis*, *Daphnia. pulex*, *Tribolium castaneum*, *Aedes aegypti*, *Anopheles gambiae*, *Culex*

quinquefasciatus, *Drosophila melanogaster*, *Drosophila pseudoobscura*, *Glossina. morsitans*, *Apis mellifera*, *Acromyrmex echinatior*, *Camponotus floridanus*, *Harpegnathos saltator*, *Solenopsis invicta, Nasonia vitripennis, Danaus plexippus* and *Pediculus humanus*.
