# Supplementary material for: Genome Wide Mapping of Peptidases in Rhodnius prolixus: Identification of Protease Gene Duplications, Horizontally Transferred Proteases and Analysis of Peptidase A1 Structures, with Considerations on Their Role in the Evolution of Hematophagy in Triatominae
Source: Front Physiol. 2017 Dec 12;8:1051. doi: 10.3389/fphys.2017.01051 (PMC5736985; doi:10.3389/fphys.2017.01051)
Supplement: Supplementary file 3 [file Image3.PDF]

frontiers

Supplementary Material

Genome wide mapping of peptidases in *Rhodnius prolixus*: identification of protease gene duplications, horizontally transferred proteases and analysis of peptidase A1 structures, with considerations on their role in the evolution of hematophagy in Triatominae

Bianca Santos Henriques, Bruno Gomes, Caroline da Silva Moraes, Samara Graciane Costa, Rafael Dias Mesquita, Viv Maureen Dillon, Eloi de Souza Garcia, Patricia Azambuja, Roderick James Dillon, Fernando Ariel Genta*

*** Correspondence:** Corresponding Author: genta@ioc.fiocruz.br or gentafernando@gmail.com

A

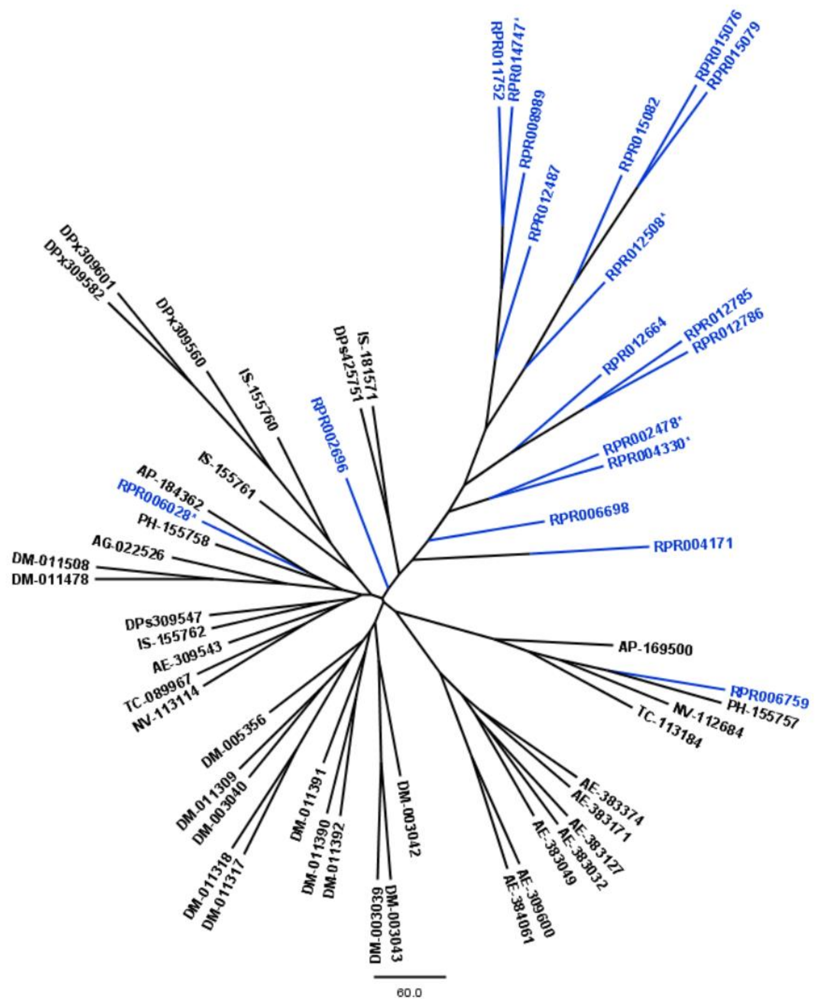

B

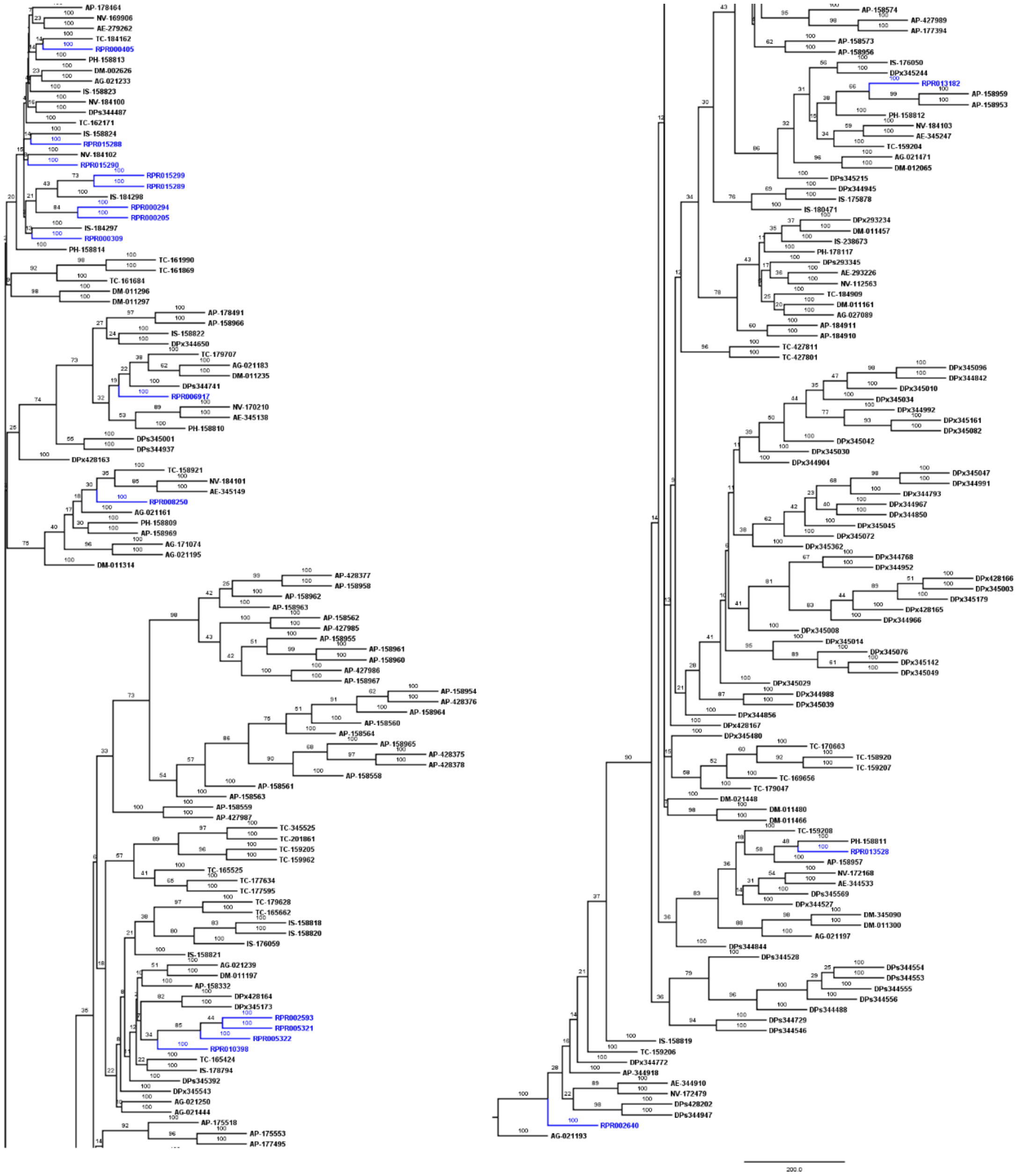

Supplementary Figure 3. Phylogenetic trees of amino acid sequences from peptidases belonging to families A1 and C1, present in 18 arthropod genomes plus *Rhodnius prolixus*. A: Family A1 (Pepsin A or Cathepsin D-like proteins). B: Family C1 (Papain). Trees gather sequences from 19 arthropod species. Blue: branches with *Rh. prolixus* peptidases; Black: branches with peptidases from 18 arthropod species; ACY: *Acyrthosiphon pisum*, IXO: *Ixodes scapularis*, DAP: *Daphnia. pulex*, TRI: *Tribolium castaneum*, AED: *Aedes aegypti*, ANO: *Anopheles gambiae*, CUL: *Culex quinquefasciatus*, DRm: *Drosophila melanogaster*, DRp: *Drosophila pseudoobscura*, GLO: *Glossina. morsitans*, API: *Apis mellifera*, ACR: *Acromyrmex echinatior*, CAM: *Camponotus floridanus*, HAR: *Harpegnathos saltator*, SOL: *Solenopsis invicta*, NAS: *Nasonia vitripennis*, DAN: *Danaus plexippus* and PED: *Pediculus humanus*. See Material and Methods for details.
