# Supplementary material for: Genome Wide Mapping of Peptidases in Rhodnius prolixus: Identification of Protease Gene Duplications, Horizontally Transferred Proteases and Analysis of Peptidase A1 Structures, with Considerations on Their Role in the Evolution of Hematophagy in Triatominae
Source: Front Physiol. 2017 Dec 12;8:1051. doi: 10.3389/fphys.2017.01051 (PMC5736985; doi:10.3389/fphys.2017.01051)
Supplement: Supplementary file 4 [file Image4.PDF]

frontiers

Supplementary Material

Genome wide mapping of peptidases in *Rhodnius prolixus*: identification of protease gene duplications, horizontally transferred proteases and analysis of peptidase A1 structures, with considerations on their role in the evolution of hematophagy in Triatominae

Bianca Santos Henriques, Bruno Gomes, Caroline da Silva Moraes, Samara Graciane Costa, Rafael Dias Mesquita, Viv Maureen Dillon, Eloi de Souza Garcia, Patricia Azambuja, Roderick James Dillon, Fernando Ariel Genta*

*** Correspondence:** Corresponding Author: genta@ioc.fiocruz.br or gentafernando@gmail.com

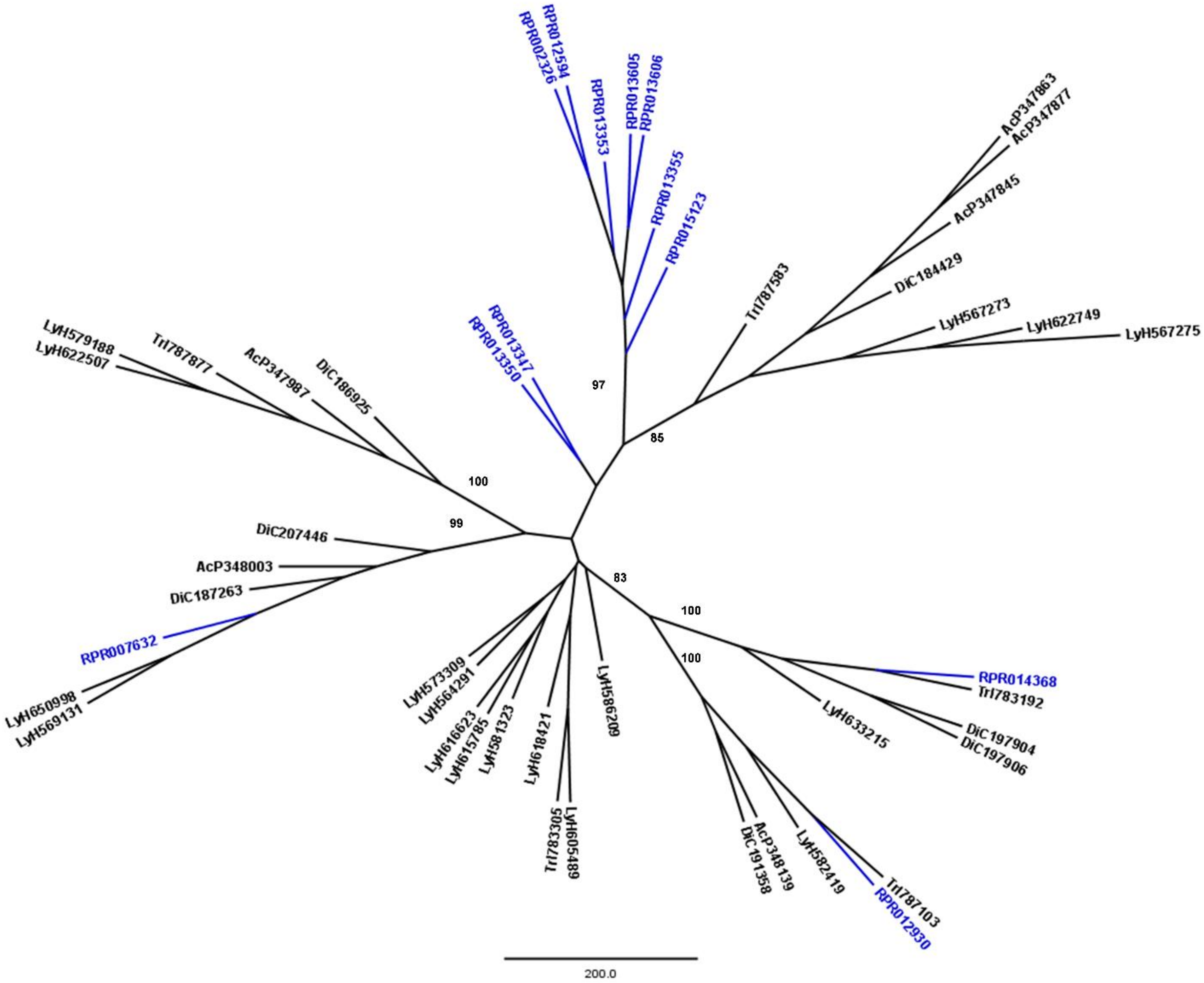

Supplementary Figure 4. Phylogenetic trees of amino acid sequences from the peptidase family C2 (Calpains) in Hemiptera. Blue: branches with *Rhodnius prolixus* peptidases (RPR); Black: branches with peptidases from other species; TrI: *Triatoma infestans* (hemathophagus Heteroptera); LyH: *Lygus hesperus* (Heteroptera); AcP: *Acyrthosiphon pisum* (Aphidiformes); DiC: *Diaphorina citri* (Psylliformes). Basal bootstrap above 60 are shown in the tree.
