# Supplementary material for: Genome Wide Mapping of Peptidases in Rhodnius prolixus: Identification of Protease Gene Duplications, Horizontally Transferred Proteases and Analysis of Peptidase A1 Structures, with Considerations on Their Role in the Evolution of Hematophagy in Triatominae
Source: Front Physiol. 2017 Dec 12;8:1051. doi: 10.3389/fphys.2017.01051 (PMC5736985; doi:10.3389/fphys.2017.01051)
Supplement: Supplementary file 6 [file Image6.PDF]

Supplementary Material

Genome wide mapping of peptidases in *Rhodnius prolixus*: identification of protease gene duplications, horizontally transferred proteases and analysis of peptidase A1 structures, with considerations on their role in the evolution of hematophagy in Triatominae

Bianca Santos Henriques, Bruno Gomes, Caroline da Silva Moraes, Samara Graciane Costa, Rafael Dias Mesquita, Viv Maureen Dillon, Eloi de Souza Garcia, Patricia Azambuja, Roderick James Dillon, Fernando Ariel Genta*

*** Correspondence:** Corresponding Author: genta@ioc.fiocruz.br or gentafernando@gmail.com

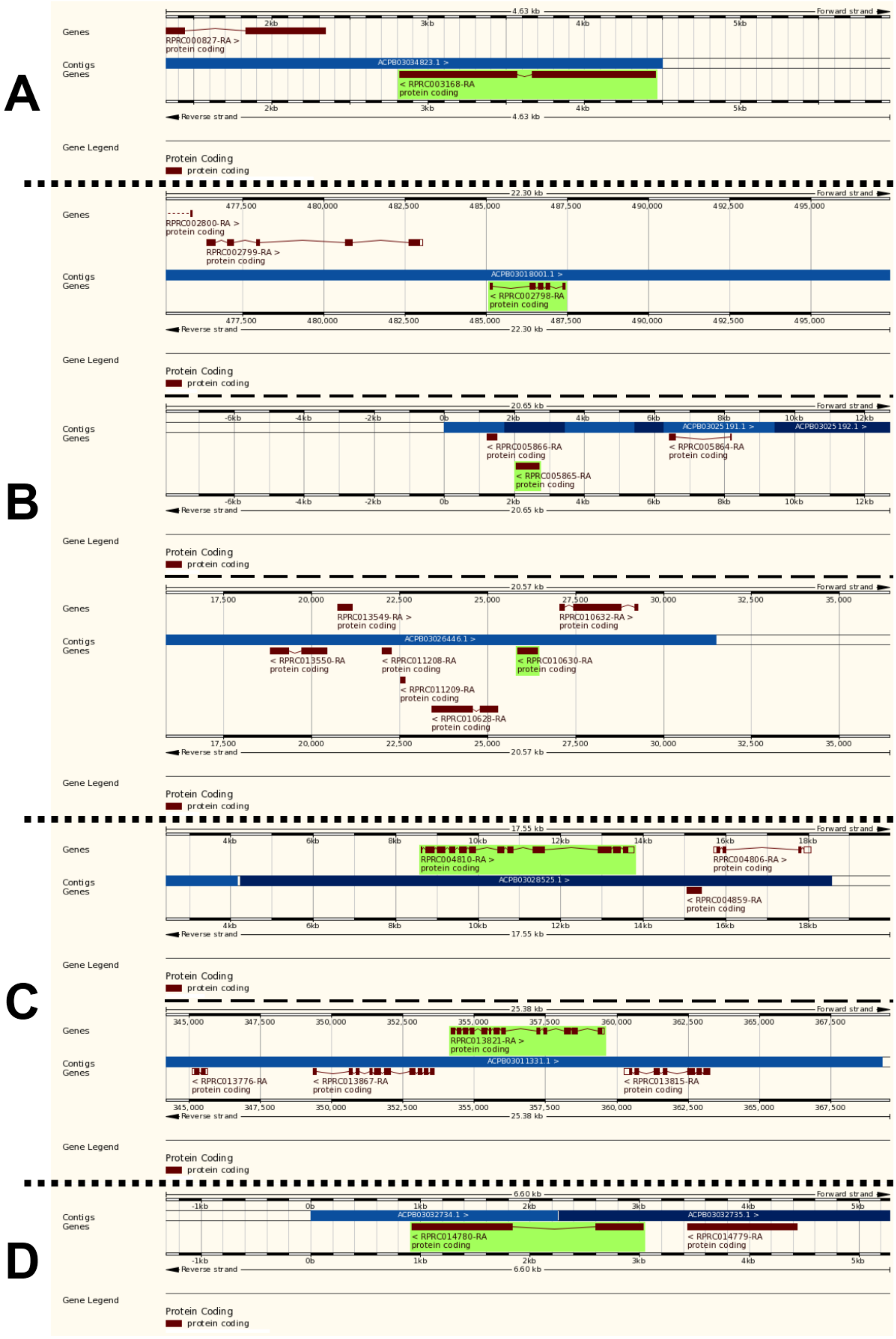
A
B
C
D
Genes
Contigs
Genes
Gene Legend
Protein Coding
protein coding
Forward strand
Reverse strand
4.63 kb
RPRC000827-RA >
ACPB03034823.1 >
< RPRC003168-RA
22.30 kb
RPRC002800-RA >
RPRC002799-RA >
ACPB03018001.1 >
< RPRC002798-RA
20.65 kb
ACPB03025191.1 >
ACPB03025192.1 >
< RPRC005866-RA
< RPRC005864-RA
< RPRC005865-RA
20.57 kb
RPRC013549-RA >
RPRC010632-RA >
ACPB03026446.1 >
< RPRC013550-RA
< RPRC011208-RA
< RPRC010630-RA
< RPRC011209-RA
< RPRC010628-RA
17.55 kb
RPRC004810-RA >
RPRC004806-RA >
ACPB03028525.1 >
< RPRC004859-RA
25.38 kb
RPRC013821-RA >
ACPB03011331.1 >
< RPRC013776-RA
< RPRC013867-RA
< RPRC013815-RA
6.60 kb
ACPB03032734.1 >
ACPB03032735.1 >
< RPRC014780-RA
< RPRC014779-RA

Supplementary Figure 6. Putative genomic regions of *Rhodnius prolixus* corresponding to sequences of four peptidase families which are typical from microorganisms (i.e. S24, M74, S29 and N6). A: Peptidase Family M74 (i.e. RPRC003168). B: Peptidase Family S24 (i.e. RPRC002798, RPRC005865, RPRC010630). C: Peptidase Family S29 (i.e. RPRC004810, RPRC013821). D: Peptidase Family N6 (i.e. RPRC014780).
