# Supplementary material for: Genome Wide Mapping of Peptidases in Rhodnius prolixus: Identification of Protease Gene Duplications, Horizontally Transferred Proteases and Analysis of Peptidase A1 Structures, with Considerations on Their Role in the Evolution of Hematophagy in Triatominae
Source: Front Physiol. 2017 Dec 12;8:1051. doi: 10.3389/fphys.2017.01051 (PMC5736985; doi:10.3389/fphys.2017.01051)
Supplement: Supplementary file 8 [file Image8.PDF]

Supplementary Material

Genome wide mapping of peptidases in *Rhodnius prolixus*: identification of protease gene duplications, horizontally transferred proteases and analysis of peptidase A1 structures, with considerations on their role in the evolution of hematophagy in Triatominae

Bianca Santos Henriques, Bruno Gomes, Caroline da Silva Moraes, Samara Graciane Costa, Rafael Dias Mesquita, Viv Maureen Dillon, Eloi de Souza Garcia, Patricia Azambuja, Roderick James Dillon, Fernando Ariel Genta*

*** Correspondence:** Corresponding Author: genta@ioc.fiocruz.br or gentafernando@gmail.com

Genes	Genomic DNA	cDNA
RPRC002696	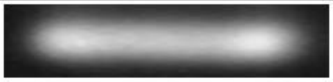	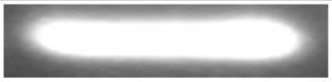
RPRC004171	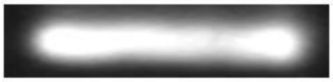	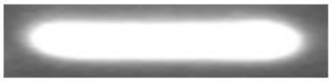
RPRC008989	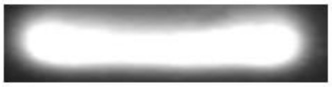	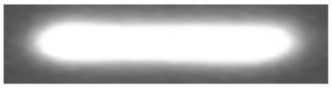
RPRC010954	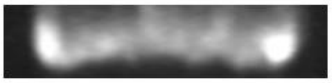	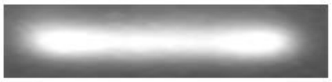
RPRC012487	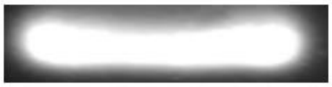	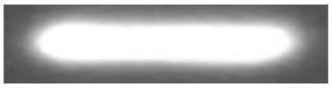
RPRC012785	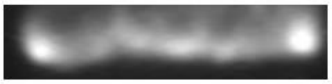	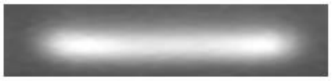
RPRC006698	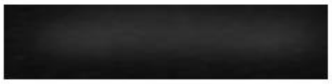	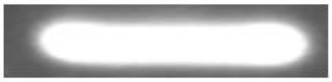
RPRC011752	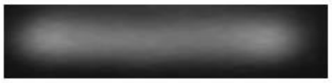	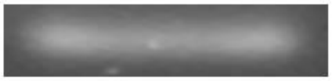
RPRC012664	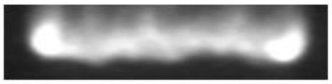	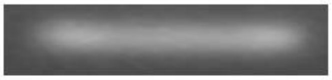
RPRC012786	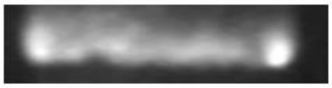	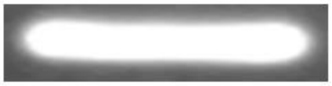
RPRC015079	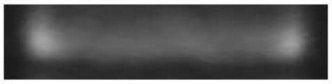	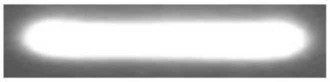
RPRC002478	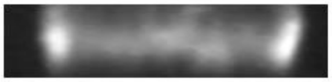	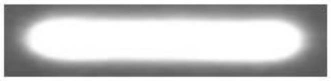
RPRC004330	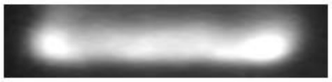	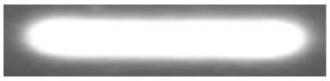
RPRC006028	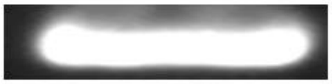	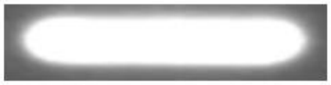
RPRC006759	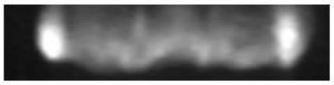	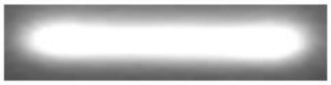
RPRC012508	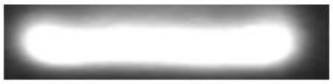	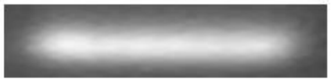
RPRC014747	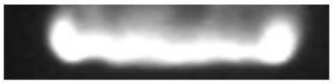	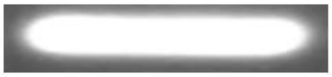
RPRC015076	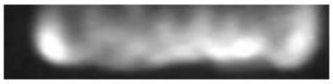	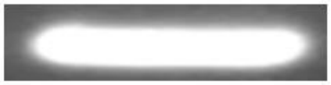
RPRC015082	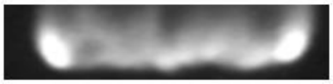	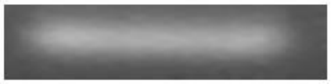

B

Genes	Genomic DNA	cDNA
RPRC002326	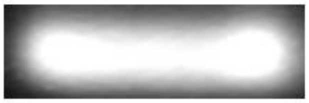	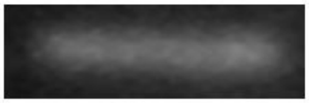
RPRC012930	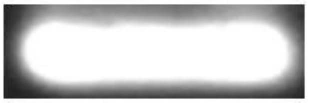	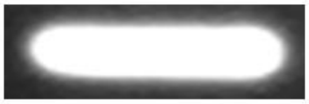
RPRC013350	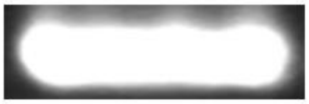	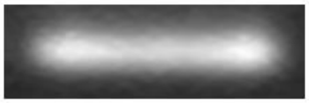
RPRC013606	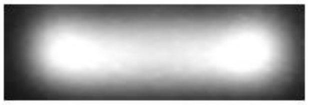	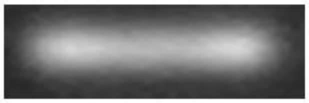
RPRC014368	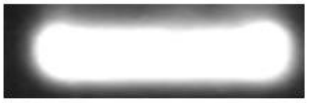	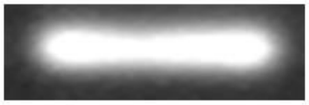
RPRC007632	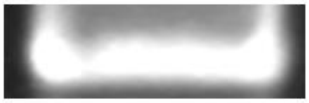	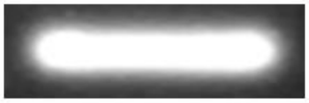
RPRC012594	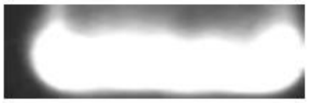	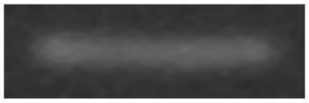
RPRC013347	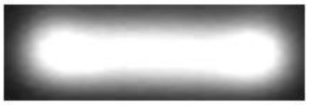	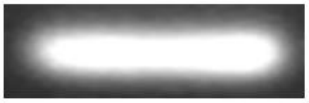
RPRC013353	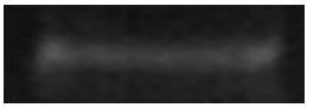	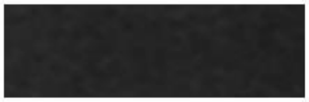
RPRC013355	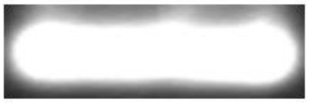	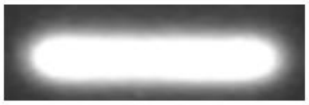
RPRC013605	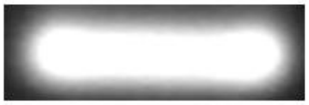	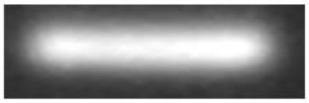
RPRC015123	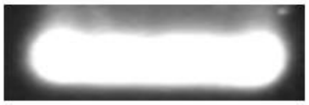	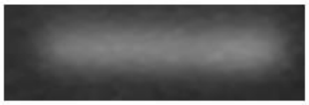

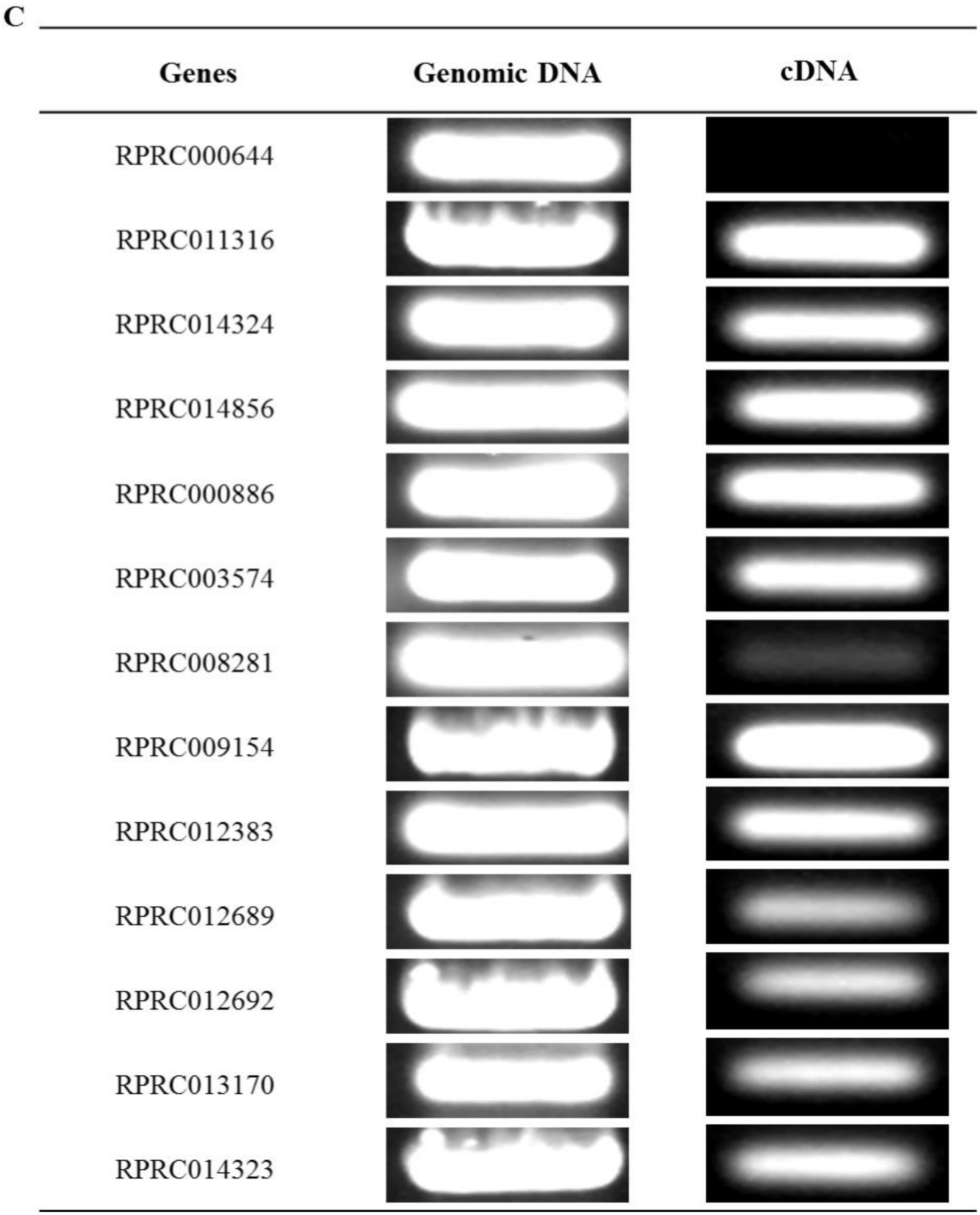
C
Genes
Genomic DNA
cDNA
RPRC000644
RPRC011316
RPRC014324
RPRC014856
RPRC000886
RPRC003574
RPRC008281
RPRC009154
RPRC012383
RPRC012689
RPRC012692
RPRC013170
RPRC014323

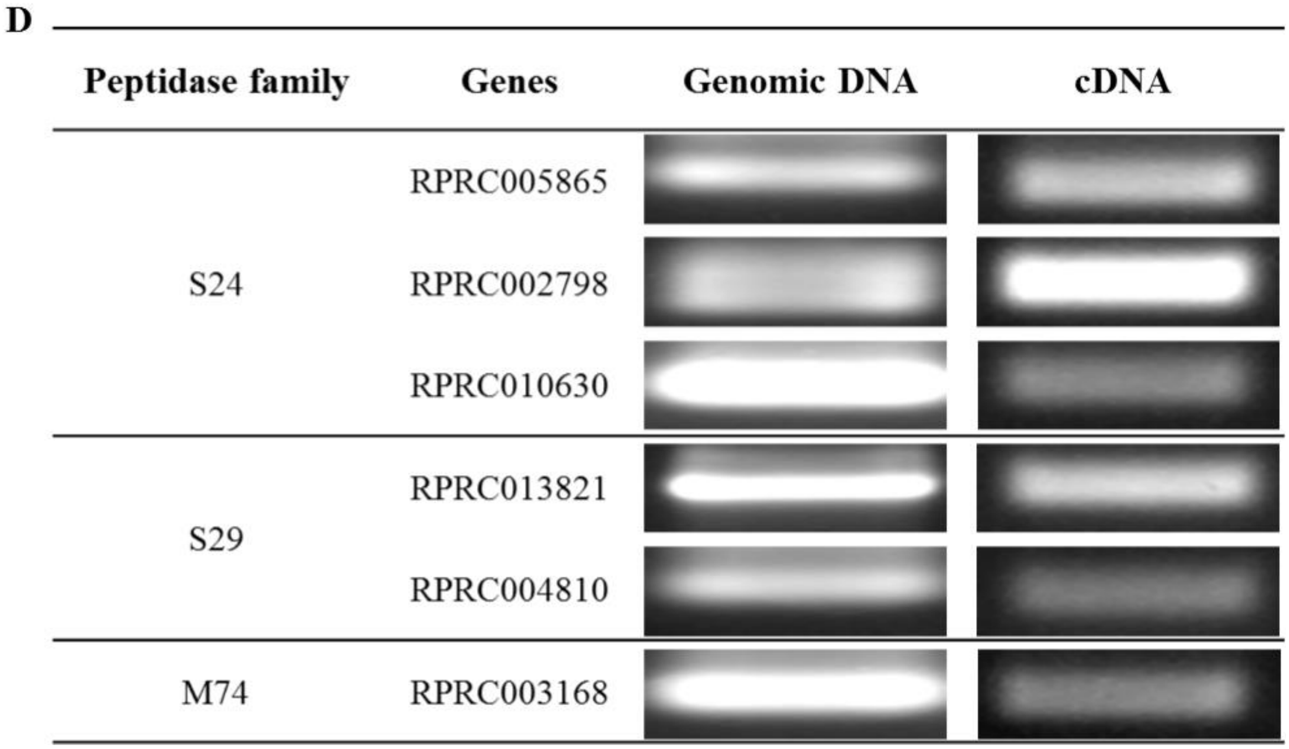

Supplementary Figure 8. PCR and RT-PCR amplification from *Rhodnius prolixus* corresponding to genes of protease families: **A** - A1 (Papain or Cathepsin D-like proteins), **B** - C2 (Calpains), **C** - M17 (Leucine Aminopeptidases), and **D** - M74, S24 and S29, corresponding to horizontally transferred putative genes of bacterial origin.
