# Supplementary material for: Genome Wide Mapping of Peptidases in Rhodnius prolixus: Identification of Protease Gene Duplications, Horizontally Transferred Proteases and Analysis of Peptidase A1 Structures, with Considerations on Their Role in the Evolution of Hematophagy in Triatominae
Source: Front Physiol. 2017 Dec 12;8:1051. doi: 10.3389/fphys.2017.01051 (PMC5736985; doi:10.3389/fphys.2017.01051)
Supplement: Supplementary file 10 [file Image10.PDF]

Supplementary Material

Genome wide mapping of peptidases in *Rhodnius prolixus*: identification of protease gene duplications, horizontally transferred proteases and analysis of peptidase A1 structures, with considerations on their role in the evolution of hematophagy in Triatominae

Bianca Santos Henriques, Bruno Gomes, Caroline da Silva Moraes, Samara Graciane Costa, Rafael Dias Mesquita, Viv Maureen Dillon, Eloi de Souza Garcia, Patricia Azambuja, Roderick James Dillon, Fernando Ariel Genta*

*** Correspondence:** Corresponding Author: genta@ioc.fiocruz.br or gentafernando@gmail.com

A

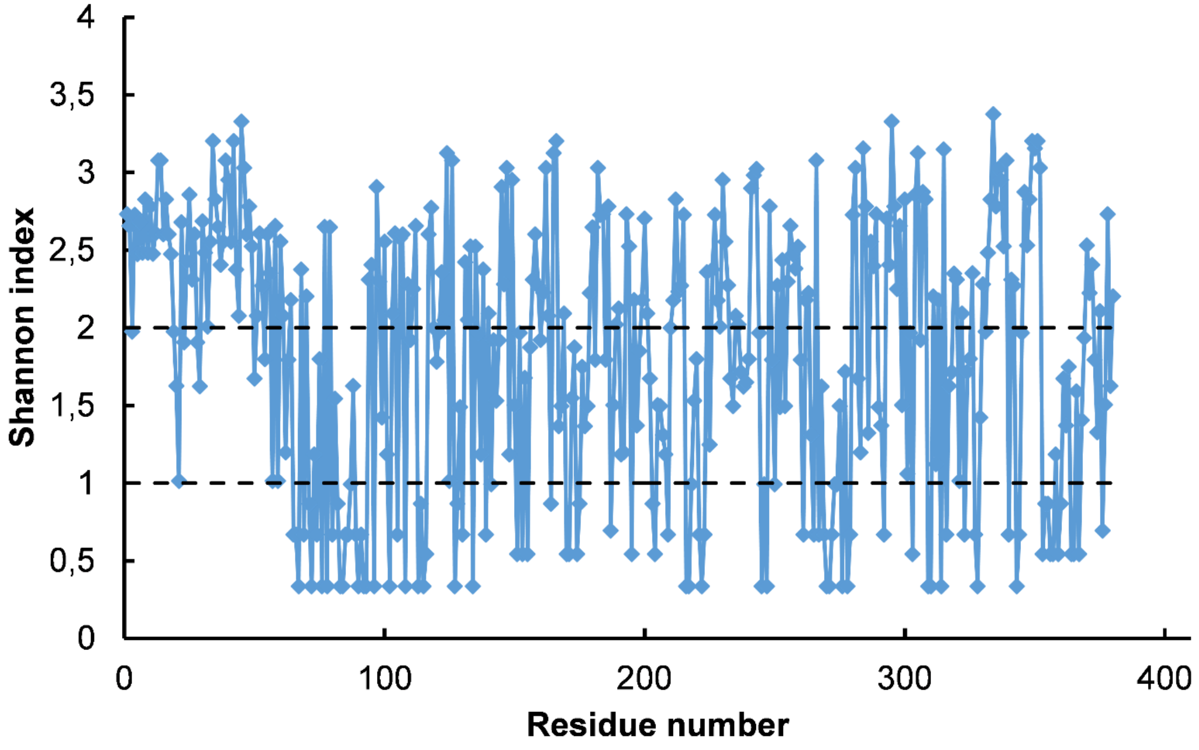
Shannon index
0
0,5
1
1,5
2
2,5
3
3,5
4
0
100
200
300
400
Residue number

B

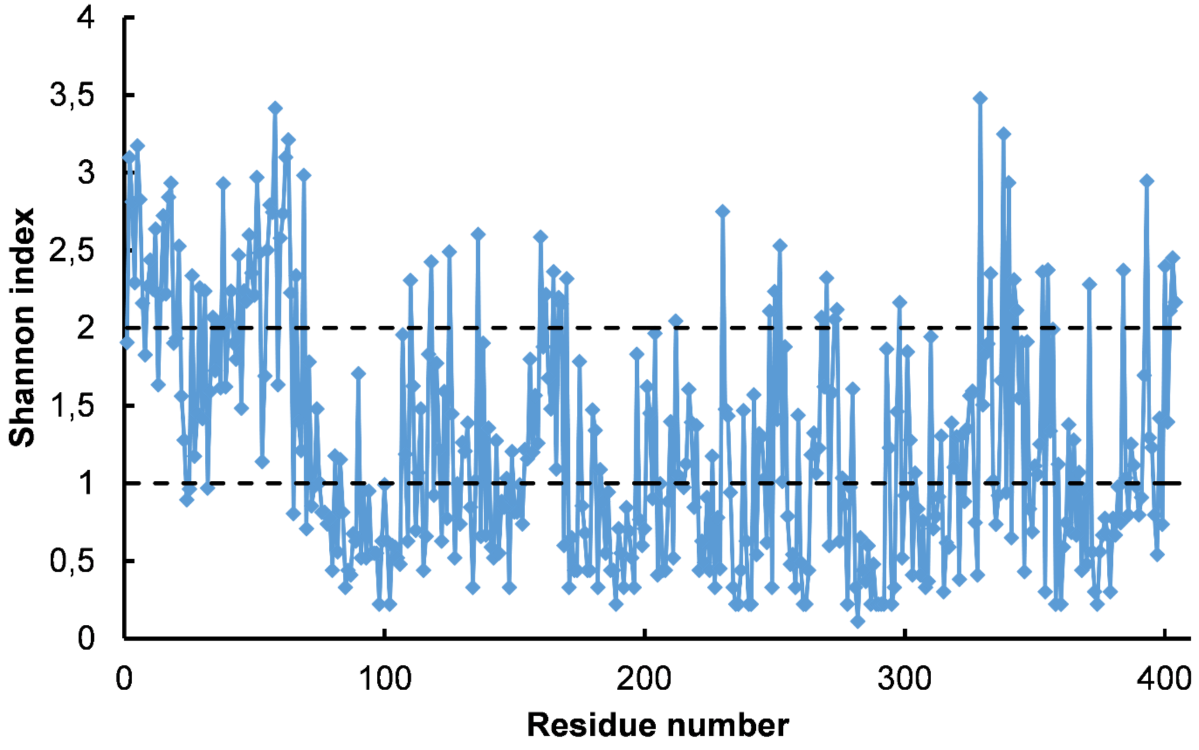
Shannon index
0
0,5
1
1,5
2
2,5
3
3,5
4
0
100
200
300
400
Residue number

Supplementary Figure 10. Profiles of amino acid diversity along sequences of peptidases from family A1. Diversity was estimated as Shannon index in each position of multiple alignments of A1 peptidases from (A) *Rhodnius prolixus* and (B) selected vertebrate species. See Materials and Methods for details.
