# Supplementary material for: Genome Wide Mapping of Peptidases in Rhodnius prolixus: Identification of Protease Gene Duplications, Horizontally Transferred Proteases and Analysis of Peptidase A1 Structures, with Considerations on Their Role in the Evolution of Hematophagy in Triatominae
Source: Front Physiol. 2017 Dec 12;8:1051. doi: 10.3389/fphys.2017.01051 (PMC5736985; doi:10.3389/fphys.2017.01051)
Supplement: Supplementary file 11 [file Table1.DOCX]

Supplementary Material

Genome wide mapping of peptidases in *Rhodnius prolixus*: identification of protease gene duplications, horizontally transferred proteases and analysis of peptidase A1 structures, with considerations on their role in the evolution of hematophagy in Triatominae

**Bianca Santos Henriques, Bruno Gomes, Caroline da Silva Moraes, Samara Graciane Costa, Rafael Dias Mesquita, Viv Maureen Dillon, Eloi de Souza Garcia, Patricia Azambuja, Roderick James Dillon, Fernando Ariel Genta***

*** Correspondence:** Corresponding Author: genta@ioc.fiocruz.br or [gentafernando@gmail.com](mailto:gentafernando@gmail.com)

**Supplementary Table 1.**  Oligonucleotide primers used in PCR and RT-PCR reactions (5’-3’) for amplification of fragments of *Rhodnius prolixus* protease coding genes belonging to different families. Peptidase family nomenclature according to the MEROPS database.

| Peptidase Family | Gene | Forward | Reverse |
| --- | --- | --- | --- |
| A1 | RPRC002696-RA | TGCAGAAGCTACCTCAGTACCCG | CATAACGCCACCGACCGAAGT |
|  | RPRC004171-RA | CTTCCGCCAGTAGAGTTCATCATTGG | CATTCCTCGCCTCCCAGTTCCAT |
|  | RPRC008989-RA | GGTTCTCTTCAAGTGGTCAA | TGCGATGTCATTCCTAAC |
|  | RPRC010954-RA | TTCGTGTTTCTGGTCTTCTCTCCCA | GCGTCCCGATAGGCGATGTTTTC |
|  | RPRC012487-RA | GGTTCTCTTCAAGTGGTCAA | TGCGATGTCATTCCTAAC |
|  | RPRC012785-RA | TCTCGGTTTGGCGTTCCCATCAA | TCTCACCTCCAAGCTCATCGTCCA |
|  | RPRC006698-RA | CTGTTTCTCAAGAGGGATACTGG | CAATCAATAATGCCGATTCCACC |
|  | RPRC011752-RA | TATGGAACAGGAGAAGTTGAAGG | GAAATTGCTGGTGACAGGTTTTC |
|  | RPRC012664-RA | GCAGACAAACTCTATTGAACCTG | GGTATCTACGAGGGCTTTACATC |
|  | RPRC012786-RA | ATATTCGGTGGAGTGGATAAAGAG | CAATAAATGAAGTACCAGTGTCAGC |
|  | RPRC015079-RA | CATTAAAAGCAACTGTGGAGGG | ATAGGGTAGAGAATCCGAGCAT |
|  | RPRC002478-RA_RPRC002479 | GTTCAAGGATTTGATGTTGCCA | CGCCATTTTCTGGATCATTCTC |
|  | RPRC004330-RA_RPRC010954 | GAACCTGGGAATGCCTTTTTAC | CAAATCTCCGTTAGGATCTCTGT |
|  | RPRC006028-RA _RPRC006290 | CATTTGACTATTGGAGGCATCAC | CTGAACCACTCCTTGCTTATAC |
|  | RPRC006759-RA | CATCTGTGTTATGCCCTGTTATAC | TCCTTTTAGCTCTAGTGAACCAAG |
|  | RPRC012508-RA_RPRC012513 | AATTCTAGTCATGTGCTTAAT | AGAACACCATTTGCTGCAAT |
|  | RPRC014747-RA_RPRC012504 | AGGAGAAGTTGAAGGACACTATTC | AGGGTAAGCTAGTCCAAATAAACC |
|  | RPRC015076-RA | TGCTGTCTCCGATGTTAGGATTC | CGGCTTCTTGTACTCCACTTTTC |
|  | RPRC015082-RA | TTCTATCTACGGACACTCTGACT | CGAATTTAGCTTGTACGAACGG |
| C2 | RPRC002326-RA | CCCAGGAAATATGTGATTGTTGG | GCGTAATTCTCTTCAAAGGACTG |
|  | RPRC012930-RA | ATCACAAGGCGTACTAGGGA | CCACGTCTATCACAAGGCAA |
|  | RPRC013350-RA | ACAGCGGTTATTGCACTCAT | TATGTACGCAGTTTGCCACA |
|  | RPRC013606-RA | TTTGACGCAGTGGTGGAAAGCCG | ACCAGCCAAAAAGTGTGGGTGTGTC |
|  | RPRC014368-RA | TAAATGGGTGCGACCAGAAG | TAACTGCCAAAGAAGCGACA |
|  | RPRC007632-RA | TGGTGTGGCGAATGGGTGGAAGT | CCTCGTAGGAACCGTGCAATTTAGCG |
|  | RPRC012594-RA | TAGAAAGAGAGAGCATGGTAGGA | CAAGCCAAATGGTTCCAGATAAC |
|  | RPRC013347-RA | TTCTCGGCGGTATCACTATGTCA | CGCACCCATTCTCCTTCAACTATG |
|  | RPRC013353-RA | AAATATGTAATAATCCGAAAT | CAAGTCGATAACGTATAAT |
|  | RPRC013355-RA | GGCGGATGTATAAAGTATTGGGTA | CTTTTGAGAACGTCTATGCTTCTG |
|  | RPRC013605-RA | TACAACGCACTGAAAGCACA | GTTTCCACCTATACGACGGG |
|  | RPRC015123-RA | TTCGCTGGTGGAAAGGAAAT | GTAGAGAACAGGCTCAGTGC |
| M17 | RPRC000644-RA | GTCCAAAACATTGCTCCTGC | CAGTGCCTTTTTCATGCCAG |
|  | RPRC011316-RA | GCTGATACTGACAATGAAGGACGCA | TCCAGAGCCCAAACCCCATCT |
|  | RPRC014324-RA | TCAGCGTTCCTATCCGTTTGGCG | GCTCCCATCCATCCTTGTCACTTTCA |
|  | RPRC014856-RA | GCGACTATGGATTACCTATGCCCCT | ATGGTGGTTCACAAGACCCCTGA |
|  | RPRC000886-RA | GTTAAAGTTAGGGAGTGGGCTAA | CCTTTCCAATGAGTACGATAGGT |
|  | RPRC003574-RA | GTAGTGTGGGAAGAACTGAAGAA | GGAGAAAATCAACATTAGGAGCG |
|  | RPRC008281-RA | TATGATGAGGGTTGCTAGTATGC | ACATCTGAAGAAGGAGTCGTAAG |
|  | RPRC009154-RA | CACTGGTGGTGCTGATATTAAAG | CTGTTACGAACCATACTCATTGC |
|  | RPRC012383-RA | GGTGGTATTGGTTGGTAAAGGTA | CACAAAGTGGTATCAATGCTCTC |
|  | RPRC012689-RA | GACCGTTAGATATGCGTCTACTT | CAAAACAATCAGCTACTCCTGTG |
|  | RPRC012692-RA | GGATAGAGGGCAAGAAGATGAAT | TCCACCAGTGTCGAATGTTATAC |
|  | RPRC013170-RA | TCTGGCTGATGGAGTTGTTTATG | CTTGACAGTTGGTAAGTAATGCG |
|  | RPRC014323-RA | CATCAAGGGTGAAGCACAGA | ACACGGGCATAGCAATTCTT |
|  | RPRC000644-RA | GTCCAAAACATTGCTCCTGC | CAGTGCCTTTTTCATGCCAG |
| M74 | RPRC003168 | GTTAATGCCGAAAATTGGCGATG | CAAAGAAACCGTCATAGAACCCG |
| S24 | RPRC002798 | AAAGCCCCATTGTTGTCGTTTT | AATTTCGCCCACTCTTACAGGA |
|  | RPRC005865 | CAATCGCTCACTGGCTTAATGG | TTTCATCCCGCTTAGTCTCCTG |
|  | RPRC010630 | CCTCAAGAATACGCTGTTGGC | AAACTCCCCGTGTTGTATCCG |
| S29 | RPRC004810 | TTATTGGAAGGAGGGTGGTGTG | CCTTTTTCTTCGGCAACTCTGG |
|  | RPRC013821 | TATCTCCGTGTCATCTTGTGGTC | CACTGTTTGTCTAGTGCCAATCG |
