# Supplementary material for: Genome Wide Mapping of Peptidases in Rhodnius prolixus: Identification of Protease Gene Duplications, Horizontally Transferred Proteases and Analysis of Peptidase A1 Structures, with Considerations on Their Role in the Evolution of Hematophagy in Triatominae
Source: Front Physiol. 2017 Dec 12;8:1051. doi: 10.3389/fphys.2017.01051 (PMC5736985; doi:10.3389/fphys.2017.01051)
Supplement: Supplementary file 12 [file Table2.DOCX]

Supplementary Material

Genome wide mapping of peptidases in *Rhodnius prolixus*: identification of protease gene duplications, horizontally transferred proteases and analysis of peptidase A1 structures, with considerations on their role in the evolution of hematophagy in Triatominae

**Bianca Santos Henriques, Bruno Gomes, Caroline da Silva Moraes, Samara Graciane Costa, Rafael Dias Mesquita, Viv Maureen Dillon, Eloi de Souza Garcia, Patricia Azambuja, Roderick James Dillon, Fernando Ariel Genta***

*** Correspondence:** Corresponding Author: genta@ioc.fiocruz.br or [gentafernando@gmail.com](mailto:gentafernando@gmail.com)

**Supplementary Table 2.**  Peptidases in *Rhodnius prolixus* genome. Frequency of coding genes by peptidase class and family in *R. prolixus*.

| Peptidase class | Number of families | Families | Number of genes | Number of genes |
| --- | --- | --- | --- | --- |
| Aspartic | 4 | A01 | 27 | 23 |
|  |  | A02 |  | 1 |
|  |  | A22 |  | 1 |
|  |  | A28 |  | 2 |
| Cysteine | 21 | C01 | 92 | 17 |
|  |  | C02 |  | 12 |
|  |  | C12 |  | 3 |
|  |  | C13 |  | 3 |
|  |  | C14 |  | 5 |
|  |  | C15 |  | 1 |
|  |  | C19 |  | 21 |
|  |  | C26 |  | 3 |
|  |  | C40 |  | 1 |
|  |  | C44 |  | 5 |
|  |  | C46 |  | 1 |
|  |  | C48 |  | 4 |
|  |  | C54 |  | 1 |
|  |  | C56 |  | 2 |
|  |  | C64/C85 |  | 4 |
|  |  | C65 |  | 1 |
|  |  | C67 |  | 1 |
|  |  | C69 |  | 1 |
|  |  | C78 |  | 2 |
|  |  | C86 |  | 2 |
|  |  | C97 |  | 2 |
| Metallo | 25 | M01 | 128 | 12 |
|  |  | M02 |  | 6 |
|  |  | M03 |  | 1 |
|  |  | M08 |  | 2 |
|  |  | M10 |  | 6 |
|  |  | M12A |  | 8 |
|  |  | M12B |  | 5 |
|  |  | M13 |  | 9 |
|  |  | M14 |  | 13 |
|  |  | M16 |  | 5 |
|  |  | M17 |  | 14 |
|  |  | M19 |  | 3 |
|  |  | M20 |  | 6 |
|  |  | M22 |  | 2 |
|  |  | M23 |  | 1 |
|  |  | M24 |  | 12 |
|  |  | M28 |  | 3 |
|  |  | M38 |  | 3 |
|  |  | M41 |  | 4 |
|  |  | M48 |  | 2 |
|  |  | M67 |  | 7 |
|  |  | M74 |  | 1 |
|  |  | M76 |  | 1 |
|  |  | M79 |  | 1 |
|  |  | M87 |  | 1 |
| Asparagine | 1 | N06 | 1 | 1 |
| Serine | 16 | S01 | 161 | 94 |
|  |  | S08 |  | 7 |
|  |  | S09 |  | 6 |
|  |  | S10 |  | 6 |
|  |  | S11 |  | 1 |
|  |  | S14 |  | 1 |
|  |  | S16 |  | 2 |
|  |  | S24 |  | 3 |
|  |  | S28 |  | 4 |
|  |  | S29 |  | 2 |
|  |  | S33 |  | 21 |
|  |  | S54 |  | 5 |
|  |  | S59 |  | 2 |
|  |  | S60 |  | 5 |
|  |  | S72 |  | 1 |
|  |  | S81 |  | 1 |
| Threonine | 3 | T01 | 22 | 15 |
|  |  | T02 |  | 4 |
|  |  | T03 |  | 3 |
