# Supplementary material for: Genome Wide Mapping of Peptidases in Rhodnius prolixus: Identification of Protease Gene Duplications, Horizontally Transferred Proteases and Analysis of Peptidase A1 Structures, with Considerations on Their Role in the Evolution of Hematophagy in Triatominae
Source: Front Physiol. 2017 Dec 12;8:1051. doi: 10.3389/fphys.2017.01051 (PMC5736985; doi:10.3389/fphys.2017.01051)
Supplement: Supplementary file 13 [file Table3.DOCX]

Supplementary Material

Genome wide mapping of peptidases in *Rhodnius prolixus*: identification of protease gene duplications, horizontally transferred proteases and analysis of peptidase A1 structures, with considerations on their role in the evolution of hematophagy in Triatominae

**Bianca Santos Henriques, Bruno Gomes, Caroline da Silva Moraes, Samara Graciane Costa, Rafael Dias Mesquita, Viv Maureen Dillon, Eloi de Souza Garcia, Patricia Azambuja, Roderick James Dillon, Fernando Ariel Genta***

*** Correspondence:** Corresponding Author: genta@ioc.fiocruz.br or [gentafernando@gmail.com](mailto:gentafernando@gmail.com)

**Supplementary Table 3.**  Results of transmembrane topology and signal peptide predictor of peptidase families A1 and C1 in *Rhodnius prolixus.* Coding gene: Vectorbase code; Sig pep: presence of initial signal peptide; Cyt: presence of cytosolic regions; Non-cyt: presence of non-cytosolic regions; Trans: presence of transmembrane regions. Genes with two identifiers are result of fusion of gene predictions based on transcriptomic data.

| Family | Coding gene | SuperContig | Sig pep | Cyt | Non-cyt | Trans |
| --- | --- | --- | --- | --- | --- | --- |
| A1 | **RPRC002479-2478** | KQ035965 | Yes | - | Yes | - |
|  | RPRC002696 | KQ034126 | - | - | Yes* | - |
|  | RPRC004171 | KQ034610 | Yes | - | Yes | - |
|  | **RPRC004330-10954** | KQ034470/ KQ034470 | Yes | - | Yes | - |
|  | **RPRC006028-6290** | KQ034219 | Yes | - | Yes | - |
|  | RPRC006698 | KQ034534 | Yes | - | Yes | - |
|  | RPRC006759 | KQ034118 | Yes | - | Yes | - |
|  | RPRC008989 | KQ036163 | - | - | Yes* | - |
|  | RPRC011752 | ACPB03043556 | - | - | Yes* | - |
|  | RPRC012487 | ACPB03042715 | - | - | Yes* | - |
|  | **RPRC012504-14747** | KQ036163/ KQ037387 | - | Yes | - | - |
|  | **RPRC012508-12513** | KQ036163 | Yes | - | Yes | - |
|  | RPRC012664 | KQ035425 | Yes | - | Yes | - |
|  | RPRC012785 | KQ034079 | Yes | - | Yes | - |
|  | RPRC012786 | KQ034079 | Yes | - | Yes | - |
|  | RPRC015076 | KQ035270 | Yes | - | Yes | - |
|  | RPRC015079 | KQ035270 | Yes | - | Yes | - |
|  | RPRC015082 | KQ035270 | Yes | - | Yes | - |
| C1 | RPRC000294 | KQ034071 | Yes | - | Yes | - |
|  | RPRC000405 | KQ034071 | Yes | - | Yes | - |
|  | RPRC015289 | KQ034071 | Yes | - | Yes | - |
|  | RPRC000205 | KQ034071 | Yes | - | Yes | - |
|  | RPRC015299 | KQ034071 | - | - | Yes | - |
|  | RPRC015288 | KQ034071 | Yes | - | Yes | - |
|  | RPRC015290 | KQ034071 | Yes | - | Yes | - |
|  | **RPRC006917-6907** | KQ034107 | Yes | - | Yes | - |
|  | RPRC000309 | KQ034071 | Yes | - | Yes | - |
|  | RPRC002593 | KQ034760 | Yes | - | Yes | - |
|  | RPRC005321 | KQ034084 | Yes | - | Yes | - |
|  | RPRC010398 | KQ034282 | Yes | - | Yes | - |
|  | RPRC008250 | KQ034065 | - | - | Yes | - |
|  | RPRC013528 | KQ034100 | - | - | Yes | - |
|  | RPRC002640 | KQ034302 | - | Yes | - | - |
|  | RPRC013182 | KQ034097 | Yes | - | Yes | - |
|  | RPRC005322 | KQ034084 | Yes | - | Yes | - |
