# Supplementary material for: Genome Wide Mapping of Peptidases in Rhodnius prolixus: Identification of Protease Gene Duplications, Horizontally Transferred Proteases and Analysis of Peptidase A1 Structures, with Considerations on Their Role in the Evolution of Hematophagy in Triatominae
Source: Front Physiol. 2017 Dec 12;8:1051. doi: 10.3389/fphys.2017.01051 (PMC5736985; doi:10.3389/fphys.2017.01051)
Supplement: Supplementary file 14 [file Table4.DOCX]

Supplementary Material

Genome wide mapping of peptidases in *Rhodnius prolixus*: identification of protease gene duplications, horizontally transferred proteases and analysis of peptidase A1 structures, with considerations on their role in the evolution of hematophagy in Triatominae

**Bianca Santos Henriques, Bruno Gomes, Caroline da Silva Moraes, Samara Graciane Costa, Rafael Dias Mesquita, Viv Maureen Dillon, Eloi de Souza Garcia, Patricia Azambuja, Roderick James Dillon, Fernando Ariel Genta***

*** Correspondence:** Corresponding Author: genta@ioc.fiocruz.br or [gentafernando@gmail.com](mailto:gentafernando@gmail.com)

| **Species name** | | **Common name** | | **Feeding habit** | | **Class: Order** |  |
| --- | --- | --- | --- | --- | --- | --- | --- |
|  |  |  |  | **larvae** | **adult** |  |  |
| *Acyrthosiphon pisum* | | pea aphid | | phytophagous | | Hexapoda: Hemiptera |  |
| *Ixodes scapularis* | | deer tick/ blacklegged tick | | hematophagous | | Chelicerata: Ixodida |  |
| *Daphnia pulex* | | water flea | | detritivore | | Crustacea: Cladocera |  |
| *Tribolium castaneum* | | red flour beetle | | omnivore | | Hexapoda: Coleoptera |  |
| *Aedes aegypti* | | yellow fever mosquito | | detritivore | phytophagous  hematophagous | Hexapoda: Diptera |  |
| *Anopheles gambiae* | | mosquito | | detritivore | phytophagous  hematophagous | Hexapoda: Diptera |  |
| *Culex quinquefasciatus* | | southern house mosquito | | detritivore | phytophagous  hematophagous | Hexapoda: Diptera |  |
| *Drosophila melanogaster* | | fruit fly/vinegar fly | | detritivore | phytophagous | Hexapoda: Diptera |  |
| *Drosophila pseudoobscura* | | fruit fly | | detritivore | phytophagous | Hexapoda: Diptera |  |
| *Glossina morsitans* | | Tsetse fly | | hematophagous | | Hexapoda: Diptera |  |
| *Apis mellifera* | | European honey bee | | phytophagous | | Hexapoda: Hymenoptera |  |
| *Acromyrmex echinatior* | | leafcutter ant | | fungivore | phytophagous | Hexapoda: Hymenoptera |  |
| *Camponotus floridanus* | | Florida carpenter ant | | carnivore | | Hexapoda: Hymenoptera |  |
| *Harpegnathos saltator* | | Indian jumping ant | | carnivore | | Hexapoda: Hymenoptera |  |
| *Solenopsis* *invicta* | | fire ant | | phytophagous | | Hexapoda: Hymenoptera |  |
| *Nasonia vitripennis* | | parasitoid wasp | | carnivore | | Hexapoda: Hymenoptera |  |
| *Danaus plexippus* | | monarch butterfly | | phytophagous | | Hexapoda: Lepidoptera |  |
| *Pediculus humanus* | | Body louse | | hematophagous | | Hexapoda: Phthiraptera |  |
|  | |  | |  | | |  |

**Supplementary Table 4.**  Description and characteristics of 18 arthropod species with genomic information available in the MEROPS database.
