# Supplementary material for: Genome Wide Mapping of Peptidases in Rhodnius prolixus: Identification of Protease Gene Duplications, Horizontally Transferred Proteases and Analysis of Peptidase A1 Structures, with Considerations on Their Role in the Evolution of Hematophagy in Triatominae
Source: Front Physiol. 2017 Dec 12;8:1051. doi: 10.3389/fphys.2017.01051 (PMC5736985; doi:10.3389/fphys.2017.01051)
Supplement: Supplementary file 15 [file Table5.DOCX]

Supplementary Material

Genome wide mapping of peptidases in *Rhodnius prolixus*: identification of protease gene duplications, horizontally transferred proteases and analysis of peptidase A1 structures, with considerations on their role in the evolution of hematophagy in Triatominae

**Bianca Santos Henriques, Bruno Gomes, Caroline da Silva Moraes, Samara Graciane Costa, Rafael Dias Mesquita, Viv Maureen Dillon, Eloi de Souza Garcia, Patricia Azambuja, Roderick James Dillon, Fernando Ariel Genta***

*** Correspondence:** Corresponding Author: genta@ioc.fiocruz.br or [gentafernando@gmail.com](mailto:gentafernando@gmail.com)

**Supplementary Table 5.**  Frequency of coding genes in each peptidase family by arthropod species. Rho.pro, Acy.pis, Ixo.sca, Dap.pul, Tri.cas, Aed.aeg, Anp.gam, Cul.qui, Dro.mel, Dro.pse, Glo.mor, Api.mel, Acr.ech, Cam.flo, Har.sal, Sol.inv, Nas.vit, Dan.ple and Ped.hum are the respective frequency of coding genes in *Rhodnius prolixus,* *Acyrthosiphon pisum*, *Ixodes scapularis*, *Daphnia. pulex*, *Tribolium castaneum*, *Aedes aegypti*, *Anopheles gambiae*, *Culex quinquefasciatus*, *Drosophila melanogaster*, *Drosophila pseudoobscura*, *Glossina morsitans*, *Apis mellifera*, *Acromyrmex echinatior*, *Camponotus floridanus*, *Harpegnathos saltator*, *Solenopsis* *invicta*, *Nasonia vitripennis*, *Danaus plexippus* and *Pediculus humanus.* Per 90: Percentil 90% of number of coding genes distribution among 19 arthropod species.

| Family | *Rho.*  *pro* | *Acy.*  *pis* | *Ixo.*  *sca* | *Dap.*  *pul* | *Tri.*  *cas* | *Aed.*  *aeg* | *Anp.*  *gam* | *Cul.*  *qui* | *Dro.*  *mel* | *Dro.*  *pse* | *Glo.*  *mor* | *Api.*  *mel* | *Acr.*  *ech* | *Cam.*  *flo* | *Har.*  *sal* | *Sol.*  *inv* | *Nas.*  *vit* | *Dan.*  *ple* | *Ped.*  *hum* | Per  90 |
| --- | --- | --- | --- | --- | --- | --- | --- | --- | --- | --- | --- | --- | --- | --- | --- | --- | --- | --- | --- | --- |
| A01 | 23 | 2 | 5 | 3 | 2 | 1 | 1 | 1 | 13 | 14 | 3 | 1 | 17 | 89 | 1 | 3 | 2 | 2 | 2 | 18.2 |
| A02 | 1 | 10 | 0 | 0 | 0 | 0 | 3 | 0 | 26 | 9 | 3 | 0 | 0 | 1 | 0 | 2 | 1 | 0 | 0 | 9.2 |
| A22 | 1 | 3 | 14 | 4 | 3 | 3 | 3 | 4 | 3 | 3 | 3 | 3 | 3 | 3 | 3 | 3 | 4 | 3 | 3 | 4 |
| A28 | 2 | 46 | 1 | 23 | 21 | 1 | 1 | 1 | 3 | 6 | 0 | 1 | 1 | 5 | 1 | 6 | 14 | 2 | 0 | 21.4 |
| C01 | 17 | 39 | 21 | 46 | 29 | 14 | 11 | 19 | 14 | 12 | 8 | 7 | 9 | 8 | 7 | 9 | 9 | 20 | 7 | 31 |
| C02 | 12 | 8 | 9 | 6 | 8 | 9 | 9 | 7 | 4 | 3 | 3 | 7 | 6 | 5 | 5 | 6 | 4 | 5 | 8 | 9 |
| C12 | 3 | 5 | 4 | 3 | 4 | 3 | 4 | 3 | 4 | 4 | 4 | 4 | 3 | 3 | 3 | 3 | 3 | 4 | 3 | 4 |
| C13 | 3 | 6 | 5 | 2 | 1 | 1 | 1 | 1 | 1 | 1 | 1 | 1 | 1 | 1 | 1 | 1 | 1 | 1 | 1 | 3.4 |
| C14 | 5 | 6 | 8 | 31 | 9 | 11 | 14 | 13 | 7 | 7 | 6 | 6 | 9 | 8 | 5 | 14 | 11 | 4 | 4 | 14 |
| C15 | 1 | 1 | 1 | 1 | 1 | 1 | 1 | 1 | 1 | 1 | 1 | 1 | 1 | 1 | 1 | 1 | 1 | 1 | 1 | 1 |
| C19 | 21 | 41 | 34 | 34 | 30 | 23 | 25 | 29 | 23 | 19 | 24 | 32 | 34 | 40 | 23 | 33 | 31 | 22 | 23 | 35.2 |
| C26 | 3 | 7 | 5 | 11 | 9 | 8 | 3 | 6 | 8 | 12 | 13 | 19 | 11 | 7 | 10 | 7 | 3 | 6 | 8 | 12.2 |
| C40 | 1 | 3 | 0 | 0 | 0 | 0 | 0 | 0 | 0 | 0 | 1 | 0 | 0 | 0 | 0 | 0 | 0 | 0 | 0 | 1 |
| C44 | 5 | 4 | 3 | 2 | 5 | 4 | 4 | 5 | 6 | 12 | 7 | 10 | 7 | 10 | 12 | 5 | 5 | 9 | 12 | 12 |
| C46 | 1 | 1 | 1 | 2 | 3 | 2 | 1 | 2 | 2 | 3 | 3 | 4 | 3 | 2 | 3 | 2 | 1 | 2 | 3 | 3 |
| C48 | 4 | 42 | 9 | 8 | 5 | 3 | 3 | 3 | 7 | 6 | 9 | 5 | 11 | 4 | 3 | 6 | 9 | 3 | 4 | 9.4 |
| C54 | 1 | 1 | 2 | 2 | 2 | 2 | 2 | 2 | 2 | 2 | 2 | 2 | 2 | 4 | 2 | 2 | 1 | 2 | 1 | 2 |
| C56 | 2 | 4 | 3 | 4 | 6 | 4 | 2 | 3 | 4 | 5 | 9 | 12 | 7 | 5 | 5 | 9 | 3 | 4 | 5 | 9 |
| C64/C85 | 4 | 6 | 4 | 7 | 5 | 5 | 6 | 5 | 7 | 6 | 4 | 6 | 7 | 7 | 5 | 7 | 6 | 5 | 5 | 7 |
| C65 | 1 | 1 | 1 | 1 | 1 | 1 | 1 | 1 | 1 | 1 | 1 | 1 | 1 | 1 | 1 | 3 | 1 | 1 | 1 | 1 |
| C67 | 1 | 0 | 0 | 1 | 1 | 1 | 1 | 1 | 1 | 1 | 1 | 1 | 1 | 1 | 1 | 1 | 1 | 1 | 1 | 1 |
| C69 | 1 | 0 | 2 | 0 | 2 | 1 | 1 | 1 | 1 | 2 | 1 | 1 | 0 | 0 | 0 | 0 | 0 | 1 | 2 | 2 |
| C78 | 2 | 2 | 1 | 3 | 2 | 3 | 3 | 3 | 2 | 2 | 2 | 3 | 2 | 1 | 2 | 2 | 1 | 2 | 1 | 3 |
| C86 | 2 | 1 | 2 | 3 | 2 | 1 | 1 | 1 | 1 | 2 | 1 | 2 | 2 | 2 | 2 | 2 | 2 | 1 | 2 | 2 |
| C97 | 2 | 2 | 2 | 2 | 4 | 2 | 1 | 2 | 2 | 1 | 1 | 2 | 4 | 3 | 2 | 2 | 2 | 2 | 2 | 3.2 |
| M01 | 12 | 45 | 20 | 24 | 20 | 28 | 25 | 24 | 22 | 20 | 14 | 14 | 40 | 58 | 30 | 40 | 20 | 16 | 10 | 41 |
| M02 | 6 | 10 | 7 | 9 | 6 | 8 | 11 | 8 | 6 | 7 | 5 | 4 | 5 | 6 | 5 | 7 | 3 | 9 | 6 | 9.2 |
| M03 | 1 | 3 | 5 | 3 | 3 | 2 | 2 | 2 | 2 | 2 | 2 | 4 | 2 | 2 | 2 | 3 | 3 | 2 | 2 | 3.2 |
| M08 | 2 | 1 | 2 | 1 | 1 | 1 | 1 | 1 | 1 | 1 | 1 | 1 | 1 | 1 | 1 | 3 | 1 | 1 | 1 | 2 |
| M10 | 6 | 3 | 6 | 11 | 9 | 8 | 3 | 9 | 2 | 2 | 3 | 3 | 7 | 11 | 2 | 7 | 3 | 3 | 2 | 9.4 |
| M12A | 8 | 6 | 7 | 31 | 3 | 23 | 15 | 25 | 13 | 12 | 6 | 3 | 6 | 6 | 3 | 10 | 10 | 10 | 4 | 23.4 |
| M12B | 5 | 11 | 36 | 13 | 12 | 9 | 13 | 11 | 11 | 8 | 11 | 16 | 13 | 12 | 11 | 16 | 22 | 9 | 11 | 17.2 |
| M13 | 9 | 14 | 221 | 26 | 23 | 9 | 7 | 9 | 30 | 25 | 24 | 15 | 11 | 11 | 15 | 41 | 28 | 7 | 8 | 32.2 |
| M14 | 13 | 16 | 19 | 36 | 17 | 26 | 35 | 30 | 29 | 27 | 27 | 18 | 29 | 18 | 13 | 22 | 22 | 34 | 12 | 34.2 |
| M16 | 5 | 11 | 7 | 7 | 9 | 10 | 12 | 12 | 11 | 16 | 13 | 11 | 47 | 9 | 9 | 10 | 12 | 9 | 11 | 13.6 |
| M17 | 14 | 3 | 5 | 3 | 5 | 5 | 5 | 6 | 9 | 13 | 11 | 4 | 3 | 2 | 2 | 4 | 1 | 4 | 4 | 11.4 |
| M19 | 3 | 1 | 0 | 6 | 3 | 5 | 6 | 4 | 4 | 4 | 4 | 5 | 13 | 9 | 14 | 8 | 7 | 3 | 3 | 9.8 |
| M20 | 6 | 16 | 23 | 3 | 6 | 3 | 2 | 3 | 7 | 10 | 5 | 3 | 3 | 2 | 2 | 2 | 6 | 2 | 1 | 11.2 |
| M23 | 1 | 0 | 0 | 1 | 0 | 0 | 1 | 0 | 0 | 0 | 0 | 1 | 0 | 0 | 0 | 0 | 0 | 0 | 0 | 1 |
| M24 | 12 | 12 | 11 | 10 | 10 | 10 | 12 | 7 | 11 | 6 | 11 | 8 | 10 | 9 | 8 | 17 | 12 | 6 | 9 | 12 |
| M28 | 3 | 6 | 24 | 6 | 5 | 3 | 11 | 5 | 15 | 0 | 9 | 5 | 4 | 6 | 3 | 5 | 4 | 1 | 2 | 11.8 |
| M38 | 3 | 4 | 10 | 2 | 3 | 2 | 2 | 2 | 3 | 0 | 4 | 8 | 7 | 19 | 19 | 10 | 6 | 0 | 0 | 11.8 |
| M41 | 4 | 3 | 4 | 3 | 3 | 3 | 3 | 3 | 3 | 3 | 4 | 3 | 3 | 3 | 3 | 3 | 3 | 3 | 5 | 4 |
| M48 | 2 | 5 | 4 | 1 | 2 | 1 | 1 | 1 | 4 | 4 | 1 | 3 | 2 | 2 | 1 | 2 | 5 | 1 | 1 | 4.2 |
| M67 | 7 | 10 | 12 | 7 | 11 | 7 | 8 | 5 | 8 | 5 | 9 | 9 | 10 | 9 | 13 | 9 | 10 | 7 | 5 | 11.2 |
| M74 | 1 | 0 | 0 | 0 | 0 | 0 | 0 | 0 | 0 | 0 | 0 | 0 | 0 | 0 | 0 | 0 | 0 | 0 | 0 | 0 |
| M76 | 1 | 1 | 1 | 1 | 1 | 1 | 1 | 1 | 1 | 1 | 1 | 0 | 1 | 1 | 1 | 1 | 1 | 1 | 1 | 1 |
| M79 | 1 | 1 | 2 | 2 | 1 | 1 | 1 | 1 | 1 | 1 | 1 | 1 | 1 | 1 | 1 | 1 | 1 | 1 | 1 | 1.2 |
| M87 | 1 | 1 | 11 | 12 | 2 | 1 | 1 | 2 | 0 | 0 | 0 | 0 | 0 | 0 | 0 | 0 | 0 | 0 | 1 | 3.8 |
| N06 | 1 | 0 | 0 | 1 | 0 | 0 | 0 | 0 | 0 | 0 | 0 | 0 | 0 | 0 | 0 | 0 | 0 | 0 | 0 | 0.2 |
| S01 | 94 | 160 | 170 | 251 | 212 | 370 | 479 | 425 | 295 | 216 | 209 | 72 | 94 | 88 | 195 | 153 | 254 | 296 | 258 | 381 |
| S08 | 7 | 11 | 8 | 18 | 25 | 13 | 5 | 11 | 12 | 17 | 17 | 24 | 21 | 21 | 25 | 12 | 5 | 12 | 18 | 24.2 |
| S09 | 6 | 70 | 112 | 118 | 191 | 127 | 60 | 74 | 110 | 123 | 162 | 211 | 154 | 167 | 174 | 145 | 65 | 77 | 87 | 177.4 |
| S10 | 6 | 6 | 30 | 30 | 37 | 35 | 34 | 39 | 39 | 45 | 43 | 46 | 41 | 46 | 48 | 42 | 13 | 20 | 24 | 46 |
| S11 | 1 | 0 | 0 | 1 | 1 | 1 | 0 | 0 | 0 | 0 | 0 | 0 | 0 | 0 | 0 | 0 | 0 | 0 | 0 | 1 |
| S14 | 1 | 2 | 2 | 3 | 4 | 4 | 4 | 5 | 6 | 7 | 10 | 13 | 7 | 8 | 9 | 8 | 1 | 2 | 3 | 9.2 |
| S16 | 2 | 3 | 3 | 6 | 7 | 7 | 4 | 5 | 5 | 6 | 8 | 9 | 6 | 6 | 7 | 6 | 1 | 2 | 3 | 7.2 |
| S24 | 3 | 3 | 0 | 0 | 0 | 0 | 0 | 0 | 0 | 0 | 0 | 1 | 0 | 0 | 0 | 0 | 0 | 0 | 0 | 1.4 |
| S28 | 4 | 9 | 10 | 12 | 15 | 20 | 19 | 28 | 26 | 32 | 28 | 31 | 29 | 29 | 33 | 32 | 4 | 9 | 11 | 32 |
| S29 | 2 | 0 | 0 | 0 | 0 | 0 | 0 | 0 | 0 | 0 | 0 | 0 | 0 | 0 | 0 | 0 | 0 | 0 | 0 | 0 |
| S33 | 21 | 39 | 34 | 36 | 82 | 47 | 58 | 69 | 83 | 92 | 96 | 123 | 117 | 127 | 127 | 154 | 55 | 55 | 59 | 127 |
| S54 | 5 | 6 | 10 | 14 | 21 | 20 | 15 | 21 | 27 | 34 | 34 | 42 | 41 | 33 | 36 | 35 | 6 | 11 | 17 | 37 |
| S59 | 2 | 1 | 1 | 4 | 5 | 5 | 2 | 3 | 4 | 5 | 5 | 6 | 5 | 5 | 6 | 5 | 2 | 3 | 4 | 5.2 |
| S60 | 5 | 5 | 4 | 8 | 15 | 14 | 10 | 19 | 24 | 30 | 29 | 34 | 35 | 32 | 40 | 31 | 8 | 13 | 17 | 34.2 |
| S72 | 1 | 2 | 1 | 2 | 2 | 3 | 4 | 6 | 8 | 14 | 10 | 11 | 10 | 6 | 8 | 5 | 2 | 4 | 6 | 10.2 |
| S81 | 1 | 3 | 1 | 3 | 3 | 3 | 2 | 4 | 8 | 13 | 9 | 10 | 13 | 9 | 10 | 6 | 1 | 5 | 8 | 10.6 |
| T01 | 15 | 16 | 21 | 35 | 36 | 35 | 14 | 18 | 26 | 61 | 45 | 60 | 44 | 16 | 30 | 20 | 15 | 29 | 43 | 48 |
| T02 | 4 | 3 | 5 | 9 | 13 | 9 | 3 | 5 | 5 | 9 | 11 | 15 | 10 | 5 | 5 | 5 | 4 | 7 | 11 | 11.4 |
| T03 | 3 | 13 | 25 | 33 | 30 | 32 | 3 | 7 | 6 | 10 | 12 | 16 | 9 | 6 | 9 | 6 | 8 | 12 | 15 | 30.4 |
