# Supplementary material for: Genome Wide Mapping of Peptidases in Rhodnius prolixus: Identification of Protease Gene Duplications, Horizontally Transferred Proteases and Analysis of Peptidase A1 Structures, with Considerations on Their Role in the Evolution of Hematophagy in Triatominae
Source: Front Physiol. 2017 Dec 12;8:1051. doi: 10.3389/fphys.2017.01051 (PMC5736985; doi:10.3389/fphys.2017.01051)
Supplement: Supplementary file 16 [file Table6.DOCX]

Supplementary Material

Genome wide mapping of peptidases in *Rhodnius prolixus*: identification of protease gene duplications, horizontally transferred proteases and analysis of peptidase A1 structures, with considerations on their role in the evolution of hematophagy in Triatominae

**Bianca Santos Henriques, Bruno Gomes, Caroline da Silva Moraes, Samara Graciane Costa, Rafael Dias Mesquita, Viv Maureen Dillon, Eloi de Souza Garcia, Patricia Azambuja, Roderick James Dillon, Fernando Ariel Genta***

*** Correspondence:** Corresponding Author: genta@ioc.fiocruz.br or [gentafernando@gmail.com](mailto:gentafernando@gmail.com)

**Supplementary Table 6.**  Test to verify similarity/differences of number of peptidases among groups defined by ability to feed in blood. Gene numbers from hematophagous or non-hematophagous species were grouped in two sets and compared using Mann-Whitney test. The test was used to verify if hematophagous and non-hematophagous species present similar distribution in the number of peptidases in each family. In bold: Significant P-value that reject hypothesis tested (i.e. indicate significant differences between/among groups).

|  | Hematophagy | | | | | |
| --- | --- | --- | --- | --- | --- | --- |
| Family | Mann-Whitney U | Wilcoxon W | Z | Asymp. Sig.  (2-tailed) | Exact Sig.  [2*(1-tailed Sig.)] | |
| A01 | 33 | 61 | -0,775757 | 0,437893 | .482b | |
| A02 | 37 | 65 | -0,458113 | 0,646871 | | .711b |
| A22 | 41 | 119 | -0,109349 | 0,912926 | | .967b |
| **A28** | **9,5** | **37,5** | **-2,820713** | **0,004792** | | **.004b** |
| C01 | 42 | 120 | 0 | 1 | | 1.000b |
| **C02** | **15** | **93** | **-2,301174** | **0,021382** | | **.022b** |
| C12 | 37,5 | 65,5 | -0,430192 | 0,667056 | | .711b |
| C13 | 37 | 115 | -0,59244 | 0,553556 | | .711b |
| C14 | 41,5 | 69,5 | -0,042463 | 0,96613 | | .967b |
| C15 | 42 | 120 | 0 | 1 | | 1.000b |
| C19 | 28 | 56 | -1,190549 | 0,233831 | | .261b |
| C26 | 25,5 | 53,5 | -1,403147 | 0,160573 | | .167b |
| C40 | 34,5 | 112,5 | -0,998949 | 0,317819 | | .536b |
| C44 | 28,5 | 56,5 | -1,156274 | 0,247569 | | .261b |
| C46 | 30 | 58 | -1,067485 | 0,285753 | | .340b |
| C48 | 27 | 55 | -1,284749 | 0,19888 | | .227b |
| C54 | 34,5 | 62,5 | -0,823754 | 0,410079 | | .536b |
| C56 | 21 | 49 | -1,803529 | 0,071305 | | .083b |
| C64C85 | 9,5 | 37,5 | -2,847174 | 0,004411 | | .004b |
| C65 | 38,5 | 66,5 | -0,763763 | 0,445009 | | .773b |
| C67 | 39,5 | 67,5 | -0,396942 | 0,69141 | | .837b |
| C69 | 19,5 | 97,5 | -2,040579 | 0,041293 | | .056b |
| C78 | 37 | 115 | -0,464133 | 0,642552 | | .711b |
| C86 | 27 | 55 | -1,456206 | 0,145336 | | .227b |
| C97 | 26 | 54 | -1,644301 | 0,100114 | | .196b |
| M01 | 24,5 | 52,5 | -1,487526 | 0,136876 | | .142b |
| M02 | 32 | 110 | -0,856499 | 0,391722 | | .432b |
| M03 | 27 | 55 | -1,426785 | 0,153642 | | .227b |
| M08 | 34,5 | 112,5 | -0,998949 | 0,317819 | | .536b |
| M10 | 40 | 118 | -0,172785 | 0,86282 | | .902b |
| M12A | 31,5 | 109,5 | -0,894502 | 0,371053 | | .384b |
| M12B | 32 | 60 | -0,861167 | 0,389146 | | .432b |
| M13 | 23,5 | 51,5 | -1,568358 | 0,116798 | | .120b |
| M14 | 41 | 69 | -0,084701 | 0,932499 | | .967b |
| M16 | 41,5 | 119,5 | -0,042748 | 0,965902 | | .967b |
| **M17** | **15** | **93** | **-2,307357** | **0,021035** | | **.022b** |
| M19 | 24 | 52 | -1,536173 | 0,124496 | | .142b |
| M20 | 40 | 68 | -0,17239 | 0,863131 | | .902b |
| M23 | 37 | 115 | -0,597614 | 0,550097 | | .711b |
| M24 | 34 | 112 | -0,684275 | 0,493801 | | .536b |
| M28 | 36,5 | 114,5 | -0,468758 | 0,639243 | | .650b |
| M38 | 27,5 | 55,5 | -1,236919 | 0,216117 | | .227b |
| **M41** | **18** | **96** | **-2,853569** | **0,004323** | | **.045b** |
| M48 | 21,5 | 49,5 | -1,821851 | 0,068478 | | .083b |
| M67 | 25 | 53 | -1,455393 | 0,14556 | | .167b |
| M74 | 36 | 114 | -1,309307 | 0,19043 | | .650b |
| M76 | 38,5 | 116,5 | -0,763763 | 0,445009 | | .773b |
| M79 | 39,5 | 117,5 | -0,396942 | 0,69141 | | .837b |
| M87 | 20 | 98 | -2,03253 | 0,0421 | | .068b |
| N06 | 39,5 | 117,5 | -0,396942 | 0,69141 | | .837b |
| S01 | 24,5 | 102,5 | -1,479669 | 0,138962 | | .142b |
| S08 | 21 | 49 | -1,78266 | 0,074642 | | .083b |
| S09 | 23 | 51 | -1,605793 | 0,108319 | | .120b |
| S10 | 28,5 | 56,5 | -1,142965 | 0,253053 | | .261b |
| S11 | 37 | 115 | -0,597614 | 0,550097 | | .711b |
| S14 | 31 | 59 | -0,934602 | 0,349994 | | .384b |
| S16 | 34 | 62 | -0,685199 | 0,493218 | | .536b |
| S24 | 41,5 | 69,5 | -0,066597 | 0,946903 | | .967b |
| S28 | 28,5 | 56,5 | -1,143469 | 0,252844 | | .261b |
| S29 | 36 | 114 | -1,309307 | 0,19043 | | .650b |
| S33 | 21 | 49 | -1,776383 | 0,07567 | | .083b |
| S54 | 26 | 54 | -1,35403 | 0,175727 | | .196b |
| S59 | 25 | 53 | -1,48094 | 0,138623 | | .167b |
| S60 | 23,5 | 51,5 | -1,564909 | 0,117604 | | .120b |
| S72 | 31 | 59 | -0,937097 | 0,348709 | | .384b |
| S81 | 22,5 | 50,5 | -1,661218 | 0,09667 | | .100b |
| T01 | 34 | 62 | -0,677015 | 0,498397 | | .536b |
| T02 | 38 | 66 | -0,344467 | 0,730495 | | .773b |
| T03 | 40,5 | 68,5 | -0,127164 | 0,89881 | | .902b |
