# Supplementary material for: Genome Wide Mapping of Peptidases in Rhodnius prolixus: Identification of Protease Gene Duplications, Horizontally Transferred Proteases and Analysis of Peptidase A1 Structures, with Considerations on Their Role in the Evolution of Hematophagy in Triatominae
Source: Front Physiol. 2017 Dec 12;8:1051. doi: 10.3389/fphys.2017.01051 (PMC5736985; doi:10.3389/fphys.2017.01051)
Supplement: Supplementary file 17 [file Table7.DOCX]

Supplementary Material

Genome wide mapping of peptidases in *Rhodnius prolixus*: identification of protease gene duplications, horizontally transferred proteases and analysis of peptidase A1 structures, with considerations on their role in the evolution of hematophagy in Triatominae

**Bianca Santos Henriques, Bruno Gomes, Caroline da Silva Moraes, Samara Graciane Costa, Rafael Dias Mesquita, Viv Maureen Dillon, Eloi de Souza Garcia, Patricia Azambuja, Roderick James Dillon, Fernando Ariel Genta***

*** Correspondence:** Corresponding Author: genta@ioc.fiocruz.br or [gentafernando@gmail.com](mailto:gentafernando@gmail.com)

**Supplementary Table 7.**  Test to verify similarity/differences of number of peptidases among groups defined by taxonomy. Gene numbers from all species of each category were grouped in five sets and compared using Kruskal–Wallis test. The test was used to verify if the five groups defined by Taxonomic order present similar distribution in the number of peptidases in each family. In bold: Significant P-value that reject hypothesis tested (i.e. indicate significant differences between/among groups).

|  |  | Classes | | |
| --- | --- | --- | --- | --- |
| Family |  | Chi-Square | df | Asymp. Sig. |
| A01 |  | 1,948905 | 4 | 0,745156 |
| A02 |  | 7,804639 | 4 | 0,099002 |
| **A22** |  | **10,04185** | **4** | **0,039729** |
| A28 |  | 2,880666 | 4 | 0,57799 |
| **C01** |  | **10,62212** | **4** | **0,031156** |
| C02 |  | 5,009813 | 4 | 0,286292 |
| C12 |  | 3,409091 | 4 | 0,491835 |
| **C13** |  | **17,75172** | **4** | **0,00138** |
| C14 |  | 6,182019 | 4 | 0,185961 |
| C15 |  | 0 | 4 | 1 |
| C19 |  | 7,666519 | 4 | 0,104587 |
| C26 |  | 1,938277 | 4 | 0,74711 |
| **C40** |  | **12,61765** | **4** | **0,013303** |
| C44 |  | 8,494595 | 4 | 0,075051 |
| C46 |  | 6,425656 | 4 | 0,169535 |
| C48 |  | 4,128378 | 4 | 0,38891 |
| C54 |  | 7,82 | 4 | 0,098399 |
| C56 |  | 6,074728 | 4 | 0,193637 |
| C64C85 |  | 4,579171 | 4 | 0,333263 |
| C65 |  | 2,166667 | 4 | 0,705136 |
| C67 |  | 7,941176 | 4 | 0,093757 |
| **C69** |  | **9,995455** | **4** | **0,040504** |
| C78 |  | 3,928571 | 4 | 0,415759 |
| **C86** |  | **10,5** | **4** | **0,032797** |
| C97 |  | 7,476654 | 4 | 0,112743 |
| M01 |  | 4,641083 | 4 | 0,326145 |
| M02 |  | 7,147297 | 4 | 0,128306 |
| M03 |  | 6,953333 | 4 | 0,138375 |
| M08 |  | 4,441176 | 4 | 0,34958 |
| M10 |  | 2,247938 | 4 | 0,690263 |
| M12A |  | 8,449198 | 4 | 0,076442 |
| **M12B** |  | **10,47131** | **4** | **0,033194** |
| M13 |  | 6,252868 | 4 | 0,181043 |
| M14 |  | 7,998238 | 4 | 0,091643 |
| M16 |  | 8,651257 | 4 | 0,070433 |
| **M17** |  | **11,0435** | **4** | **0,026079** |
| **M19** |  | **13,49866** | **4** | **0,00908** |
| M20 |  | 6,410584 | 4 | 0,170512 |
| M23 |  | 2,8 | 4 | 0,591833 |
| M24 |  | 4,586253 | 4 | 0,332442 |
| M28 |  | 5,415254 | 4 | 0,24728 |
| **M38** |  | **12,28954** | **4** | **0,015323** |
| M41 |  | 3,677083 | 4 | 0,451468 |
| M48 |  | 3,907856 | 4 | 0,41862 |
| M67 |  | 6,125563 | 4 | 0,189965 |
| M74 |  | 8,5 | 4 | 0,074887 |
| M76 |  | 2,166667 | 4 | 0,705136 |
| **M79** |  | **18** | **4** | **0,001234** |
| **M87** |  | **11,03774** | **4** | **0,026143** |
| N06 |  | 7,941176 | 4 | 0,093757 |
| **S01** |  | **11,22564** | **4** | **0,024142** |
| S08 |  | 5,392035 | 4 | 0,249384 |
| S09 |  | 6,384211 | 4 | 0,172234 |
| **S10** |  | **10,31514** | **4** | **0,035441** |
| S11 |  | 3,75 | 4 | 0,440896 |
| S14 |  | 9,37367 | 4 | 0,052409 |
| S16 |  | 4,455405 | 4 | 0,347868 |
| **S24** |  | **13,27778** | **4** | **0,009995** |
| **S28** |  | **9,857709** | **4** | **0,042894** |
| S29 |  | 8,5 | 4 | 0,074887 |
| **S33** |  | **12,49956** | **4** | **0,013998** |
| **S54** |  | **10,06728** | **4** | **0,039309** |
| S59 |  | 8,014445 | 4 | 0,09105 |
| **S60** |  | **11,72583** | **4** | **0,019511** |
| S72 |  | 9,47861 | 4 | 0,050189 |
| S81 |  | 6,318182 | 4 | 0,176613 |
| T01 |  | 3,646438 | 4 | 0,455964 |
| T02 |  | 6,32377 | 4 | 0,176238 |
| T03 |  | 7,033981 | 4 | 0,134103 |
