# Supplementary material for: Genome Wide Mapping of Peptidases in Rhodnius prolixus: Identification of Protease Gene Duplications, Horizontally Transferred Proteases and Analysis of Peptidase A1 Structures, with Considerations on Their Role in the Evolution of Hematophagy in Triatominae
Source: Front Physiol. 2017 Dec 12;8:1051. doi: 10.3389/fphys.2017.01051 (PMC5736985; doi:10.3389/fphys.2017.01051)
Supplement: Supplementary file 18 [file Table8.DOCX]

Supplementary Material

Genome wide mapping of peptidases in *Rhodnius prolixus*: identification of protease gene duplications, horizontally transferred proteases and analysis of peptidase A1 structures, with considerations on their role in the evolution of hematophagy in Triatominae

**Bianca Santos Henriques, Bruno Gomes, Caroline da Silva Moraes, Samara Graciane Costa, Rafael Dias Mesquita, Viv Maureen Dillon, Eloi de Souza Garcia, Patricia Azambuja, Roderick James Dillon, Fernando Ariel Genta***

*** Correspondence:** Corresponding Author: genta@ioc.fiocruz.br or [gentafernando@gmail.com](mailto:gentafernando@gmail.com)

**Supplementary Table 8.**  Results of transmembrane topology and signal peptide predictor of peptidase families C2 in *Rhodnius prolixus.* Coding gene: Vectorbase code; Sig pep: presence of initial signal peptide; Cyt: presence of cytosolic regions; Non-cyt: presence of non-cytosolic regions; Trans: presence of transmembrane regions.

| Family | Coding gene | SuperContig | Sig pep | Cyt | Non-cyt | Trans | Start Met |
| --- | --- | --- | --- | --- | --- | --- | --- |
| C2 | RPRC002326 | ACPB03026553 | - | - | Yes | - | - |
|  | RPRC007632 | KQ034058 | - | - | Yes | Yes | - |
|  | RPRC012594 | ACPB03028740 | - | - | Yes | - | - |
|  | RPRC012930 | KQ034358 | - | - | Yes* | - | Yes |
|  | RPRC013347 | KQ034117 | - | Yes | - | - | Yes |
|  | RPRC013350 | KQ034117 | - | - | Yes | - | Yes |
|  | RPRC013353 | KQ034117 | - | Yes* | - | - | Yes |
|  | RPRC013355 | KQ034117 | - | - | Yes* | - | Yes |
|  | RPRC013605 | KQ034117 | - | - | Yes* | - | - |
|  | RPRC013606 | KQ034117 | - | - | Yes | - | - |
|  | RPRC014368 | KQ034516 | - | - | Yes | - | Yes |
|  | RPRC015123 | ACPB03026553 | - | - | Yes | - | - |
