# Supplementary material for: Genome Wide Mapping of Peptidases in Rhodnius prolixus: Identification of Protease Gene Duplications, Horizontally Transferred Proteases and Analysis of Peptidase A1 Structures, with Considerations on Their Role in the Evolution of Hematophagy in Triatominae
Source: Front Physiol. 2017 Dec 12;8:1051. doi: 10.3389/fphys.2017.01051 (PMC5736985; doi:10.3389/fphys.2017.01051)
Supplement: Supplementary file 19 [file Table9.DOCX]

Supplementary Material

Genome wide mapping of peptidases in *Rhodnius prolixus*: identification of protease gene duplications, horizontally transferred proteases and analysis of peptidase A1 structures, with considerations on their role in the evolution of hematophagy in Triatominae

**Bianca Santos Henriques, Bruno Gomes, Caroline da Silva Moraes, Samara Graciane Costa, Rafael Dias Mesquita, Viv Maureen Dillon, Eloi de Souza Garcia, Patricia Azambuja, Roderick James Dillon, Fernando Ariel Genta***

*** Correspondence:** Corresponding Author: genta@ioc.fiocruz.br or [gentafernando@gmail.com](mailto:gentafernando@gmail.com)

**Supplementary Table 9.**  Identities, result of transmembrane topology and signal peptide prediction of peptidases of family M17 in *Rhodnius prolixus.* Coding gene: Vectorbase code; Sig pep: presence of initial signal peptide; Cyt: presence of cytosolic regions; Non-cyt: presence of non-cytosolic regions; Trans: presence of transmembrane regions. The gene with two identifiers is the result of fusion of gene predictions based on transcriptomic data.

| Family | Coding gene | SuperContig | Sig pep | Cyt | Non-cyt | Trans | Start Met |
| --- | --- | --- | --- | --- | --- | --- | --- |
| M17 | RPRC000644 | KQ035508 | - | - | Yes | - | - |
|  | RPRC000886 | KQ034806 | - | - | Yes | - | Yes |
|  | RPRC003574 | KQ034497 | - | Yes | Yes | Yes | Yes |
|  | RPRC008281 | KQ034251 | - | - | Yes | - | - |
|  | RPRC009154 | KQ034418 | - | - | Yes | - | Yes |
|  | RPRC011316 | KQ034110 | - | - | Yes* | - | Yes |
|  | RPRC012383 | KQ034202 | - | - | Yes | - | Yes |
|  | RPRC012689 | KQ035139 | - | - | Yes | - | Yes |
|  | RPRC012692 | KQ035139 | - | Yes | Yes | Yes | - |
|  | RPRC013170 | KQ034097 | - | - | Yes | - | Yes |
|  | **RPRC014323-10866** | KQ034126 | - | - | Yes* | - | Yes |
|  | RPRC014324 | KQ034126 | - | - | Yes* | - | Yes |
|  | RPRC014856 | KQ034163 | - | - | Yes | - | - |
