# Supplementary material for: Genome Wide Mapping of Peptidases in Rhodnius prolixus: Identification of Protease Gene Duplications, Horizontally Transferred Proteases and Analysis of Peptidase A1 Structures, with Considerations on Their Role in the Evolution of Hematophagy in Triatominae
Source: Front Physiol. 2017 Dec 12;8:1051. doi: 10.3389/fphys.2017.01051 (PMC5736985; doi:10.3389/fphys.2017.01051)
Supplement: Supplementary file 20 [file Table10.DOCX]

Supplementary Material

Genome wide mapping of peptidases in *Rhodnius prolixus*: identification of protease gene duplications, horizontally transferred proteases and analysis of peptidase A1 structures, with considerations on their role in the evolution of hematophagy in Triatominae

**Bianca Santos Henriques, Bruno Gomes, Caroline da Silva Moraes, Samara Graciane Costa, Rafael Dias Mesquita, Viv Maureen Dillon, Eloi de Souza Garcia, Patricia Azambuja, Roderick James Dillon, Fernando Ariel Genta***

*** Correspondence:** Corresponding Author: genta@ioc.fiocruz.br or [gentafernando@gmail.com](mailto:gentafernando@gmail.com)

**Supplementary Table 10.**  Identity of genes neighbor to peptidase sequences in the genome of *Rhodnius prolixus* from peptidase families with putative bacterial origin. Family – MEROPS classification; Coding genes – *R. prolixus* peptidase sequences; Neighbor genes - neighbor genes from *R. prolixus* in same contigs than ¨coding genes¨; Residues – number of amino acid residues in neighbor coding sequences; Blast-p results – taxonomic group of sequences with best scores in blast-p search with neighbor sequence as query.

| Family | Coding genes | Neighbor genes | Residues | Blast-p results |
| --- | --- | --- | --- | --- |
| M74 | RPRC003168 | - |  | - |
| N6 | RPRC014779 | RPRC014780 | 447 | Gram-negative |
| S24 | RPRC002798 | RPRC002799 | 366 | Arthopod |
|  |  | RPRC002800 | 82 | - |
|  |  | RPRC002801 | 269 | Arthopod |
|  | RPRC005865 | RPRC005864 | 65 | Bacteria/worm |
|  |  | RPRC005866 | 88 | Arthopod |
|  | RPRC010630 | RPRC010628 | 558 | Gram-negative |
|  |  | RPRC010632 | 528 | Gram-negative |
| S29 | RPRC004810 | RPRC004806 | 59 | Arthopod |
|  |  | RPRC004859 | 118 | Arthopod |
|  | RPRC013821 | RPRC001037 | 409 | Arthopod |
|  |  | RPRC013776 | 97 | Arthopod |
|  |  | RPRC013815 | 395 | Arthopod |
|  |  | RPRC013867 | 491 | Arthopod |
