# Supplementary material for: Genome Wide Mapping of Peptidases in Rhodnius prolixus: Identification of Protease Gene Duplications, Horizontally Transferred Proteases and Analysis of Peptidase A1 Structures, with Considerations on Their Role in the Evolution of Hematophagy in Triatominae
Source: Front Physiol. 2017 Dec 12;8:1051. doi: 10.3389/fphys.2017.01051 (PMC5736985; doi:10.3389/fphys.2017.01051)
Supplement: Supplementary file 21 [file Table11.DOCX]

Supplementary Material

Genome wide mapping of peptidases in *Rhodnius prolixus*: identification of protease gene duplications, horizontally transferred proteases and analysis of peptidase A1 structures, with considerations on their role in the evolution of hematophagy in Triatominae

**Bianca Santos Henriques, Bruno Gomes, Caroline da Silva Moraes, Samara Graciane Costa, Rafael Dias Mesquita, Viv Maureen Dillon, Eloi de Souza Garcia, Patricia Azambuja, Roderick James Dillon, Fernando Ariel Genta***

*** Correspondence:** Corresponding Author: genta@ioc.fiocruz.br or [gentafernando@gmail.com](mailto:gentafernando@gmail.com)

**Supplementary Table 11.**  Positional comparison of conserved residues among A1 peptidases from *Rhodnius prolixus* and their human homologs (Pepsin, Renin and Cathepsin D). Cat – catalitic aspartates; Cys – cysteins; Pro – prolines; Asn – asparagines; Lys – lysines; S4 to S2’ – residues at subsite number 4–2’. Subsite numbering according to Schechter and Berger, 1967. Residues roles, positions and numberings in Pepsin, Renin and Cathepsin D were obtained from Baldwin *et al.*, 1993; Dunn and Hung, 2000; Metcalf and Fusek 1993.

| Gene | Cat | Cat | Cys | Cys | Cys | Cys | Cys | Cys | Cys | Cys | Pro | Pro | Pro | Pro | Asn | Asn | Lys |
| --- | --- | --- | --- | --- | --- | --- | --- | --- | --- | --- | --- | --- | --- | --- | --- | --- | --- |
| Pepsin A | D94 | D277 | D88 | C107 | C112 | - | C268 | C272 | C311 | C344 | P85 | P354 | T355 | E356 | T129 | R245 | E249 |
| renin | D104 | D292 | T98 | C117 | C124 | - | C283 | C287 | C325 | C362 | P95 | P372 | P373 | P374 | N141 | N260 | T264 |
| Cathepsin D | D97 | D295 | C91 | C110 | C117 | C160 | C286 | C290 | C329 | C366 | P88 | P376 | P377 | P378 | N134 | N263 | K267 |
| RPRC006698 | D90 | D278 | E84 | C103 | C110 | - | C269 | C273 | C311 | C348 | P81 | P358 | - | - | N127 | R246 | E250 |
| RPRC012786 | D89 | D277 | E83 | C102 | C107 | - | C268 | C272 | C310 | C347 | P80 | A357 | P358 | - | D124 | R243 | T247 |
| RPRC015079 | D91 | D279 | N85 | C104 | C110 | - | C270 | C274 | C312 | C349 | P82 | N359 | - | - | N127 | R247 | Q251 |
| RPRC015082 | D89 | D276 | N83 | C102 | C108 | - | C267 | C271 | C309 | C346 | P80 | F356 | P357 | - | D125 | R244 | P248 |
| RPRC015076 | D84 | D272 | N78 | C97 | C103 | - | C263 | C267 | C305 | C342 | T75 | S352 | - | - | D120 | R240 | Q244 |
| RPRC012664 | D21 | D207 | E15 | C34 | C39 | - | C198 | C202 | C240 | C277 | P12 | I287 | P288 | - | L56 | S174 | G178 |
| RPRC004171 | D100 | D286 | R94 | C113 | C120 | - | D277 | V281 | C319 | C357 | P91 | G367 | G368 | - | V137 | N255 | T259 |
| RPRC006028 | D92 | D278 | S86 | C105 | C112 | - | C269 | C273 | - | C342 | P83 | P352 | A353 | P354 | N129 | R247 | Q251 |
| RPRC012785 | D89 | D277 | H83 | C102 | C107 | - | C268 | C272 | C310 | C347 | P80 | S357 | L358 | Q359 | D124 | R243 | K247 |
| RPRC006759 | D99 | D288 | K93 | C112 | C119 | - | C279 | C283 | C322 | Q352 | P90 | - | - | - | N136 | I254 | T258 |
| RPRC004330 | D92 | D277 | S86 | C105 | C110 | - | C268 | C272 | C310 | C346 | P83 | L355 | K356 | - | E127 | R245 | V249 |
| RPRC014747 | D90 | D276 | N84 | C103 | C109 | - | C267 | C271 | C311 | C348 | P81 | I357 | K358 | - | D126 | R244 | K248 |
| RPRC002479 | D93 | D279 | E87 | C106 | C113 | - | C270 | C274 | C313 | C349 | P84 | Y359 | K360 | - | D130 | R248 | P252 |
| RPRC012504 | - | D82 | - | - | - | - | C73 | C77 | C117 | C163 | - | I172 | K173 | - | K8 | R50 | K54 |
| RPRC012508 | D88 | D274 | Y82 | C101 | C107 | - | C265 | C269 | C307 | C344 | P79 | S354 | Q355 | - | D124 | R242 | L246 |
| RPRC011752 | D52 | - | N46 | C65 | C71 | - | - | - | - | - | P43 | - | - | - | D88 | - | - |

| Gene | S4 | S4 | S4 | S3 | S3 | S3* | S3 | S3 | S3 | S3 | S3 | S2 | S2 | S2 | S2 | S2 | S2 | S2 | S2 | S2 |
| --- | --- | --- | --- | --- | --- | --- | --- | --- | --- | --- | --- | --- | --- | --- | --- | --- | --- | --- | --- | --- |
| Pepsin A | M74 | S281 | L282 | E75 | T139 | F173 | L174 | F179 | G279 | T280 | S281 | Y137 | G138 | T139 | G279 | T280 | T284 | Q349 | M351 | I362 |
| renin | T84 | S296 | Y297 | Q85 | T151 | P184 | F185 | F190 | G294 | A295 | S296 | Y149 | S150 | T151 | G294 | A295 | S299 | H367 | M369 | A380 |
| Cathepsin D | A77 | S299 | L300 | Q78 | S144 | T189 | F190 | F195 | G297 | T298 | S299 | Y142 | G143 | S144 | G297 | T298 | V302 | M371 | M373 | I384 |
| RPRC006698 | A70 | S282 | L283 | Q71 | S137 | T171 | F172 | F177 | G280 | T281 | S282 | Y135 | V136 | S137 | G280 | T281 | I285 | L353 | I355 | I363 |
| RPRC012786 | T69 | S281 | F282 | Q70 | T134 | P168 | F169 | P174 | G279 | T280 | S281 | Y132 | G133 | T134 | G279 | T280 | V284 | T352 | L354 | I363 |
| RPRC015079 | A71 | S283 | L284 | D72 | T137 | P171 | F172 | F177 | G281 | T282 | S283 | Y135 | G136 | T137 | G281 | T282 | I286 | S354 | L356 | I364 |
| RPRC015082 | A69 | S280 | L281 | A70 | S135 | P169 | F170 | F175 | G278 | T279 | S280 | Y133 | G134 | S135 | G278 | T279 | V283 | S351 | L353 | I362 |
| RPRC015076 | A64 | S276 | L277 | A65 | T130 | P164 | F165 | F170 | G274 | T275 | S276 | Y128 | G129 | T130 | G274 | T275 | I279 | S347 | L349 | I357 |
| RPRC012664 | A1 | S211 | L212 | E2 | T66 | P100 | F101 | L106 | G209 | T210 | S211 | Y64 | G65 | T66 | G209 | T210 | V214 | T282 | V284 | I293 |
| RPRC004171 | A80 | S290 | L291 | E81 | T147 | T181 | F182 | F187 | G288 | A289 | S290 | Y145 | V146 | T147 | G288 | A289 | I293 | A362 | M364 | I372 |
| RPRC006028 | A72 | S282 | L283 | Q73 | S139 | V173 | F174 | F179 | G280 | T281 | S282 | Y137 | G138 | S139 | G280 | T281 | A285 | L347 | L349 | I360 |
| RPRC012785 | A69 | S281 | L282 | E70 | T134 | P168 | F169 | T174 | G279 | T280 | S281 | Y132 | G133 | T134 | G279 | T280 | V284 | S352 | L354 | I365 |
| RPRC006759 | T79 | N292 | T293 | E80 | S146 | I179 | L180 | V185 | S290 | E291 | N292 | L144 | G145 | S146 | S290 | E291 | R295 | K357 | L358 | - |
| RPRC004330 | S72 | T281 | M282 | Q73 | K137 | A171 | F172 | F177 | G279 | T280 | T281 | Y135 | G136 | K137 | G279 | T280 | I284 | S351 | V353 | I361 |
| RPRC014747 | V70 | S280 | L281 | D71 | T136 | P170 | F171 | C176 | G278 | T279 | S280 | Y134 | G135 | T136 | G278 | T279 | I283 | S353 | A354 | I363 |
| RPRC002479 | V73 | S283 | L284 | Q74 | S140 | - | F173 | F178 | G281 | T282 | S283 | Y138 | G139 | S140 | G281 | T282 | I286 | S354 | M356 | L364 |
| RPRC012504 | - | S86 | L87 | - | - | - | - | - | G84 | T85 | S86 | Y16 | - | - | G84 | T85 | I89 | S268 | A269 | I178 |
| RPRC012508 | A68 | S278 | M279 | A69 | T134 | P168 | F169 | F174 | G276 | T277 | S278 | Y132 | G133 | T134 | G276 | T277 | I281 | S349 | A351 | I360 |
| RPRC011752 | V32 | - | - | D33 | T98 | - | - | - | - | - | - | Y96 | G97 | T98 | - | - | - | - | - | - |

| Gene | S1 | S1 | S1 | S1 | S1 | S1 | S1 | S1 | S1 | S1’ | S1’ | S1’ | S1’ | S1’ | S2’ | S2’ | S2’ | S2’ | S2’ |
| --- | --- | --- | --- | --- | --- | --- | --- | --- | --- | --- | --- | --- | --- | --- | --- | --- | --- | --- | --- |
| Pepsin A | V92 | D94 | G96 | T136 | Y137 | L174 | F179 | I182 | D277 | G96 | S97 | T136 | Y137 | T280 | T136 | Y137 | G138 | Y251 | L353 |
| renin | V102 | D104 | G106 | R148 | Y149 | F185 | F190 | V193 | D292 | G106 | S107 | R148 | Y149 | A295 | R148 | Y149 | S150 | V266 | I371 |
| Cathepsin D | V95 | D97 | G99 | H141 | Y142 | F190 | F195 | I198 | D295 | G99 | S100 | H141 | Y142 | T298 | H141 | Y142 | G143 | Y269 | I375 |
| RPRC006698 | I88 | D90 | G92 | R134 | Y135 | F172 | F177 | I180 | D278 | G92 | S93 | R134 | Y135 | T281 | R134 | Y135 | V136 | Y252 | L357 |
| RPRC012786 | I87 | D89 | G91 | V131 | Y132 | F169 | P174 | I177 | D277 | G91 | S92 | V131 | Y132 | T280 | V131 | Y132 | G133 | Y249 | K356 |
| RPRC015079 | V89 | D91 | G93 | A134 | Y135 | F172 | F177 | I180 | D279 | G93 | S94 | A134 | Y135 | T282 | A134 | Y135 | G136 | Y253 | G358 |
| RPRC015082 | V87 | D89 | G91 | T132 | Y133 | F170 | F175 | I178 | D276 | G91 | S92 | T132 | Y133 | T279 | T132 | Y133 | G134 | Y250 | K355 |
| RPRC015076 | V82 | D84 | G86 | A127 | Y128 | F165 | F170 | I173 | D272 | G86 | S87 | A127 | Y128 | T275 | A127 | Y128 | G129 | Y246 | G351 |
| RPRC012664 | I19 | D21 | G23 | E63 | Y64 | F101 | L106 | I109 | D207 | G23 | S24 | E63 | Y64 | T210 | E63 | Y64 | G65 | F180 | N286 |
| RPRC004171 | V98 | D100 | G102 | S144 | Y145 | F182 | F187 | V190 | D286 | G102 | S103 | S144 | Y145 | A289 | S144 | Y145 | V146 | F261 | L366 |
| RPRC006028 | V90 | D92 | G94 | Q136 | Y137 | F174 | F179 | I182 | D278 | G94 | S95 | Q136 | Y137 | T281 | Q136 | Y137 | G138 | Y253 | V351 |
| RPRC012785 | I87 | D89 | G91 | V131 | Y132 | F169 | T174 | I177 | D277 | G91 | S92 | V131 | Y132 | T280 | V131 | Y132 | G133 | Y249 | E356 |
| RPRC006759 | V97 | D99 | A101 | K143 | L144 | L180 | V185 | V188 | D288 | A101 | W102 | K143 | L144 | E291 | K143 | L144 | G145 | V260 | - |
| RPRC004330 | L90 | D92 | G94 | I134 | Y135 | F172 | F177 | I180 | D277 | G94 | S95 | I134 | Y135 | T280 | I134 | Y135 | G136 | K251 | E354 |
| RPRC014747 | I88 | D90 | G92 | V133 | Y134 | F171 | C176 | L179 | D276 | G92 | S93 | V133 | Y134 | T279 | V133 | Y134 | G135 | H250 | S356 |
| RPRC002479 | V91 | D93 | G95 | A137 | Y138 | F173 | F178 | L181 | D279 | G95 | S96 | A137 | Y138 | T282 | A137 | Y138 | G139 | Y254 | D358 |
| RPRC012504 | - | - | - | P15 | Y16 | - | - | - | D82 | - | - | P15 | Y16 | T85 | P15 | Y16 | - | H56 | S171 |
| RPRC012508 | I86 | D88 | G90 | S131 | Y132 | F169 | F174 | I177 | D274 | G90 | S91 | S131 | Y132 | T277 | S131 | Y132 | G133 | Y248 | Y353 |
| RPRC011752 | I50 | D52 | G54 | V95 | Y96 | - | - | - | - | G54 | S55 | V95 | Y96 | - | V95 | Y96 | G97 | - | - |
