# Supplementary material for: Genome Wide Mapping of Peptidases in Rhodnius prolixus: Identification of Protease Gene Duplications, Horizontally Transferred Proteases and Analysis of Peptidase A1 Structures, with Considerations on Their Role in the Evolution of Hematophagy in Triatominae
Source: Front Physiol. 2017 Dec 12;8:1051. doi: 10.3389/fphys.2017.01051 (PMC5736985; doi:10.3389/fphys.2017.01051)
Supplement: Supplementary file 22 [file Table12.DOCX]

Supplementary Material

Genome wide mapping of peptidases in *Rhodnius prolixus*: identification of protease gene duplications, horizontally transferred proteases and analysis of peptidase A1 structures, with considerations on their role in the evolution of hematophagy in Triatominae

**Bianca Santos Henriques, Bruno Gomes, Caroline da Silva Moraes, Samara Graciane Costa, Rafael Dias Mesquita, Viv Maureen Dillon, Eloi de Souza Garcia, Patricia Azambuja, Roderick James Dillon, Fernando Ariel Genta***

*** Correspondence:** Corresponding Author: genta@ioc.fiocruz.br or [gentafernando@gmail.com](mailto:gentafernando@gmail.com)

**Supplementary Table 12.**  Putative Glycosilation sites in *Rhodnius prolixus* peptidases of Family A1. Predictions were performed with NetNGlyc and NetOGlyc (see Material and Methods for details).

| Gene | Frequency | | Residue location | |
| --- | --- | --- | --- | --- |
|  | O-glycosylation | N-glycosylation | O-glycosylated | N-glycosylated |
| A01.009_sp |  | 2 |  | N134 N263 |
| gi\|5572822 | 8 | 2 | S31 T35 S37 S56 T63 S125 S129 T130 | N134 N263 |
| gi\|4263668 | 6 | 2 | S31 T35 S56 T63 S125 S129 | N134 N263 |
| gi\|3322647 | 6 | 2 | S31 T35 S56 T63 S125 S129 | N134 N263 |
| gi\|1609042 | 6 | 2 | S31 T35 S56 T63 S125 S129 | N134 N263 |
| gi\|1706496 | 5 | 2 | S31 T35 S56 S125 S129 | N134 N263 |
| gi\|3555661 | 6 | 2 | S31 T35 S56 T63 S125 S129 | N172 N301 |
| gi\|5444836 | 6 | 2 | S31 T35 S56 T63 S125 S129 | N134 N263 |
| gi\|2962190 | 3 | 1 | S31 T35 S56 | N243 |
| gi\|3974902 | 7 | 4 | S31 T35 S56 S61 T63 S125 S129 | N134 N263 N511 N578 |
| RPRC006698 | 0 | 2 | - | N127 N239 |
| RPRC012786 | 0 | 1 | - | N74 |
| RPRC015079 | 2 | 4 | S119 S122 | N85 N158 N239 N381 |
| RPRC015082 | 3 | 1 | S113 S116 S117 | N83 |
| RPRC015076 | 4 | 4 | S49 S100 S112 S115 | N78 N151 N232 N374 |
| RPRC012664 | 4 | 0 | S47 T48 S50 S51 | - |
| RPRC004171 | 8 | 4 | S38 T56 T60 S61 T129 S131 S132 T133 | N168 N247 N255 N386 |
| RPRC006028 | 6 | 0 | S32 S43 S120 S121 S122 S123 | - |
| RPRC012785 | 6 | 2 | S104 T105 S106 S118 T119 T120 | N117 N256 |
| RPRC006759 | 5 | 6 | S123 S128 S130 S131 T132 | N24 N136 N164 N167 N224 N248 |
| RPRC004330 | 4 | 3 | S57 S121 S122 T123 | N153 N263 N324 |
| RPRC014747 | 7 | 5 | T47 S48 S106 S117 S120 S121 T122 | N84 N157 N216 N231 N380 |
| RPRC002479 | 7 | 3 | T57 S110 S121 S122 S124 T125 S188 | N21 N120 N161 |
| RPRC012504 | 0 | 3 | - | N22 N37 N195 |
| RPRC012508 | 6 | 2 | S48 S49 T50 S116 S119 T120 | N155 N295 |
| RPRC011752 | 0 | 1 | - | N46 |
