# Supplementary material for: Genome Wide Mapping of Peptidases in Rhodnius prolixus: Identification of Protease Gene Duplications, Horizontally Transferred Proteases and Analysis of Peptidase A1 Structures, with Considerations on Their Role in the Evolution of Hematophagy in Triatominae
Source: Front Physiol. 2017 Dec 12;8:1051. doi: 10.3389/fphys.2017.01051 (PMC5736985; doi:10.3389/fphys.2017.01051)
Supplement: Supplementary file 23 [file Table13.DOCX]

Supplementary Material

Genome wide mapping of peptidases in *Rhodnius prolixus*: identification of protease gene duplications, horizontally transferred proteases and analysis of peptidase A1 structures, with considerations on their role in the evolution of hematophagy in Triatominae

**Bianca Santos Henriques, Bruno Gomes, Caroline da Silva Moraes, Samara Graciane Costa, Rafael Dias Mesquita, Viv Maureen Dillon, Eloi de Souza Garcia, Patricia Azambuja, Roderick James Dillon, Fernando Ariel Genta***

*** Correspondence:** Corresponding Author: genta@ioc.fiocruz.br or [gentafernando@gmail.com](mailto:gentafernando@gmail.com)

**Supplementary Table 13.**  Frequencies of exposed basic residues in A1 peptidase sequences from *Rhodnius prolixus* and their human homologs Pepsin and Cathepsin D. Exposed basic residues were assigned in human crystallographic structures (PDB files 1LYA and 3UTL) and in homology-based structural models of *R. prolixus* sequences.

| Gene | Lys | Arg | Total |
| --- | --- | --- | --- |
| Pepsin A | 0 | 3 | 3 |
| Cathepsin D | 20 | 8 | 28 |
| RPRC006698 | 25 | 7 | 32 |
| RPRC012786 | 18 | 12 | 40 |
| RPRC015079 | 21 | 14 | 35 |
| RPRC015082 | 20 | 9 | 29 |
| RPRC015076 | 18 | 15 | 33 |
| RPRC012664 | 19 | 5 | 24 |
| RPRC004171 | 26 | 15 | 41 |
| RPRC006028 | 23 | 9 | 32 |
| RPRC012785 | 25 | 16 | 41 |
| RPRC006759 | 22 | 11 | 33 |
| RPRC004330 | 23 | 10 | 33 |
| RPRC014747 | 27 | 15 | 42 |
| RPRC002479 | 29 | 7 | 36 |
| RPRC012504 | 21 | 6 | 27 |
| RPRC012508 | 19 | 16 | 35 |
