# Supplementary material for: Genome Wide Mapping of Peptidases in Rhodnius prolixus: Identification of Protease Gene Duplications, Horizontally Transferred Proteases and Analysis of Peptidase A1 Structures, with Considerations on Their Role in the Evolution of Hematophagy in Triatominae
Source: Front Physiol. 2017 Dec 12;8:1051. doi: 10.3389/fphys.2017.01051 (PMC5736985; doi:10.3389/fphys.2017.01051)
Supplement: Supplementary file 24 [file Table14.DOCX]

Supplementary Material

Genome wide mapping of peptidases in *Rhodnius prolixus*: identification of protease gene duplications, horizontally transferred proteases and analysis of peptidase A1 structures, with considerations on their role in the evolution of hematophagy in Triatominae

**Bianca Santos Henriques, Bruno Gomes, Caroline da Silva Moraes, Samara Graciane Costa, Rafael Dias Mesquita, Viv Maureen Dillon, Eloi de Souza Garcia, Patricia Azambuja, Roderick James Dillon, Fernando Ariel Genta***

*** Correspondence:** Corresponding Author: genta@ioc.fiocruz.br or [gentafernando@gmail.com](mailto:gentafernando@gmail.com)

**Supplementary Table 14.**  Comparison of protease gene mapping in the transcriptome (Ribeiro *et al.*, 2014) with the genome of *Rhodnius prolixus* (this work).

| Protease Family | Identifier | |
| --- | --- | --- |
|  | Genome | Transcriptome |
| A1 | RPRC002478 | [RP-10596](http://exon.niaid.nih.gov/transcriptome/R_prolixus_454/S2/links/pep/Rp-10596-pep.txt) |
|  | RPRC002696 | - |
|  | RPRC004171 | [RP-6421](http://exon.niaid.nih.gov/transcriptome/R_prolixus_454/S2/links/pep/RP-6421-pep.txt) |
|  | RPRC004330 | [RP-7417](http://exon.niaid.nih.gov/transcriptome/R_prolixus_454/S2/links/pep/RP-7417-pep.txt) |
|  | RPRC006028 | [RP-1760](http://exon.niaid.nih.gov/transcriptome/R_prolixus_454/S2/links/pep/Rp-1760-pep.txt) |
|  | RPRC006698 | [RP-5007](http://exon.niaid.nih.gov/transcriptome/R_prolixus_454/S2/links/pep/RP-5007-pep.txt) |
|  | RPRC006759 | [RP-3415](http://exon.niaid.nih.gov/transcriptome/R_prolixus_454/S2/links/pep/RP-3415-pep.txt) |
|  | RPRC008989 | [RP-2814](http://exon.niaid.nih.gov/transcriptome/R_prolixus_454/S2/links/pep/Rp-2814-pep.txt) |
|  | RPRC010954 | [RP-7417](http://exon.niaid.nih.gov/transcriptome/R_prolixus_454/S2/links/pep/RP-7417-pep.txt) |
|  | RPRC011752 | [RP-2814](http://exon.niaid.nih.gov/transcriptome/R_prolixus_454/S2/links/pep/Rp-2814-pep.txt) |
|  | RPRC012487 | [RP-2814](http://exon.niaid.nih.gov/transcriptome/R_prolixus_454/S2/links/pep/Rp-2814-pep.txt) |
|  | RPRC012508 | [RP-8066](http://exon.niaid.nih.gov/transcriptome/R_prolixus_454/S2/links/pep/RP-8066-pep.txt) |
|  | RPRC012664 | - |
|  | RPRC012785 | [RP-2217](http://exon.niaid.nih.gov/transcriptome/R_prolixus_454/S2/links/pep/Rp-2217-pep.txt) |
|  | RPRC012786 | [RP-39441](http://exon.niaid.nih.gov/transcriptome/R_prolixus_454/S2/links/pep/RP-39441-pep.txt) |
|  | RPRC014747 | [RP-2814](http://exon.niaid.nih.gov/transcriptome/R_prolixus_454/S2/links/pep/Rp-2814-pep.txt) |
|  | RPRC015076 | [RP-6846](http://exon.niaid.nih.gov/transcriptome/R_prolixus_454/S2/links/pep/Rp-6846-pep.txt) |
|  | RPRC015079 | [RP-6850](http://exon.niaid.nih.gov/transcriptome/R_prolixus_454/S2/links/pep/Rp-6850-pep.txt) |
|  | RPRC015082 | [RP-82226](http://exon.niaid.nih.gov/transcriptome/R_prolixus_454/S2/links/pep/RP-82226-pep.txt) |
| C1 | RPRC000294 | [RP-4483](http://exon.niaid.nih.gov/transcriptome/R_prolixus_454/S2/links/pep/RP-4483-pep.txt) |
|  | RPR006917-6907 | [RP-428](http://exon.niaid.nih.gov/transcriptome/R_prolixus_454/S2/links/pep/Rp-428-pep.txt) |
|  | RPRC000205 | [RP-1305](http://exon.niaid.nih.gov/transcriptome/R_prolixus_454/S2/links/pep/RP-1305-pep.txt) |
|  | RPRC000309 | - |
|  | RPRC000405 | - |
|  | RPRC002593 | [RP-2313](http://exon.niaid.nih.gov/transcriptome/R_prolixus_454/S2/links/pep/Rp-2313-pep.txt) |
|  | RPRC002640 | [RP-5910](http://exon.niaid.nih.gov/transcriptome/R_prolixus_454/S2/links/pep/RP-5910-pep.txt) |
|  | RPRC005321 | [RP-2313](http://exon.niaid.nih.gov/transcriptome/R_prolixus_454/S2/links/pep/Rp-2313-pep.txt) |
|  | RPRC005322 | - |
|  | RPRC008250 | - |
|  | RPRC010398 | [RP-35466](http://exon.niaid.nih.gov/transcriptome/R_prolixus_454/S2/links/pep/Rp-35466-pep.txt) |
|  | RPRC013182 | [RP-10924](http://exon.niaid.nih.gov/transcriptome/R_prolixus_454/S2/links/pep/Rp-10924-pep.txt) |
|  | RPRC013528 | RP-25907* |
|  | RPRC015288 | [RP-34337](http://exon.niaid.nih.gov/transcriptome/R_prolixus_454/S2/links/pep/RP-34337-pep.txt) |
|  | RPRC015289 | [RP-2246](http://exon.niaid.nih.gov/transcriptome/R_prolixus_454/S2/links/pep/RP-2246-pep.txt) |
|  | RPRC015290 | - |
|  | RPRC015299 | RP-3723* |
| C2 | RPRC013347 | [RP-94045](http://exon.niaid.nih.gov/transcriptome/R_prolixus_454/S2/links/pep/RP-94045-pep.txt)* |
|  | RPRC002326 | - |
|  | RPRC007632 | - |
|  | RPRC012594 | RP-11100* |
|  | RPRC012930 | - |
|  | RPRC013350 | - |
|  | RPRC013353 | - |
|  | RPRC013355 | - |
|  | RPRC013605 | - |
|  | RPRC013606 | - |
|  | RPRC014368 | - |
|  | RPRC015123 | RP-11100* |
| M17 | RPRC011316 | [RP-21838](http://exon.niaid.nih.gov/transcriptome/R_prolixus_454/S2/links/pep/RP-21838-pep.txt)* |
|  | RPRC000644 | - |
|  | RPRC000886 | - |
|  | RPRC003574 | [RP-10247](http://exon.niaid.nih.gov/transcriptome/R_prolixus_454/S2/links/pep/RP-10247-pep.txt)* |
|  | RPRC008281 | - |
|  | RPRC009154 | [RP-12371](http://exon.niaid.nih.gov/transcriptome/R_prolixus_454/S2/links/pep/Rp-12371-pep.txt)* |
|  | RPRC012383 | - |
|  | RPRC012689 | [RP-23956](http://exon.niaid.nih.gov/transcriptome/R_prolixus_454/S2/links/pep/Rp-23956-pep.txt)* |
|  | RPRC012692 | - |
|  | RPRC013170 | [RP-8740](http://exon.niaid.nih.gov/transcriptome/R_prolixus_454/S2/links/pep/Rp-8740-pep.txt)* |
|  | RPRC014323 | - |
|  | RPRC014324 | [RP-99958](http://exon.niaid.nih.gov/transcriptome/R_prolixus_454/S2/links/pep/RP-99958-pep.txt)* |
|  | RPRC014856 | - |
| S24 | RPRC002798 | RP-15903* |
|  | RPRC005865 | - |
|  | RPRC010630 | - |
| S29 | RPRC004810 | - |
|  | RPRC013821 | - |
| M74 | RPRC003168 | - |
