# Supplementary material for: Genome Wide Mapping of Peptidases in Rhodnius prolixus: Identification of Protease Gene Duplications, Horizontally Transferred Proteases and Analysis of Peptidase A1 Structures, with Considerations on Their Role in the Evolution of Hematophagy in Triatominae
Source: Front Physiol. 2017 Dec 12;8:1051. doi: 10.3389/fphys.2017.01051 (PMC5736985; doi:10.3389/fphys.2017.01051)
Supplement: Supplementary file 25 [file Table15.DOCX]

Supplementary Material

Genome wide mapping of peptidases in *Rhodnius prolixus*: identification of protease gene duplications, horizontally transferred proteases and analysis of peptidase A1 structures, with considerations on their role in the evolution of hematophagy in Triatominae

**Bianca Santos Henriques, Bruno Gomes, Caroline da Silva Moraes, Samara Graciane Costa, Rafael Dias Mesquita, Viv Maureen Dillon, Eloi de Souza Garcia, Patricia Azambuja, Roderick James Dillon, Fernando Ariel Genta***

*** Correspondence:** Corresponding Author: genta@ioc.fiocruz.br or [gentafernando@gmail.com](mailto:gentafernando@gmail.com)

**Supplementary Table 15.**  Best 2 matches for blastp of selected *Rhodnius prolixus* sequences of protease family C2 against sequences of human calpains.

| *R. prolixus* ID | Human match, Description | Query cover | E-value | Identity | Accession |
| --- | --- | --- | --- | --- | --- |
| RPRC002326 | calpain-3 isoform c | 96% | 9e-158 | 39% | EAW92550.1 |
|  | calpain-8 | 98% | 2e-157 | 41% | NP_001137434.1 |
| RPRC012584 | calpain-8 | 99% | 1e-148 | 39% | NP_001137434.1 |
|  | calpain-3 isoform c | 96% | 5e-148 | 38% | NP_775110.1 |
| RPRC013353 | calpain 9, isof.CRA_c | 74% | 2e-20 | 50% | EAW69922.1 |
|  | calpain-8 | 76% | 8e-17 | 45% | NP_001137434.1 |
| RPRC013606 | calpain-3 isoform c | 97% | 3e-154 | 38% | NP_775110.1 |
|  | calpain-8 | 97% | 6e-142 | 38% | NP_001137434.1 |
| RPRC013605 | calpain 9, isof. CRA_c | 87% | 2e-19 | 47% | EAW69922.1 |
|  | calpain-5 | 83% | 2e-15 | 46% | NP_004046.2 |
| RPRC015123 | calpain-3 isoform b | 96% | 2e-162 | 36% | NP_077320.1 |
|  | Calpain 8 | 98% | 8e-160 | 39% | AAI57894.1 |
| RPRC013355 | calpain 9, isof. CRA_c | 87% | 4e-55 | 40% | EAW69922.1 |
|  | calpain 3 trans. var. 11 | 95% | 5e-49 | 36% | ACI63188.1 |
